# Supplementary material for: Facial Synthesis and Bioevaluation of Well-Defined OEGylated Betulinic Acid-Cyclodextrin Conjugates for Inhibition of Influenza Infection
Source: Molecules. 2022 Feb 9;27(4):1163. doi: 10.3390/molecules27041163 (PMC8880671; doi:10.3390/molecules27041163)

Supporting Information

Facial Synthesis and Bioevaluation of Well-Defined OEGylated Betulinic Acid-Cyclodextrin Conjugates for Inhibition of Influenza Entry

Yingying Chen,[1,†] Xinchen Wang,[1,†] Xinyuan Ma,[1] Shuobin Liang,[1] Qianqian Gao,[1] Elena V. Tretyakova,[3] Yongmin Zhang,[4] Demin Zhou,[1,2] Sulong Xiao[1,*]

[1]State Key Laboratory of Natural and Biomimetic Drugs, School of Pharmaceutical Sciences, Peking University, Beijing 100191, China

[2]Institute of Chemical Biology, Shenzhen Bay Laboratories, Shenzhen 518132, China

[3]Ufa Institute of Chemistry of the Ufa Federal Research Centre of the Russian Academy of Sciences, 71 Prospect Oktyabrya, 450054, Ufa, Russian Federation

[4]Sorbonne Université, CNRS, Institut Parisien de Chimie Moléculaire, UMR 8232, 4 place Jussieu, 75005 Paris, France

1. **Figure S1**. Cytotoxicity of multivalent BA-CD conjugates to MDCK cells S4

2. **Figure S2**. Binding sensorgrams for conjugates **81** and **83** interaction with influenza HA protein. S4

3. Selected NMR, HRMS and HRMS or MALDI-TOF MS spectra S5

Spectra	Page
^{1}H NMR of compound **34**	S5
^{13}C NMR of compound **34**	S5
HRMS of compound **34**	S6
^{1}H NMR of compound **35**	S6
^{13}C NMR of compound **35**	S7
HRMS of compound **35**	S7
^{1}H NMR of compound **36**	S8
^{13}C NMR of compound **36**	S8
HRMS of compound **36**	S9
^{1}H NMR of compound **37**	S9
^{13}C NMR of compound **37**	S10
HRMS of compound **37**	S10
^{1}H NMR of compound **38**	S11
^{13}C NMR of compound **38**	S11
HRMS of compound **38**	S12
^{1}H NMR of compound **51**	S12
^{13}C NMR of compound **51**	S13

MALDI-TOF of compound **51**	S13
^{1}H NMR of compound **52**	S14
^{13}C NMR of compound **52**	S14
MALDI-TOF of compound **52**	S15
^{1}H NMR of compound **53**	S15
^{13}C NMR of compound **53**	S16
MALDI-TOF of compound **53**	S16
^{1}H NMR of compound **54**	S17
^{13}C NMR of compound **54**	S17
MALDI-TOF of compound **54**	S18
^{1}H NMR of compound **55**	S18
^{13}C NMR of compound **55**	S19
MALDI-TOF of compound **55**	S19
^{1}H NMR of compound **56**	S20
^{13}C NMR of compound **56**	S20
MALDI-TOF of compound **56**	S21
^{1}H NMR of compound **57**	S21
^{13}C NMR of compound **57**	S22
MALDI-TOF of compound **57**	S22
^{1}H NMR of compound **58**	S23
^{13}C NMR of compound **58**	S23
MALDI-TOF of compound **58**	S24
^{1}H NMR of compound **59**	S24
^{13}C NMR of compound **59**	S25
MALDI-TOF of compound **59**	S25
^{1}H NMR of compound **60**	S26
^{13}C NMR of compound **60**	S26
MALDI-TOF of compound **60**	S27

^{1}H NMR of compound **61**	S27
^{13}C NMR of compound **61**	S28
MALDI-TOF of compound **61**	S28
^{1}H NMR of compound **62**	S29
^{13}C NMR of compound **62**	S29
MALDI-TOF of compound **62**	S30
^{1}H NMR of compound **63**	S30
^{13}C NMR of compound **63**	S45
MALDI-TOF of compound **63**	S31
^{1}H NMR of compound **64**	S32
^{13}C NMR of compound **64**	S32
MALDI-TOF of compound **64**	S33
^{1}H NMR of compound **65**	S33
^{13}C NMR of compound **65**	S34
MALDI-TOF of compound **65**	S34
^{1}H NMR of compound **66**	S35
^{13}C NMR of compound **66**	S35
MALDI-TOF of compound **66**	S36
^{1}H NMR of compound **67**	S36
^{13}C NMR of compound **67**	S37
MALDI-TOF of compound **67**	S37
^{1}H NMR of compound **68**	S38
^{13}C NMR of compound **68**	S38
MALDI-TOF of compound **68**	S39
^{1}H NMR of compound **69**	S39
^{13}C NMR of compound **69**	S40
MALDI-TOF of compound **69**	S40
^{1}H NMR of compound **70**	S41

^{13}C NMR of compound **70**	S41
MALDI-TOF of compound **70**	S42
^{1}H NMR of compound **71**	S42
^{13}C NMR of compound **71**	S43
MALDI-TOF of compound **71**	S43
^{1}H NMR of compound **72**	S44
^{13}C NMR of compound **72**	S44
MALDI-TOF of compound **72**	S59
^{1}H NMR of compound **73**	S45
^{13}C NMR of compound **73**	S46
MALDI-TOF of compound **73**	S46
^{1}H NMR of compound **74**	S47
^{13}C NMR of compound **74**	S47
MALDI-TOF of compound **74**	S48
^{1}H NMR of compound **75**	S48
^{13}C NMR of compound **75**	S49
MALDI-TOF of compound **75**	S49
^{1}H NMR of compound **76**	S50
^{13}C NMR of compound **76**	S50
MALDI-TOF of compound **76**	S51
^{1}H NMR of compound **77**	S51
^{13}C NMR of compound **77**	S52
MALDI-TOF of compound **77**	S52
^{1}H NMR of compound **78**	S53
^{13}C NMR of compound **78**	S53
MALDI-TOF of compound **78**	S54

^{1}H NMR of compound **79**	S54
^{13}C NMR of compound **79**	S55
MALDI-TOF of compound **79**	S55
^{1}H NMR of compound **80**	S56
^{13}C NMR of compound **80**	S56
MALDI-TOF of compound **80**	S57
^{1}H NMR of compound **81**	S57
^{13}C NMR of compound **81**	S58
MALDI-TOF of compound **81**	S58
^{1}H NMR of compound **82**	S59
^{13}C NMR of compound **82**	S59
MALDI-TOF of compound **82**	S60
^{1}H NMR of compound **83**	S60
^{13}C NMR of compound **83**	S61
MALDI-TOF of compound **83**	S61
^{1}H NMR of compound **84**	S62
^{13}C NMR of compound **84**	S62
MALDI-TOF of compound **84**	S63
^{1}H NMR of compound **85**	S63
^{13}C NMR of compound **85**	S64
MALDI-TOF of compound **85**	S64
^{1}H NMR of compound **86**	S65
^{13}C NMR of compound **86**	S65
MALDI-TOF of compound **86**	S66

1. **Figure S1**.

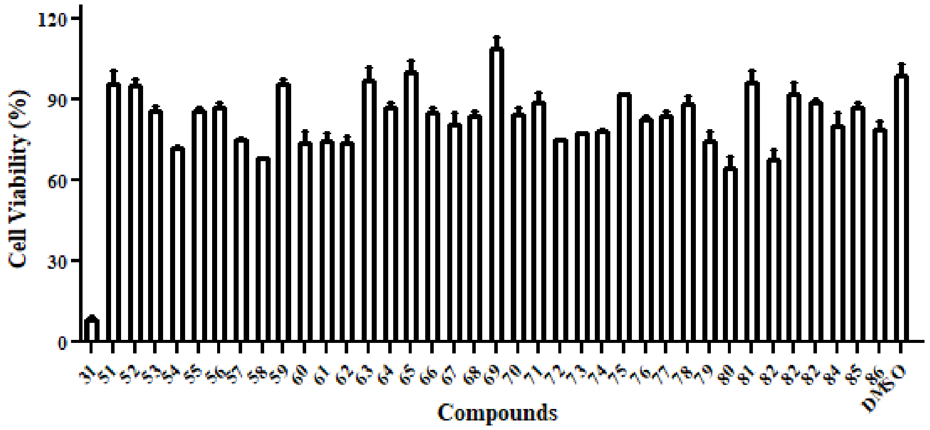

Figure S1. Cytotoxicity of multivalent BA-CD conjugates to MDCK cells. The cell viability of MDCK cells was analyzed at 36 h of incubation in the presence of BA (**31**) and its CD conjugates **51-86** at concentration of 100 μM. The CellTiter-Glo Luminescent cell viability assay kit was used. 1% DMSO acted as negative control. Error bars indicate the mean ± SD of three independent experiments.

2. **Figure S2**.

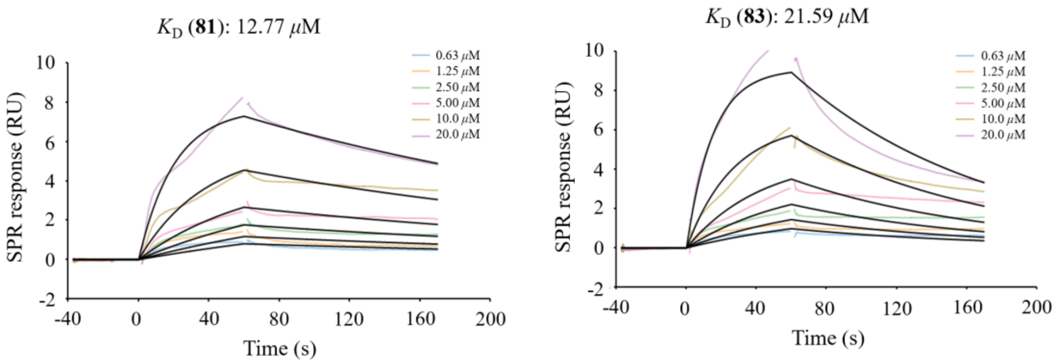

Figure S2. Binding sensorgrams for conjugates **81** and **83** interaction with influenza HA protein. The HA protein was immobilized on a CM5 sensor chip by the standard amine-coupling procedure. Different concentrations of conjugates **81** and **83** were flowed over the chip surface for a contact time of 60 s and a dissociation time of 180 s, respectively. K_D values were labeled in the graphs.

3. Selected NMR, HRMS and MALDI-TOF spectra

^{1}H NMR of compound **34**

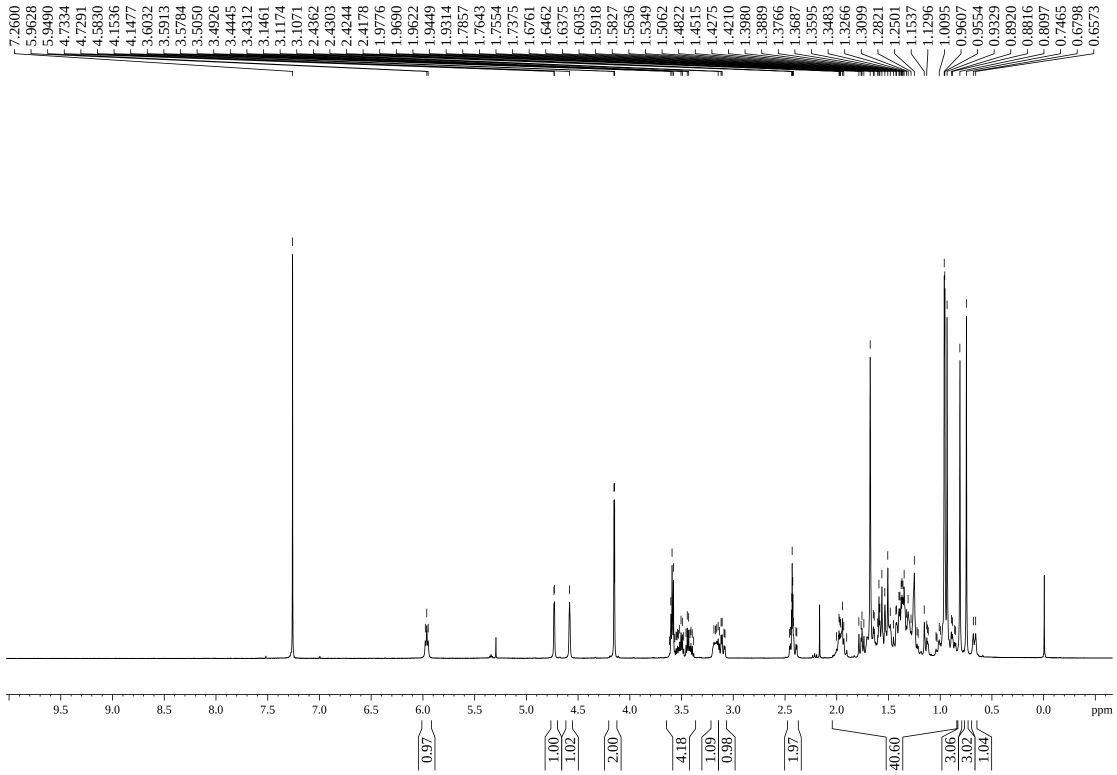

^{13}C NMR of compound **34**

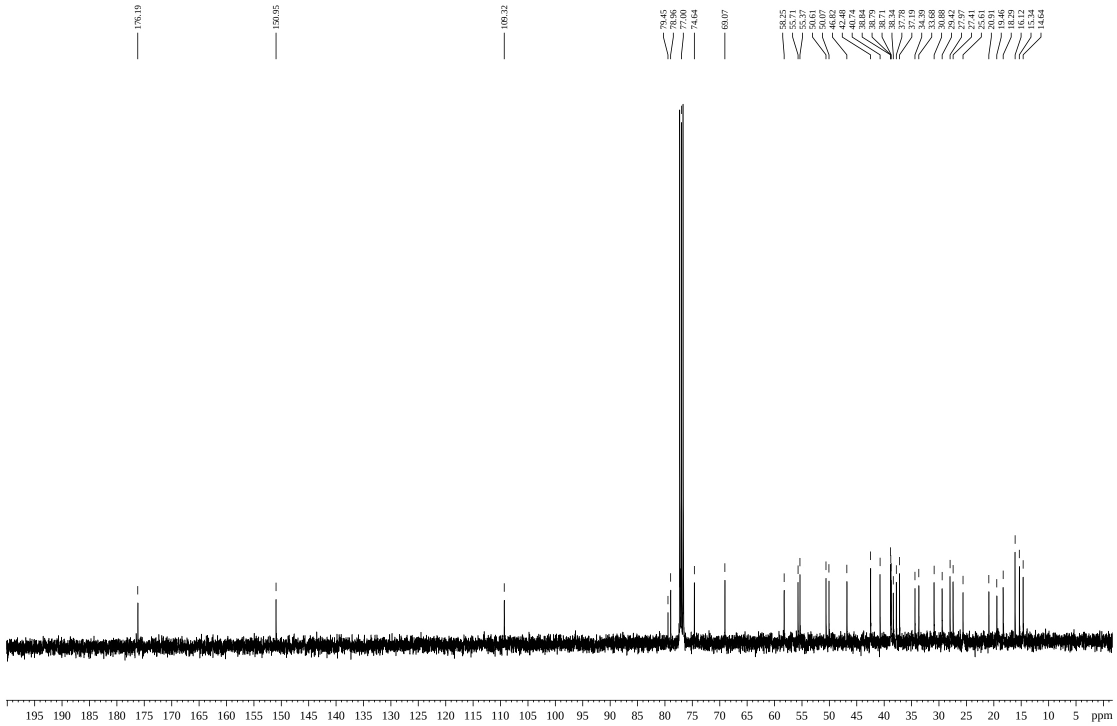

HRMS of compound **34**

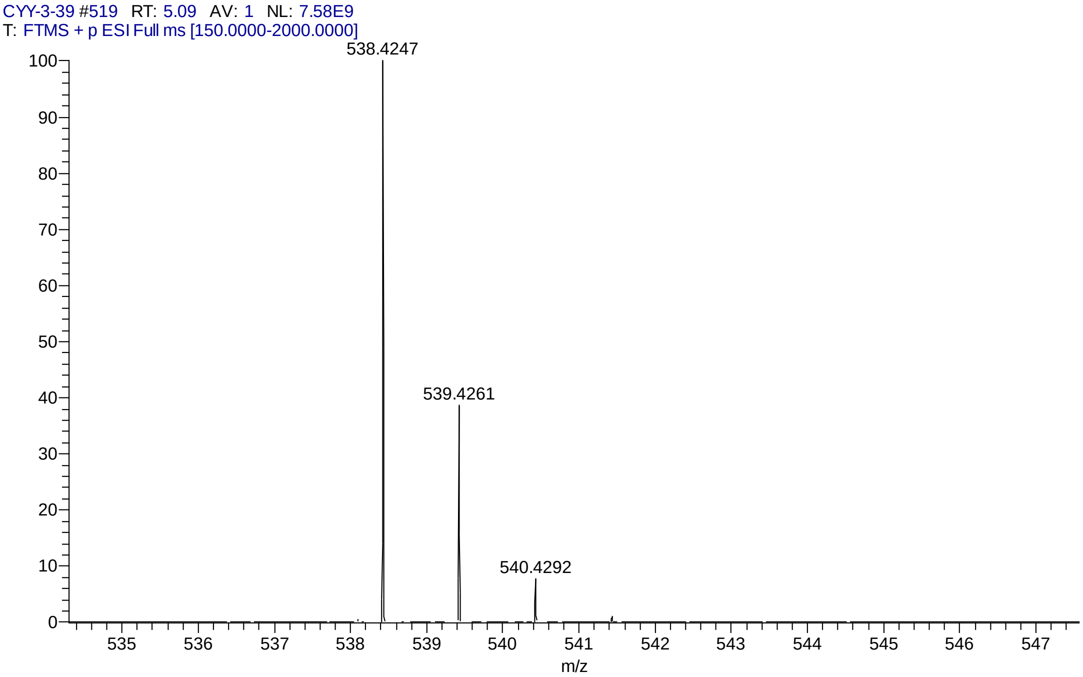

^{1}H NMR of compound **35**

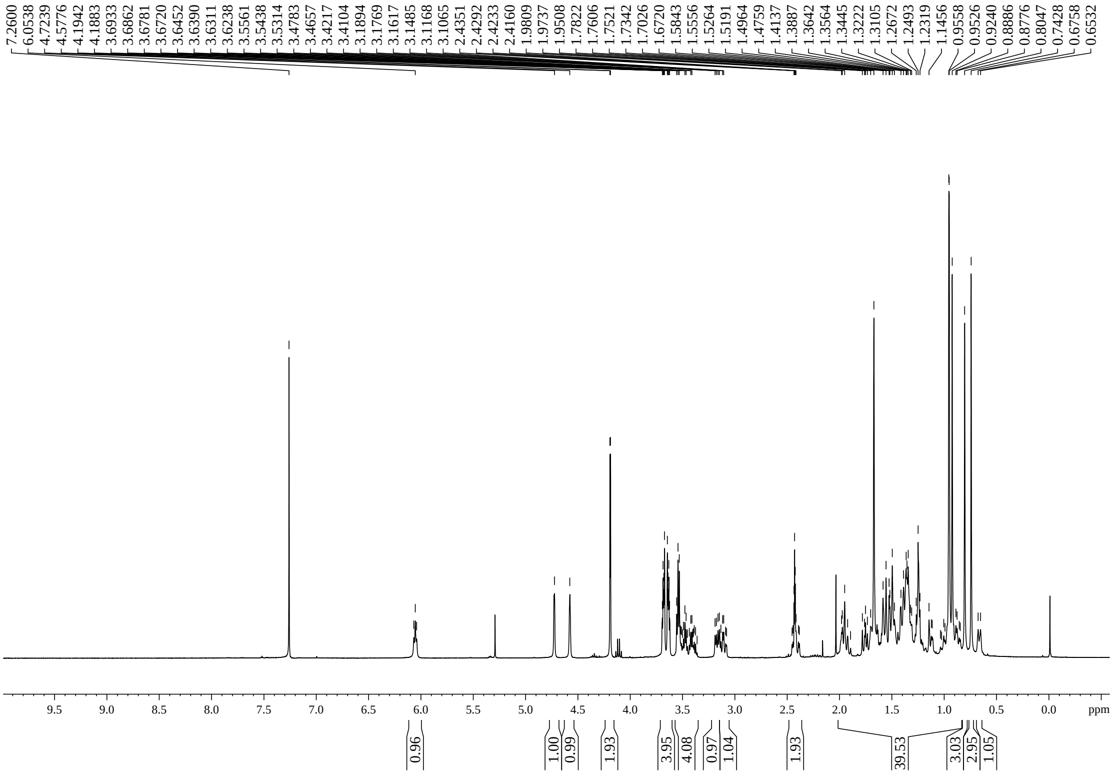

^{13}C NMR of compound **35**

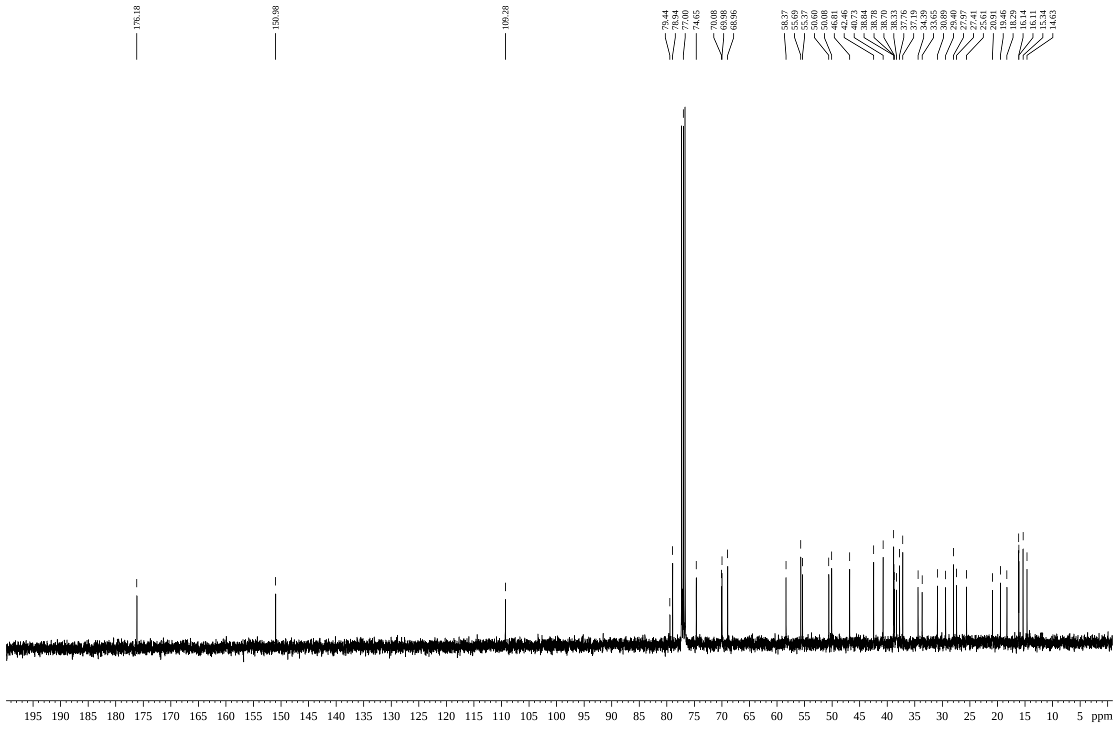

HRMS of compound **35**

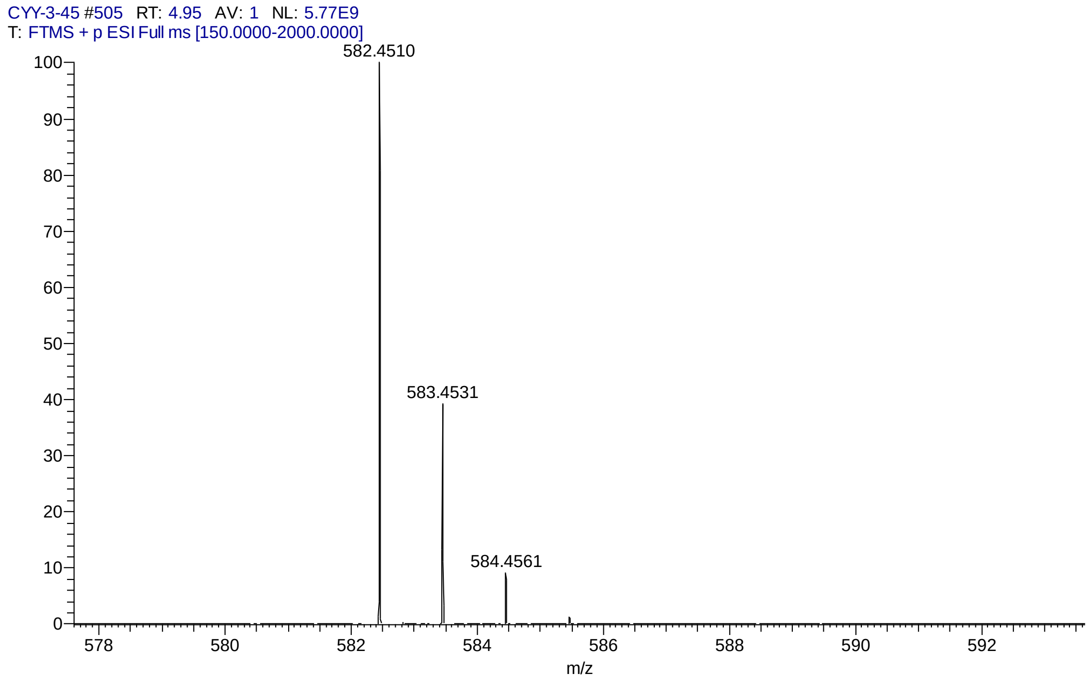

^{1}H NMR of compound **36**

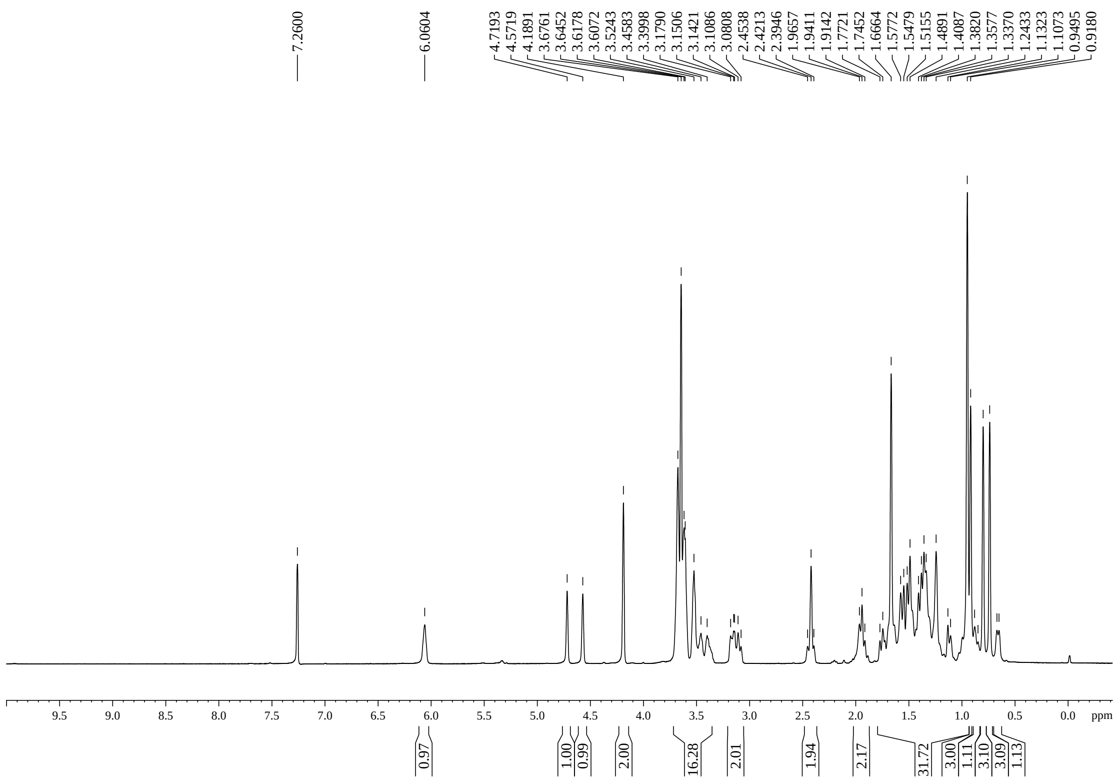

^{13}C NMR of compound **36**

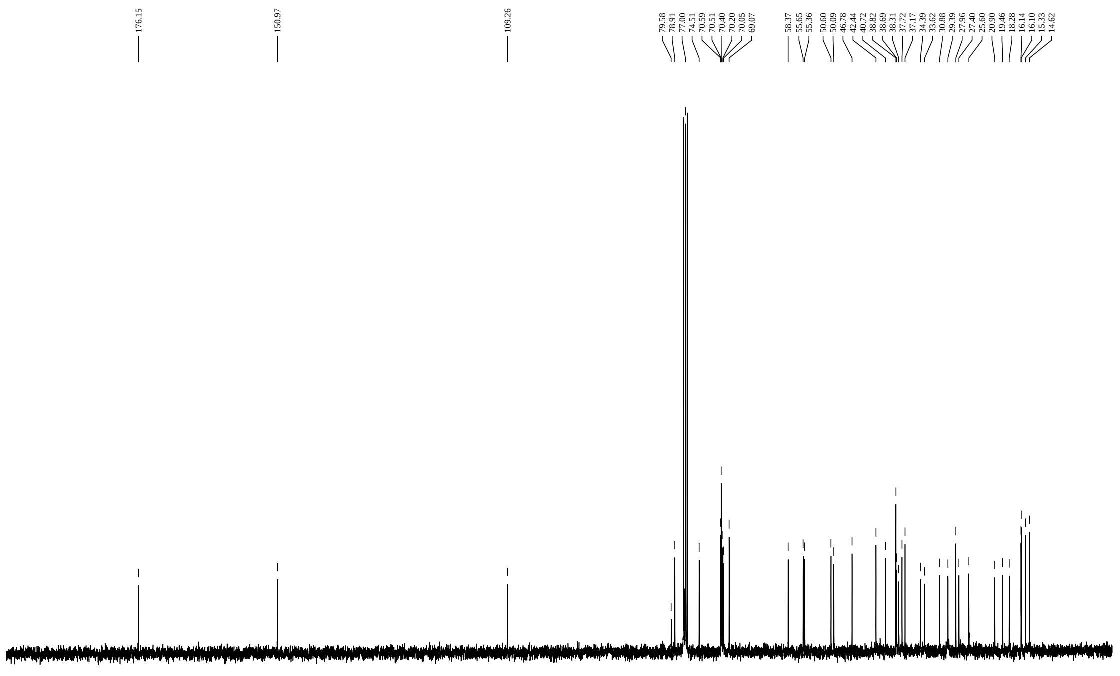

HRMS of compound **36**

Peking University Mass Spectrometry Sample Analysis Report

Analysis Info

Analysis Name	FTMS-19080136_Pos_20190826_000003.d	Acquisition Date	8/26/2019 1:25:39 PM
Sample	CYY-1-96	Instrument	Bruker Solarix XR FTMS
Comment		Operator	Peking University

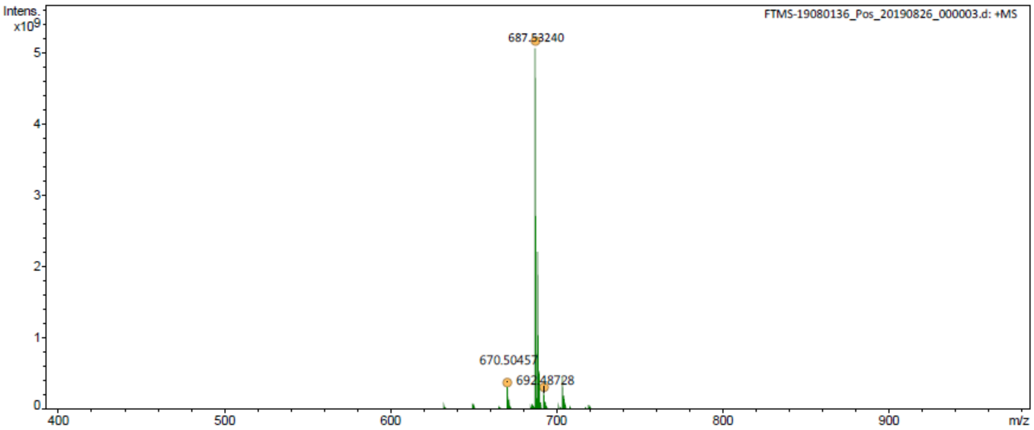

Meas. m/z	#	Ion Formula	Score	m/z	err [ppm]	Mean err [ppm]	mSigma	rdb	e⁻ Conf	N-Rule
670.504566	1	C41H68NO6	100.00	670.504115	-0.7	-0.0	15.5	9.0	even	ok
687.532405	1	C41H71N2O6	100.00	687.530664	-2.5	-1.5	13.5	8.0	even	ok
692.487277	1	C41H67NNaO6	100.00	692.486060	-1.8	-6.2	23.4	9.0	even	ok

^{1}H NMR of compound **37**

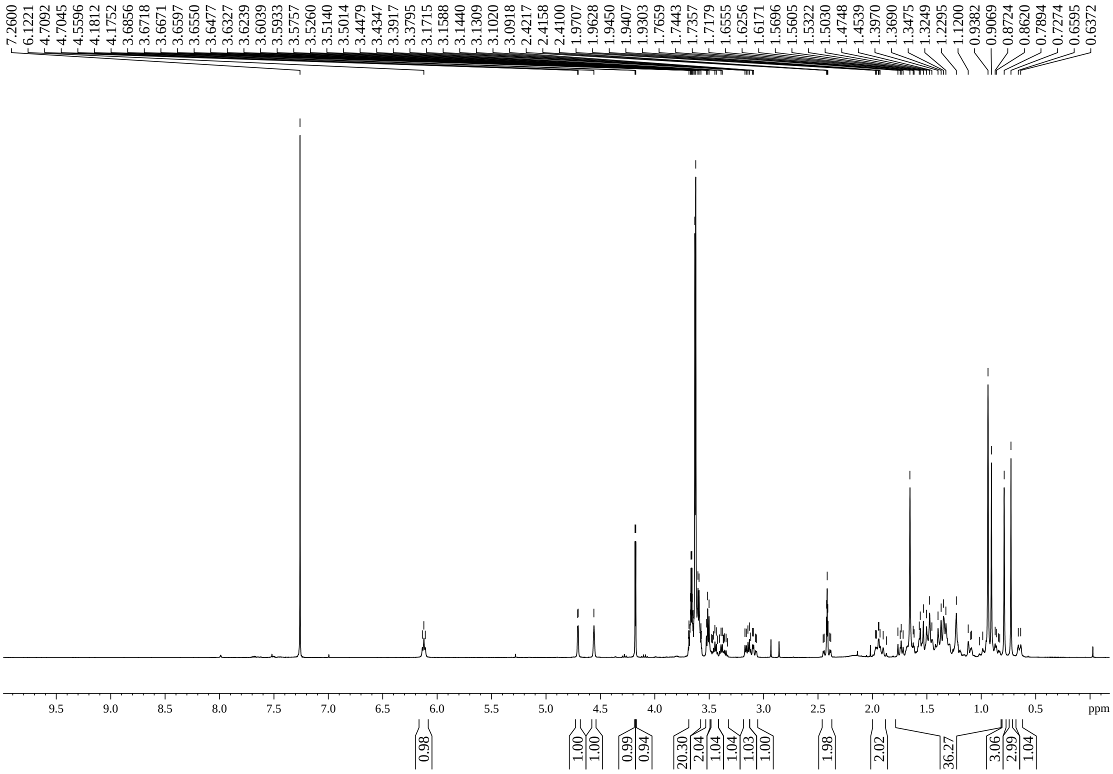

^{13}C NMR of compound **37**

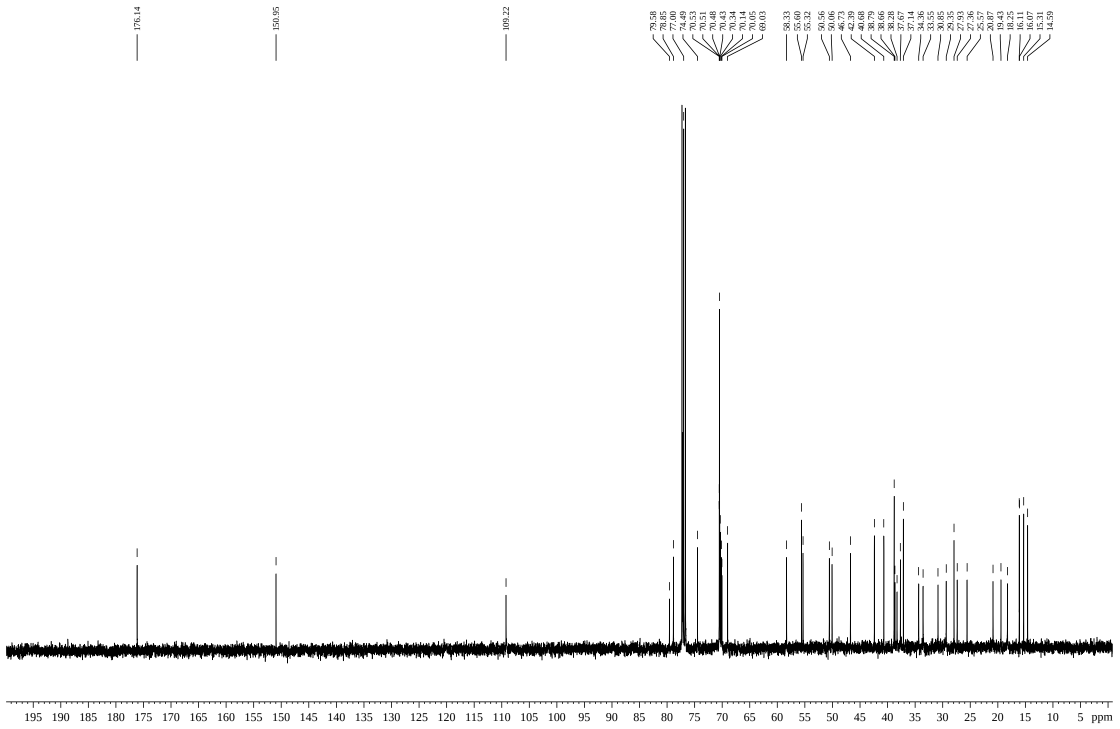

HRMS of compound **37**

Peking University Mass Spectrometry Sample Analysis Report

Analysis Info

Analysis Name	FTMS-18100355_Pos_20181103_000003.d	Acquisition Date	11/3/2018 8:10:39 PM
Sample	CYY2-92	Instrument	Bruker Solarix XR FTMS
Comment		Operator	Peking University

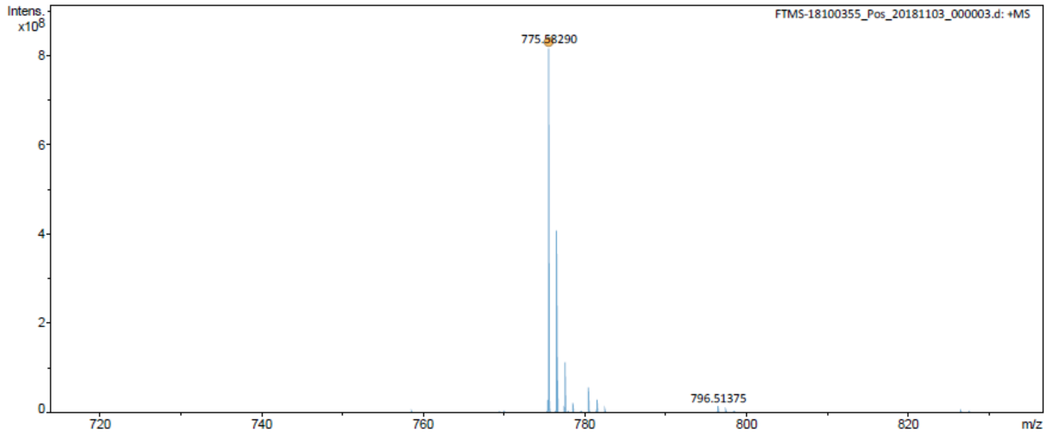

Meas. m/z	#	Ion Formula	Score	m/z	err [ppm]	Mean err [ppm]	mSigma	rdb	e⁻ Conf	N-Rule
775.582904	1	C45H79N2O8	100.00	775.583094	0.2	-0.7	3.5	8.0	even	ok

^{1}H NMR of compound **38**

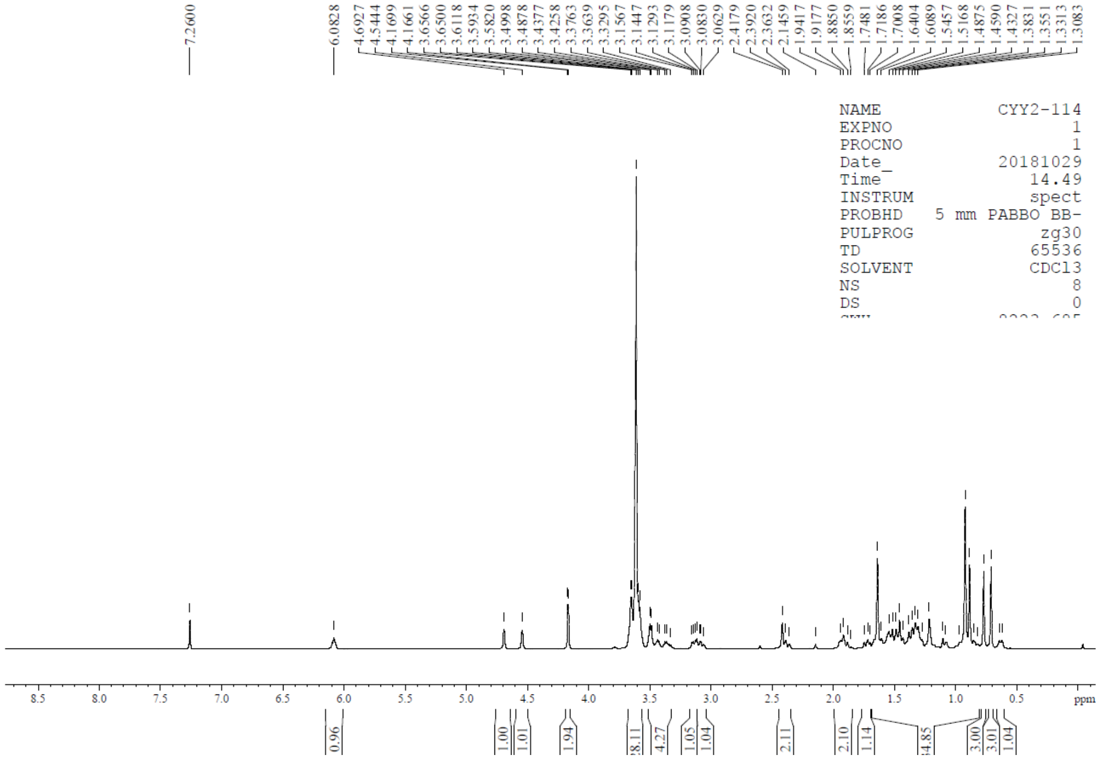

^{13}C NMR of compound **38**

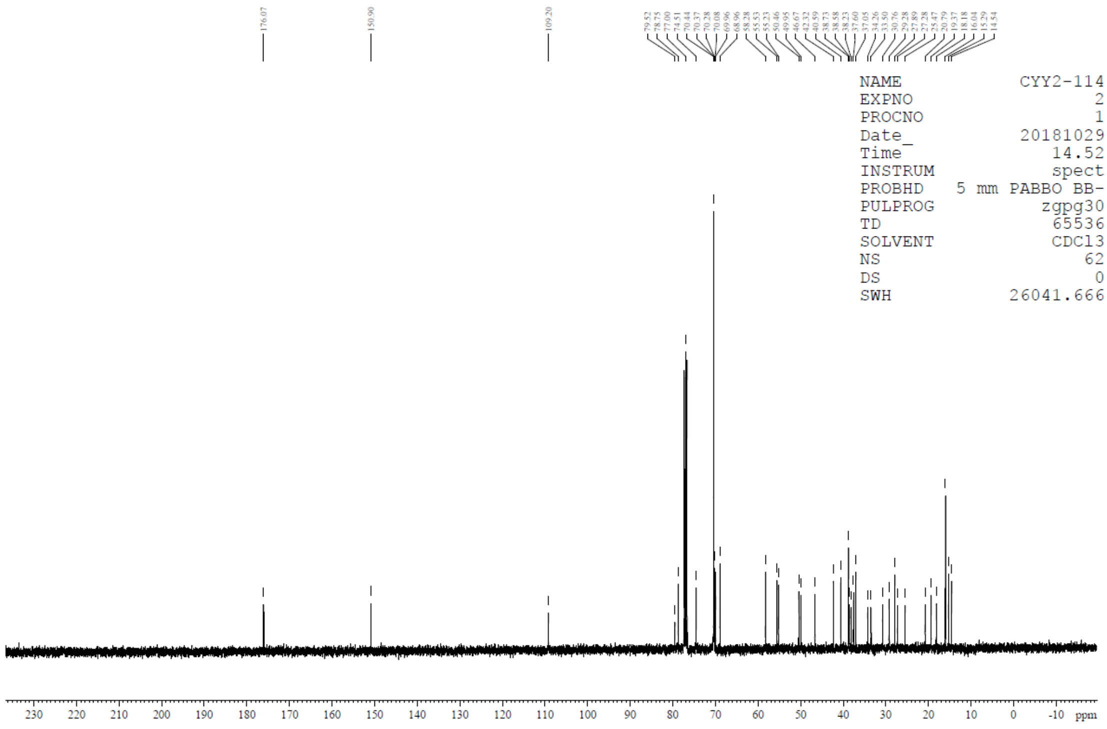

HRMS of compound **38**

Peking University Mass Spectrometry Sample Analysis Report

Analysis Info

Analysis Name	FTMS-18100355_Pos_20181103_000010.d	Acquisition Date	11/3/2018 8:23:56 PM
Sample	CYY2-114	Instrument	Bruker Solarix XR FTMS
Comment		Operator	Peking University

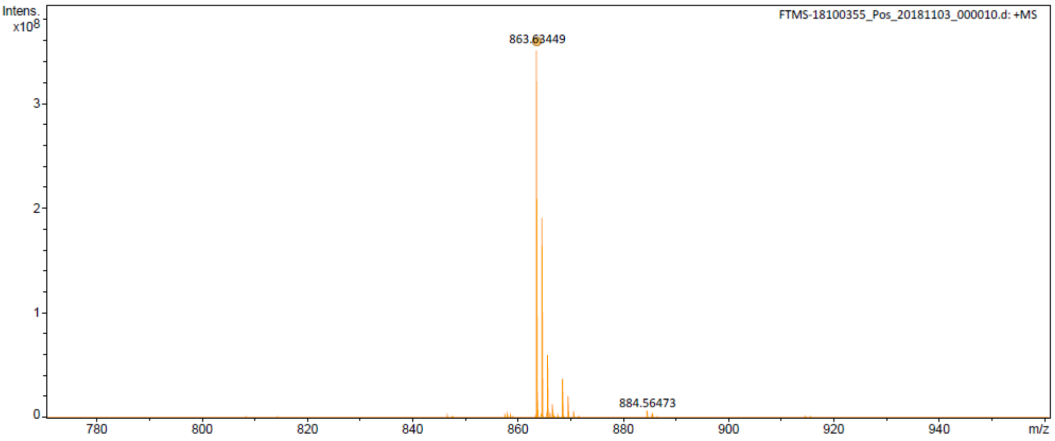

Meas. m/z	#	Ion Formula	Score	m/z	err [ppm]	Mean err [ppm]	mSigma	rdb	e⁻ Conf	N-Rule
863.634490	1	C49H87N2O10	100.00	863.635523	1.2	1.1	3.5	8.0	even	ok

^{1}H NMR of compound **51**

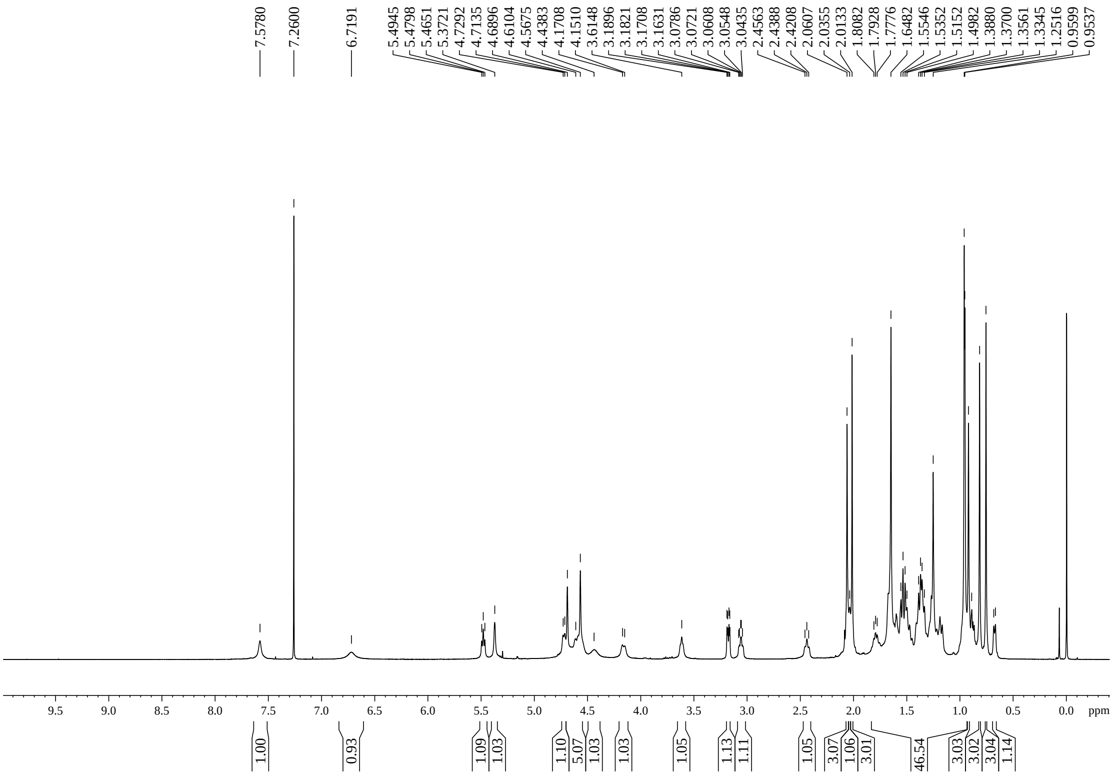

^{13}C NMR of compound **51**

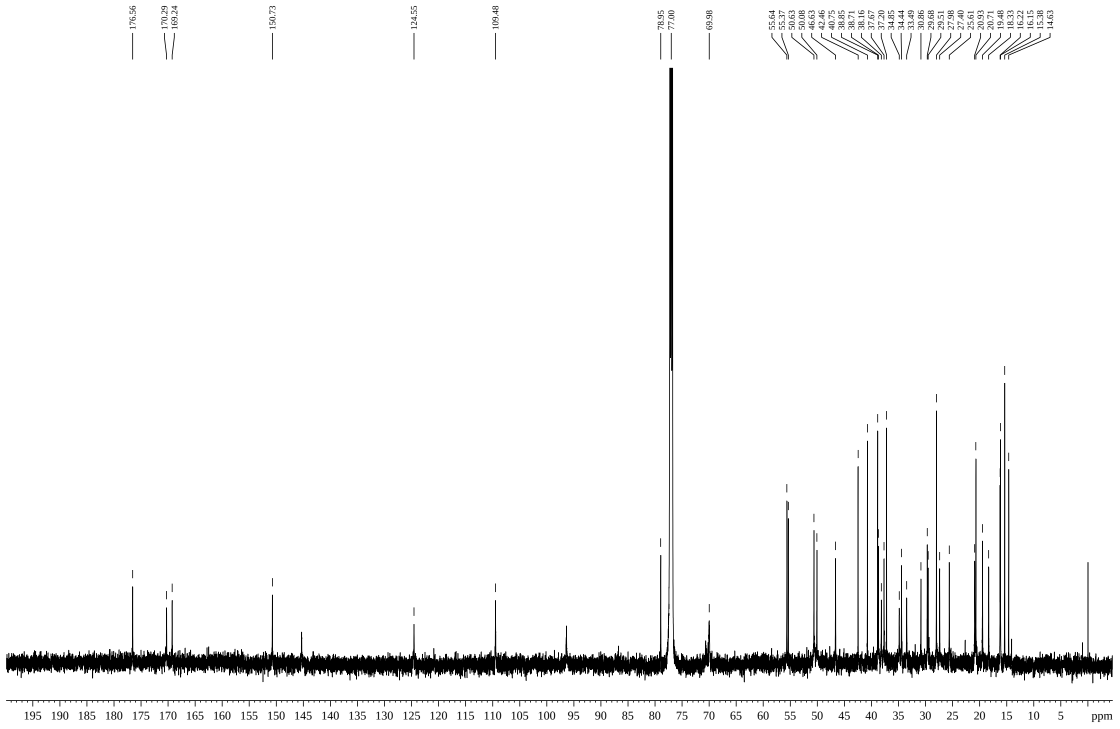

MALDI-TOF of compound **51**

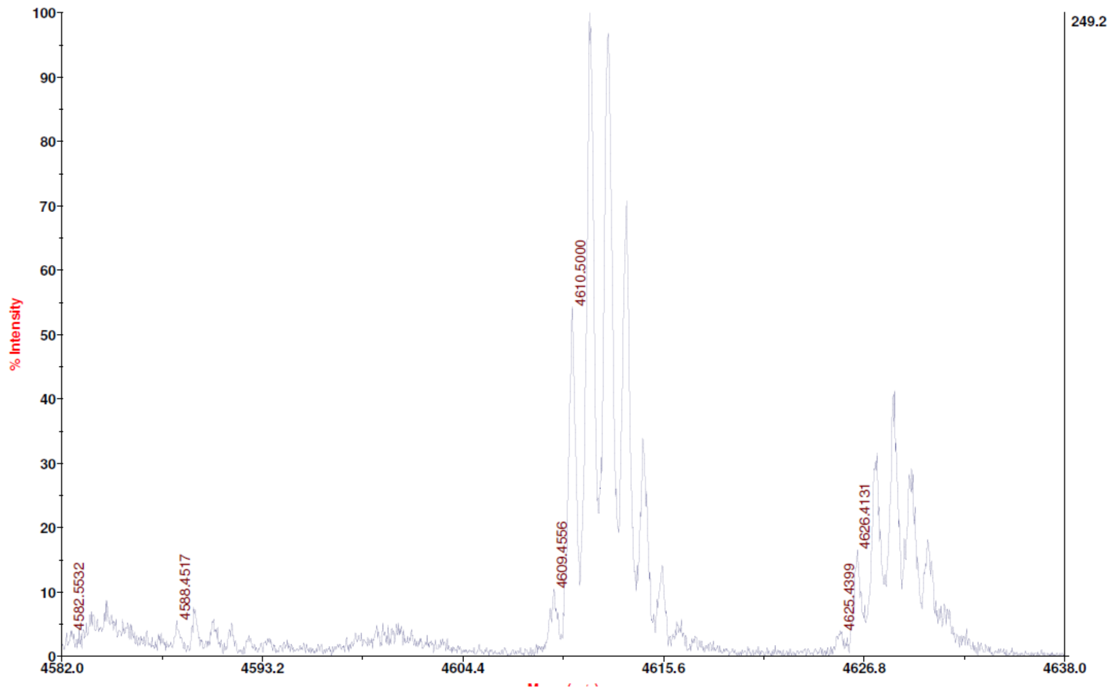

^{1}H NMR of compound **52**

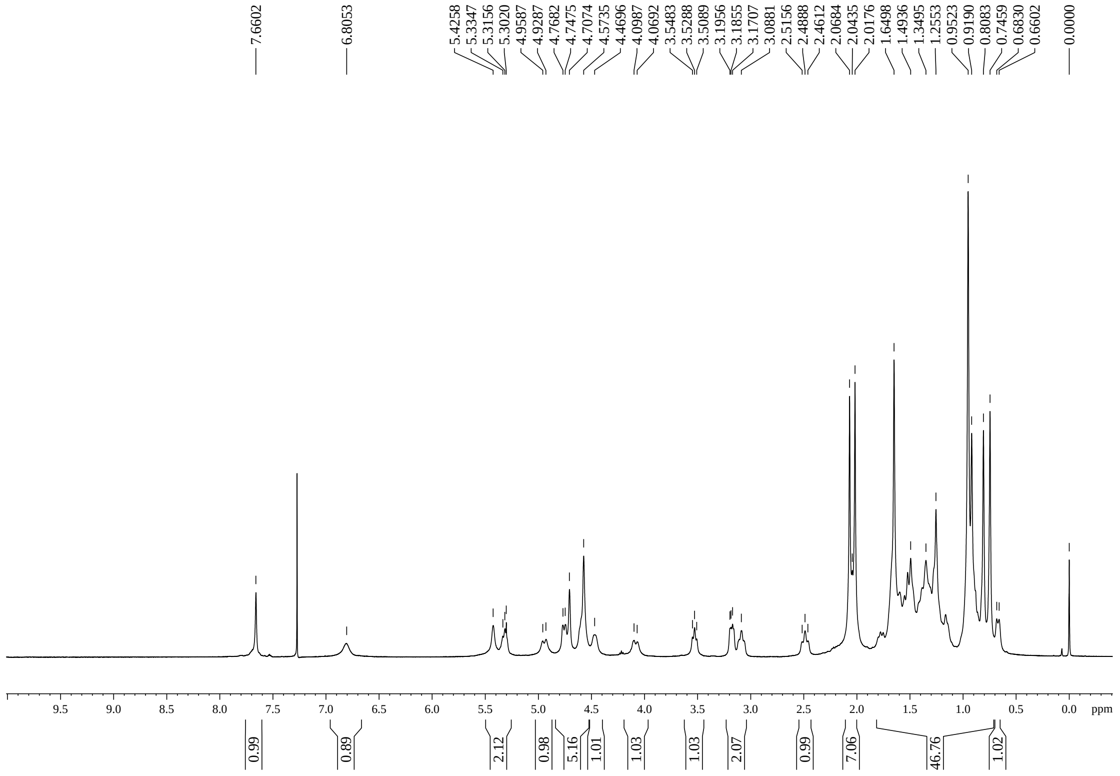

^{13}C NMR of compound **52**

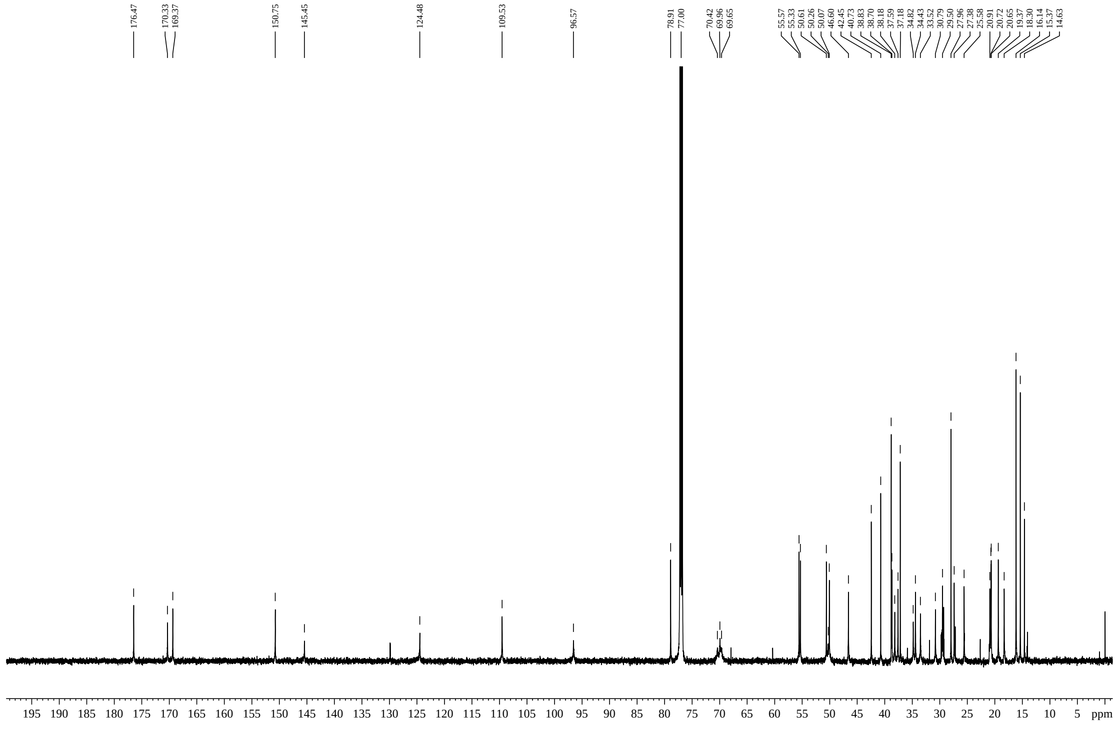

MALDI-TOF of compound **52**

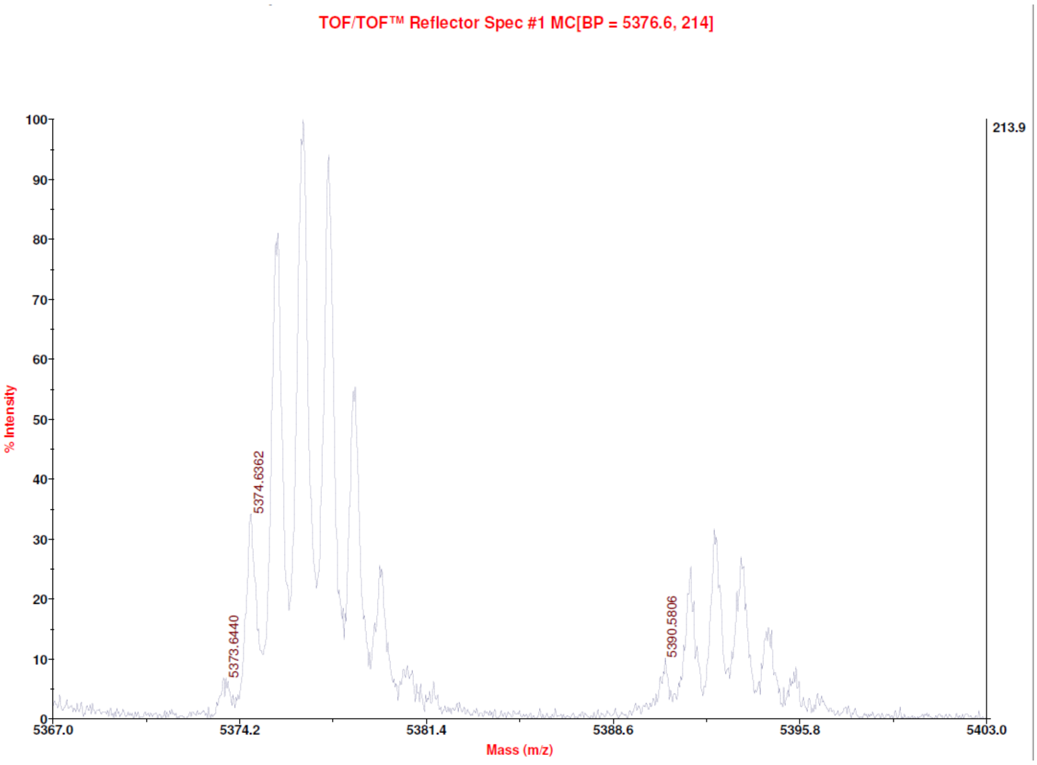

^{1}H NMR of compound **53**

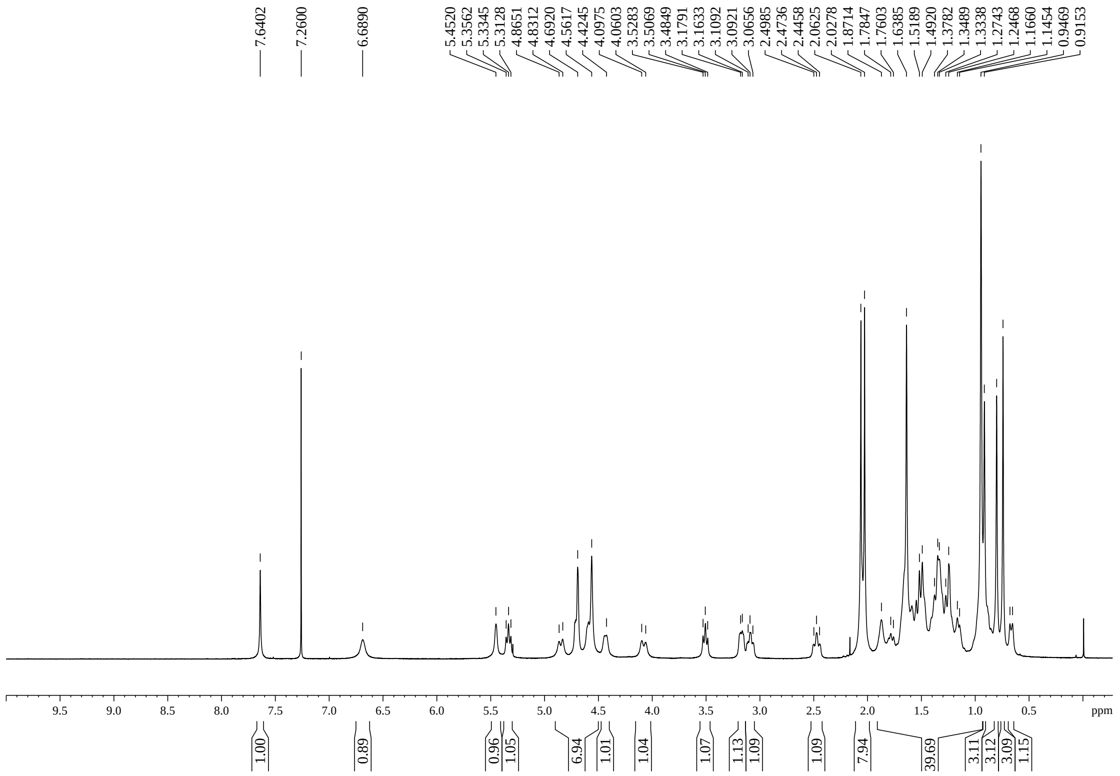

^{13}C NMR of compound **53**

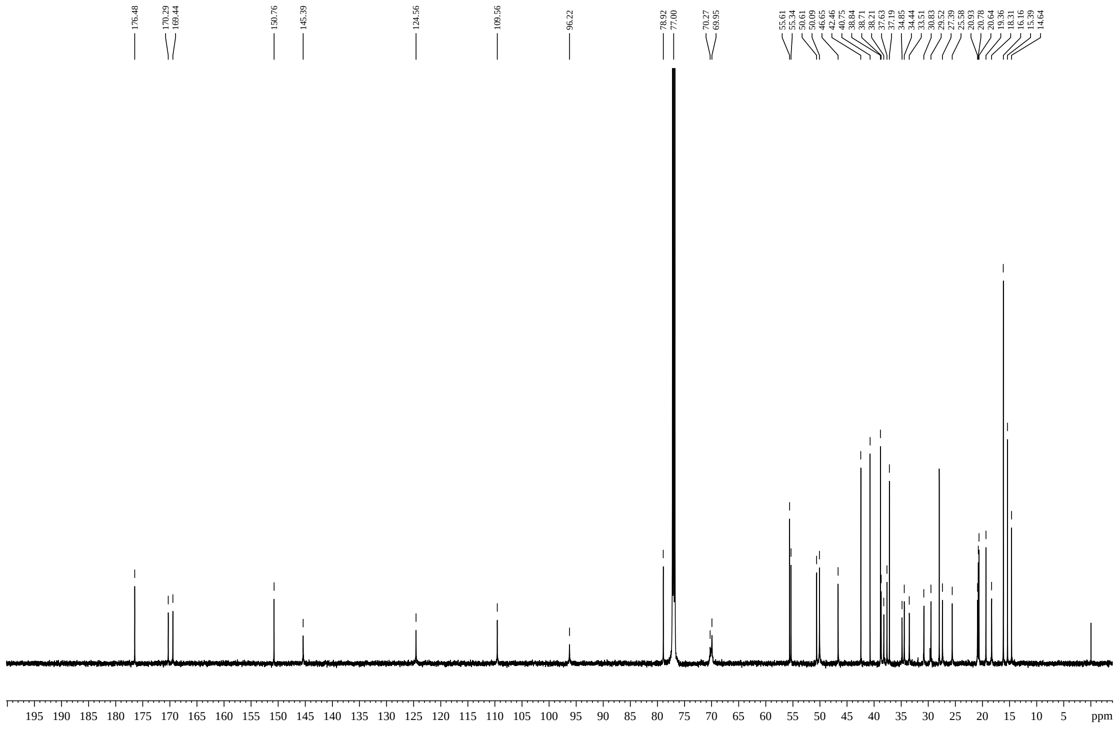

MALDI-TOF of compound **53**

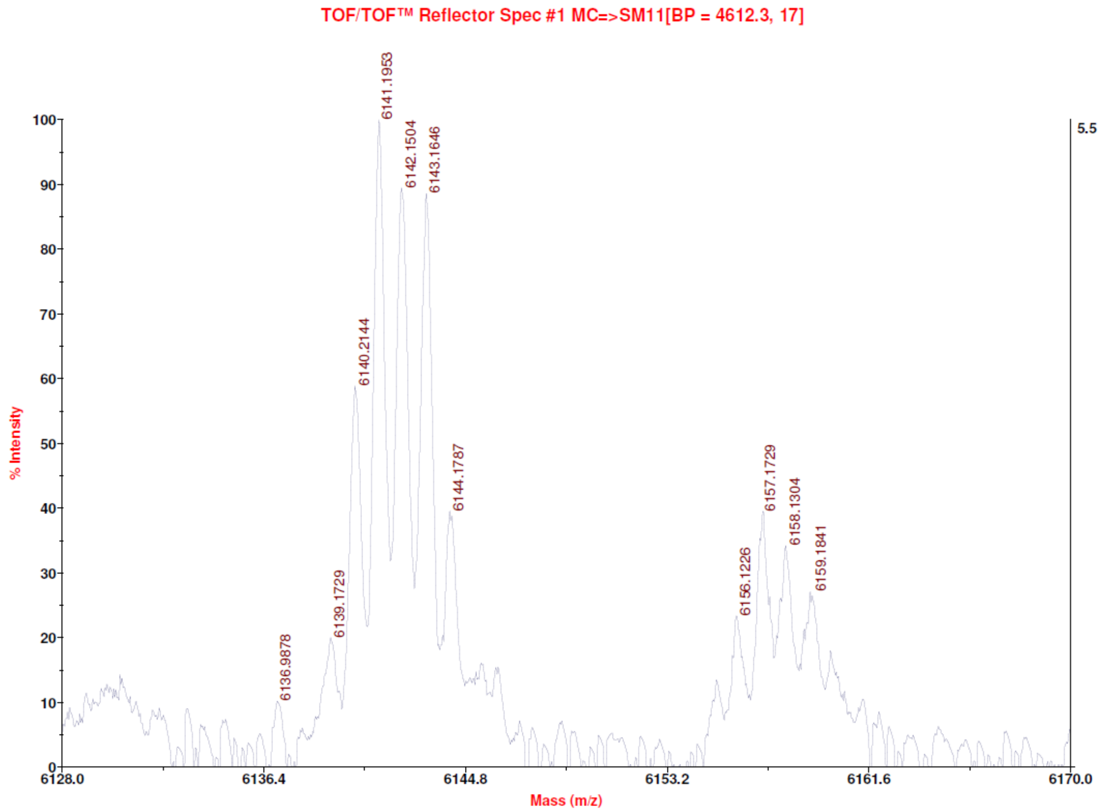

^{1}H NMR of compound **54**

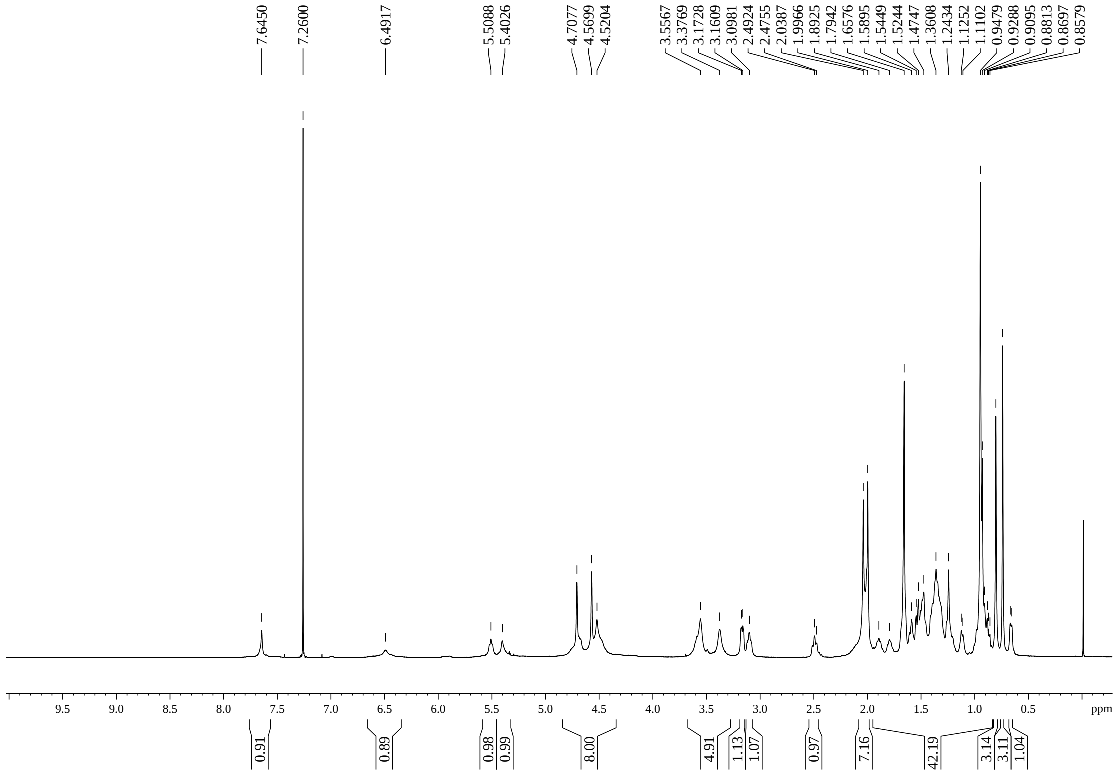

^{13}C NMR of compound **54**

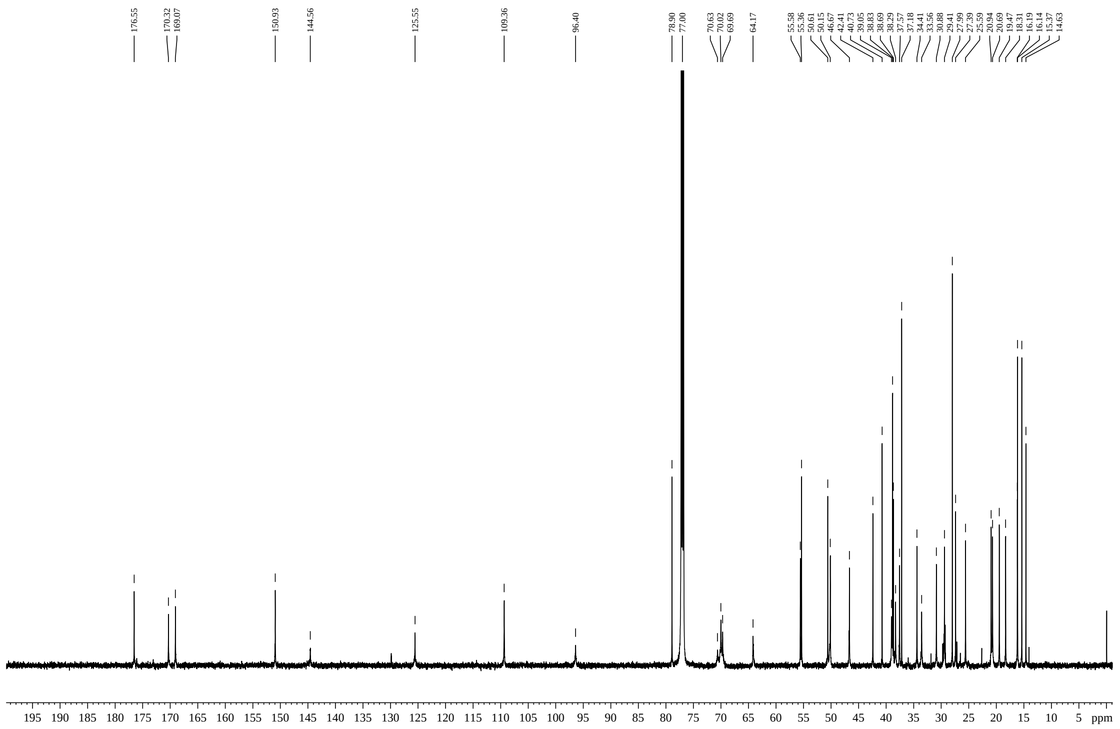

MALDI-TOF of compound **54**

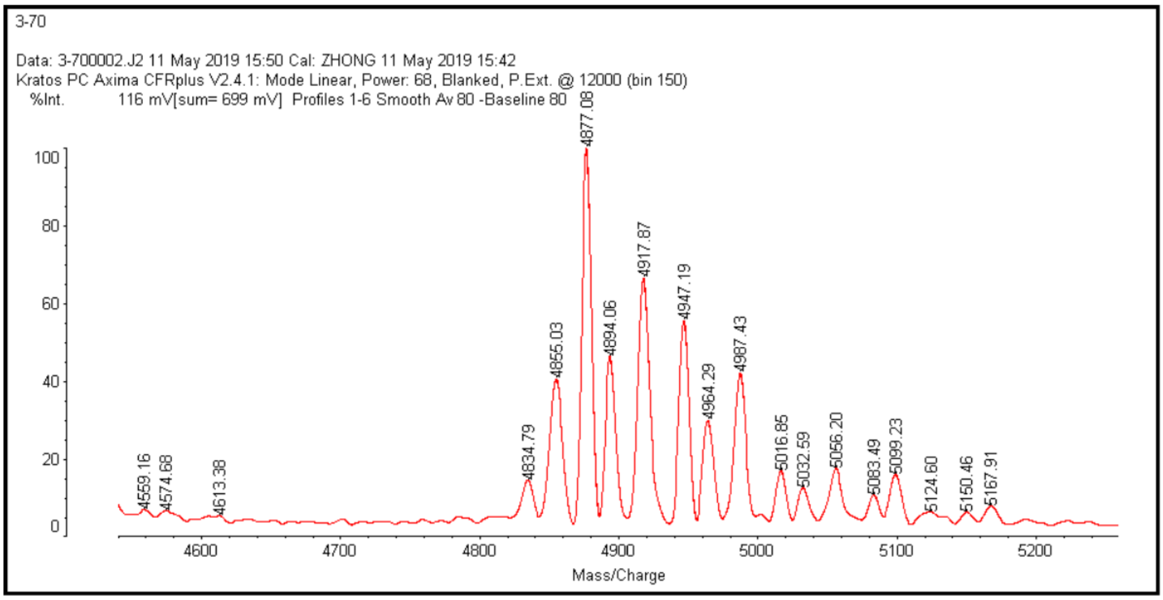

^{1}H NMR of compound **55**

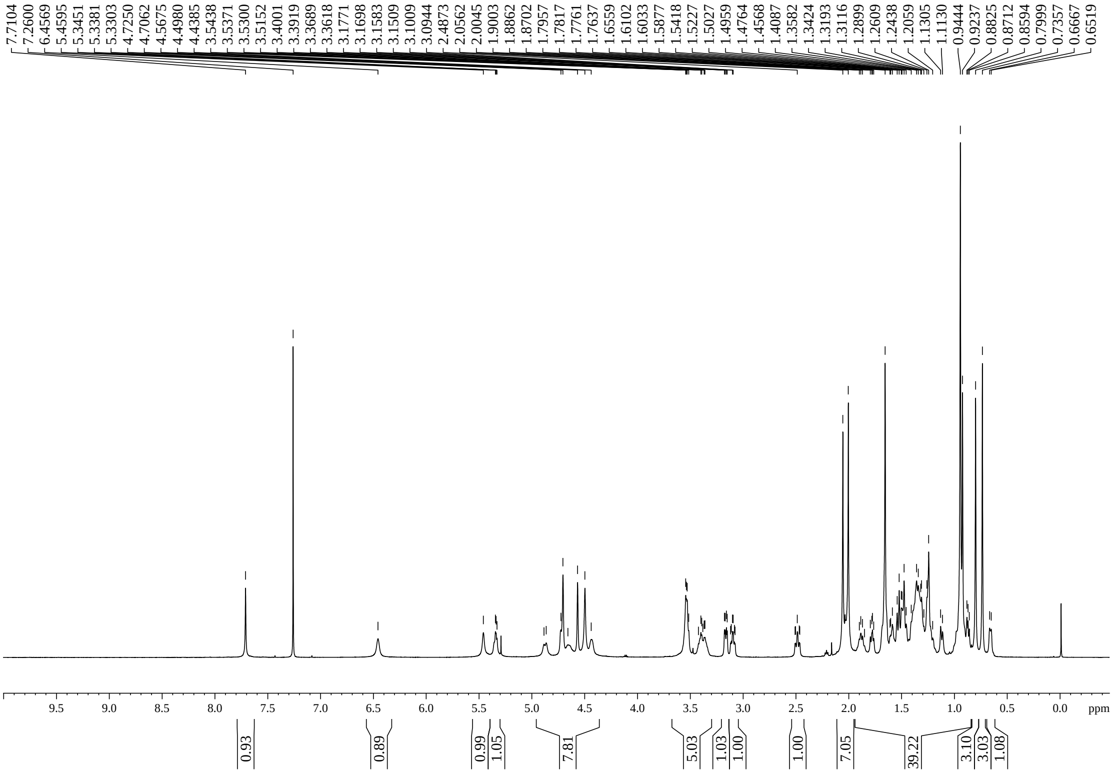

^{13}C NMR of compound **55**

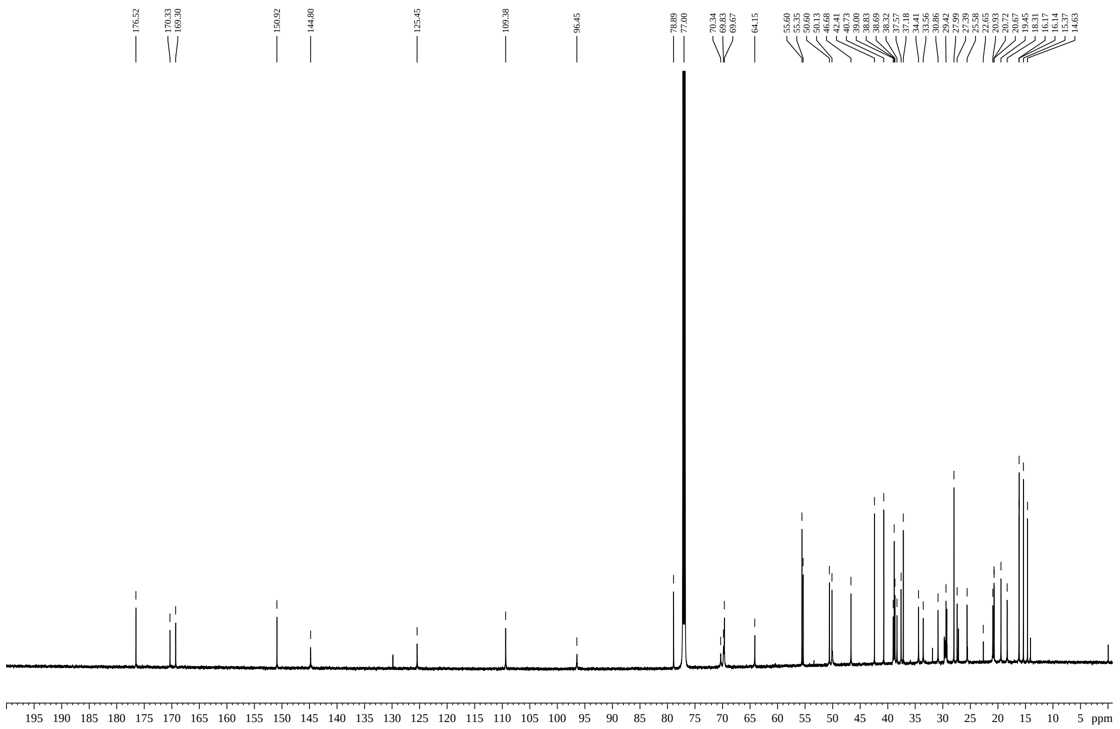

MALDI-TOF of compound **55**

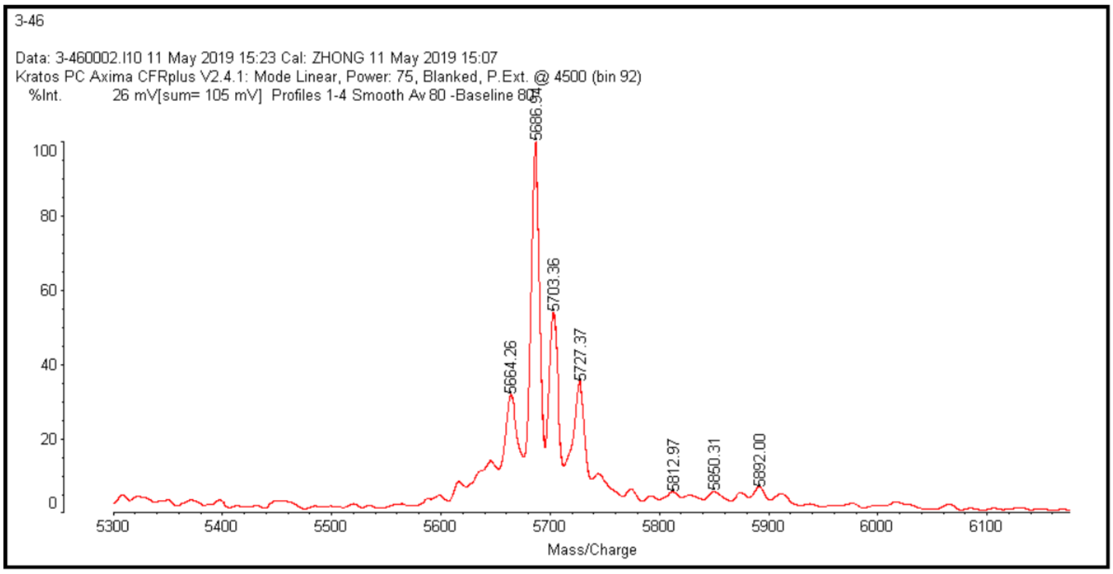

^{1}H NMR of compound **56**

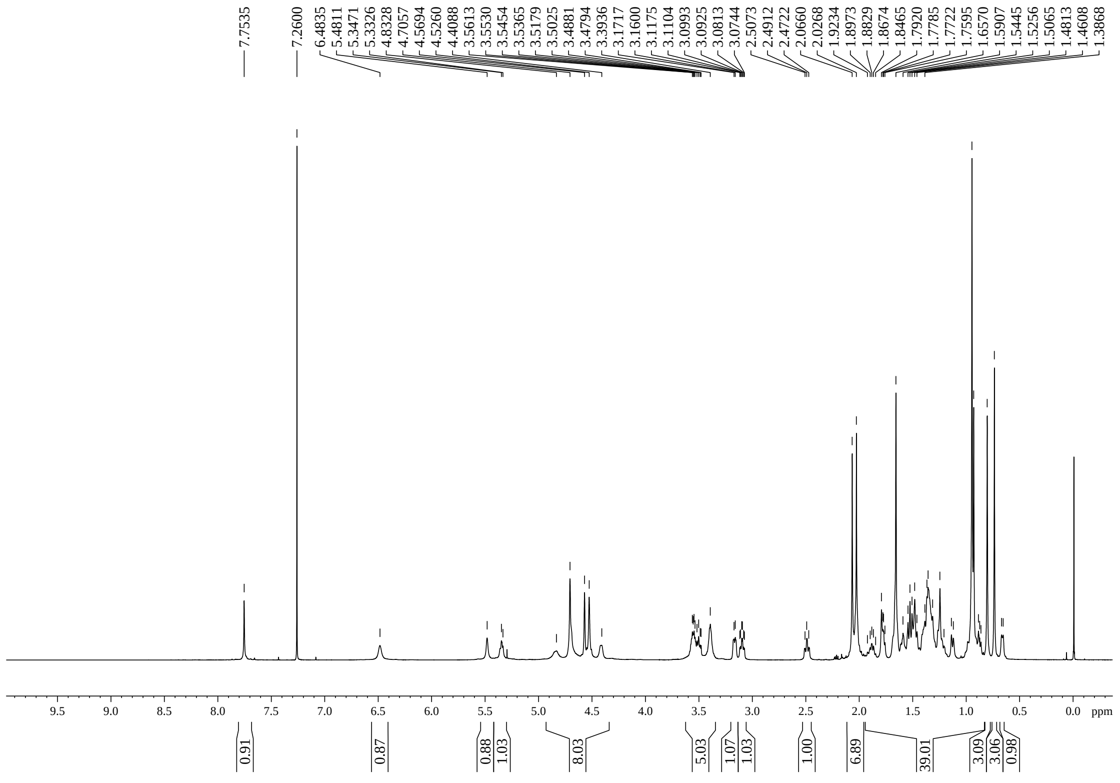

^{13}C NMR of compound **56**

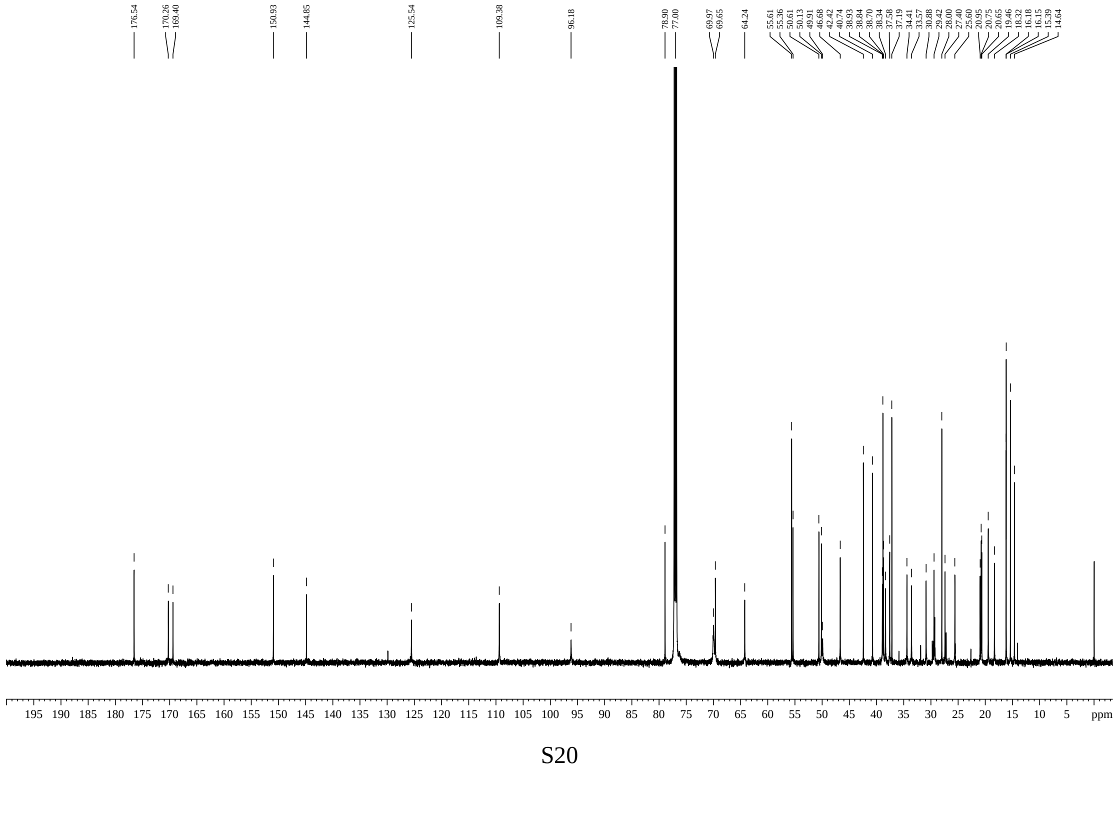

MALDI-TOF of compound **56**

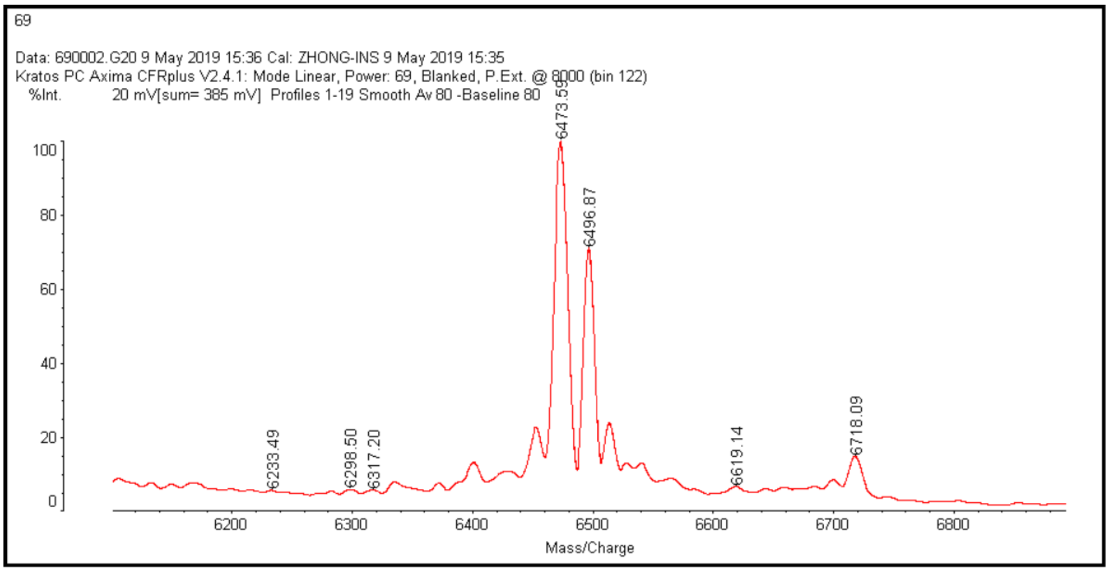

^{1}H NMR of compound **57**

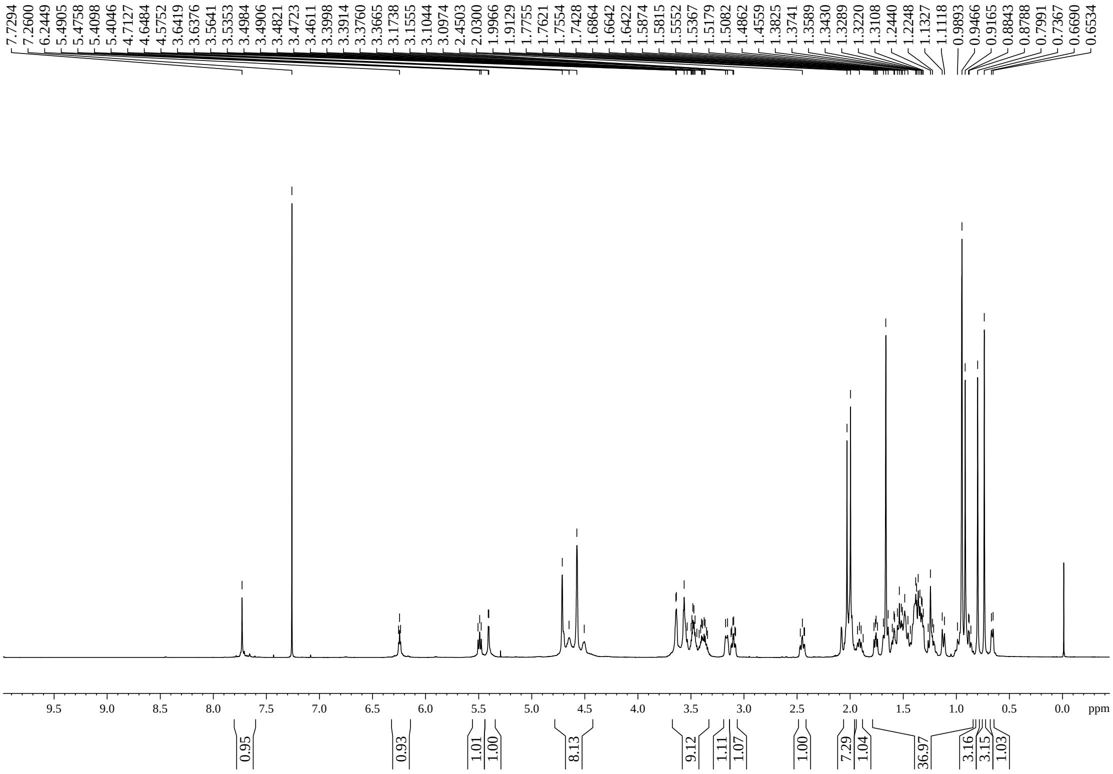

^{13}C NMR of compound **57**

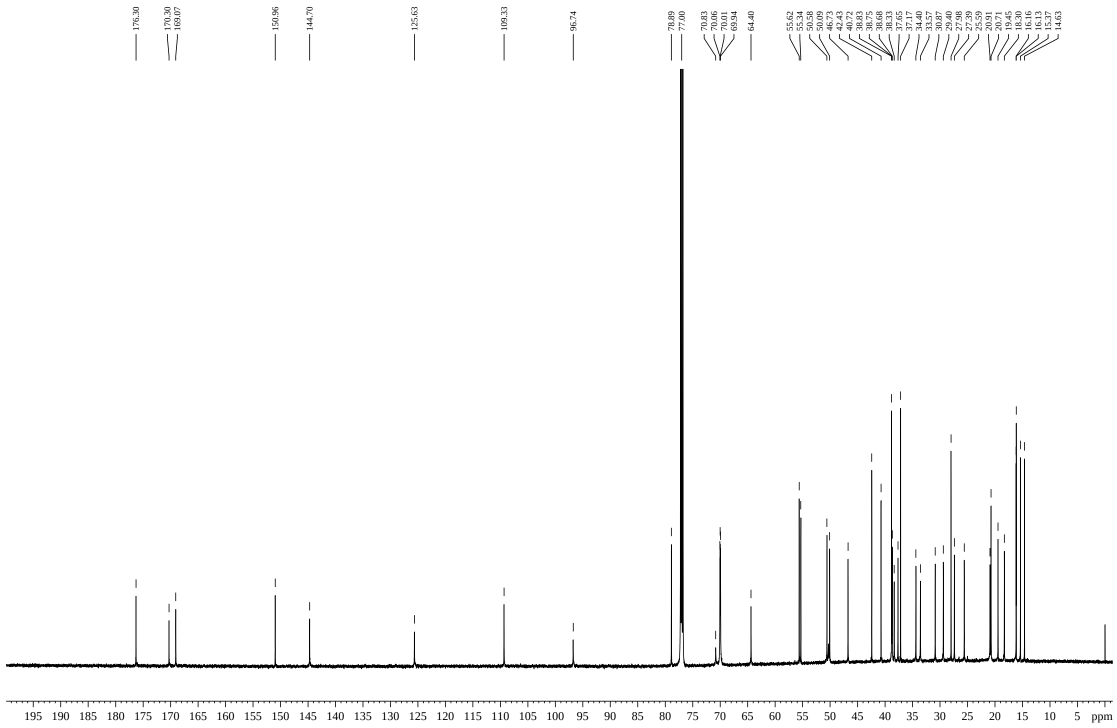

MALDI-TOF of compound **57**

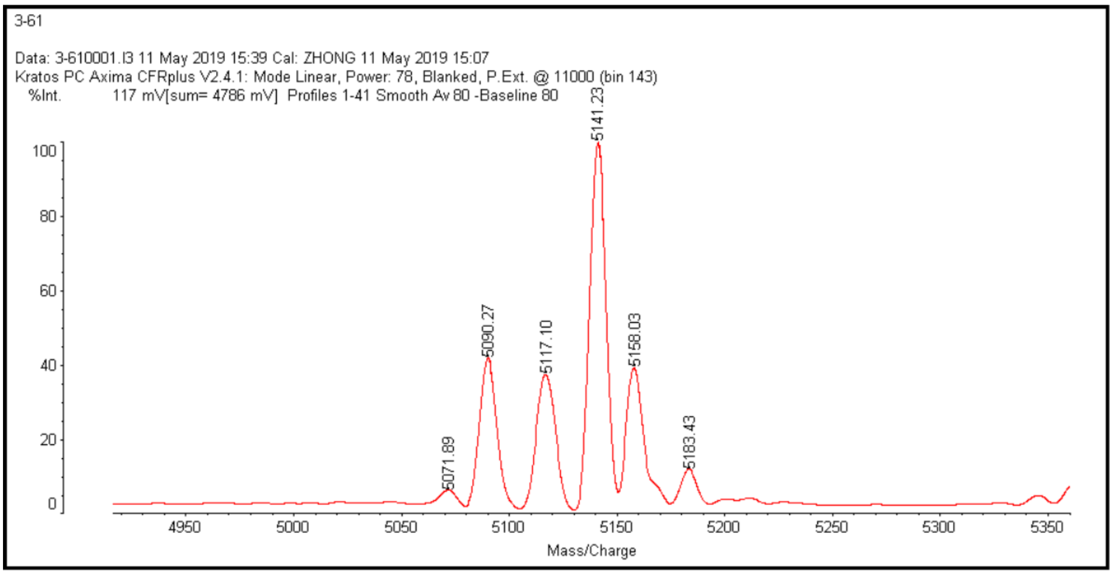

^{1}H NMR of compound **58**

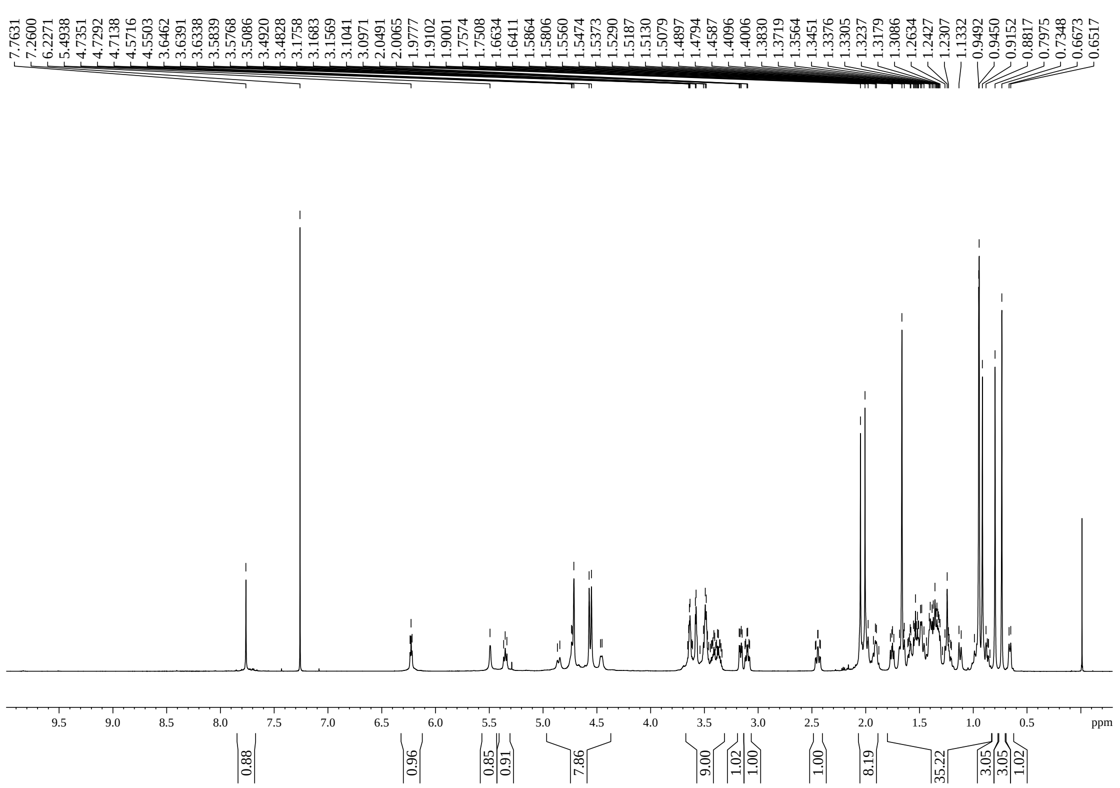

^{13}C NMR of compound **58**

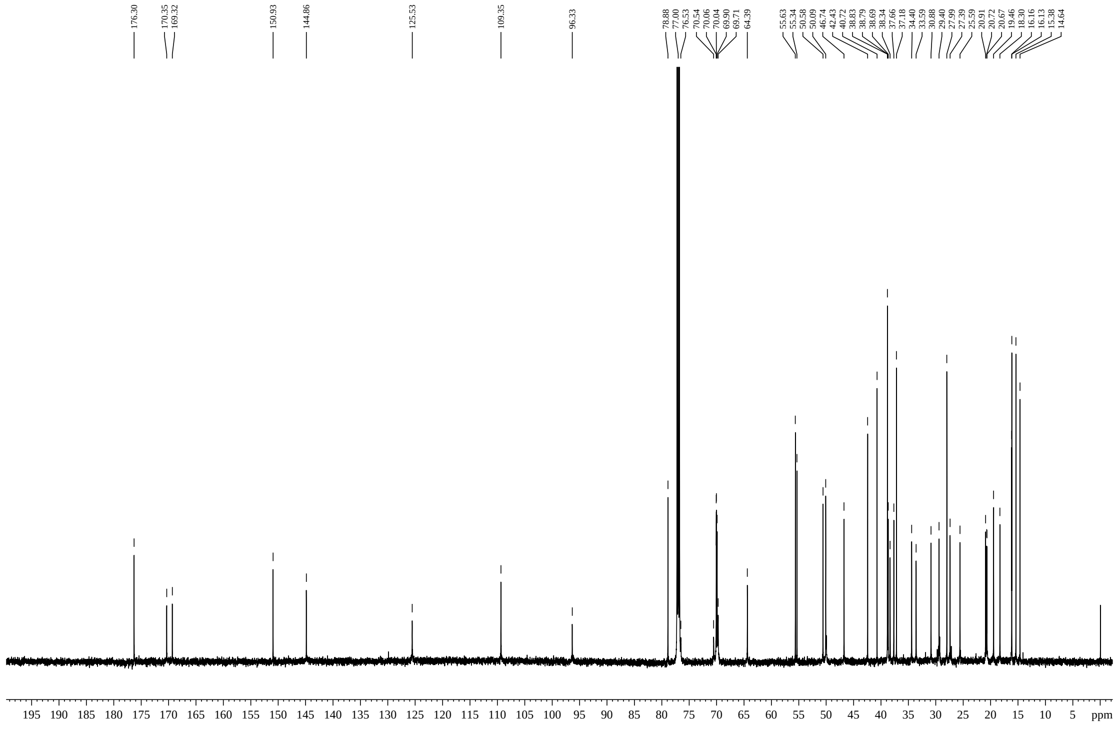

MALDI-TOF of compound **58**

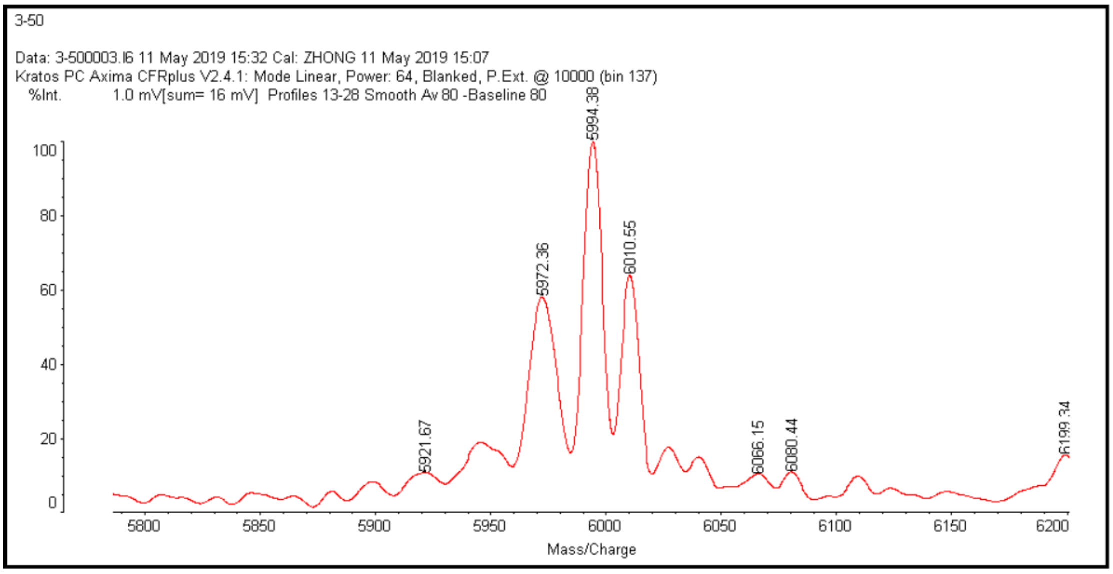

^{1}H NMR of compound **59**

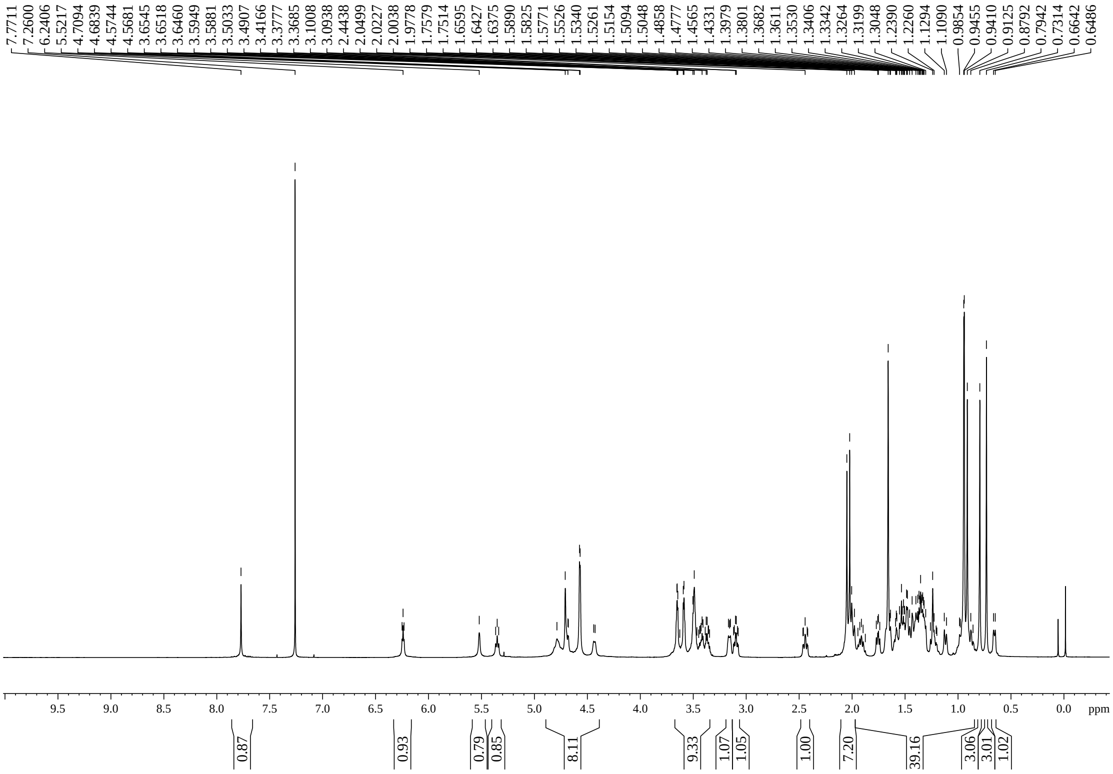

^{13}C NMR of compound **59**

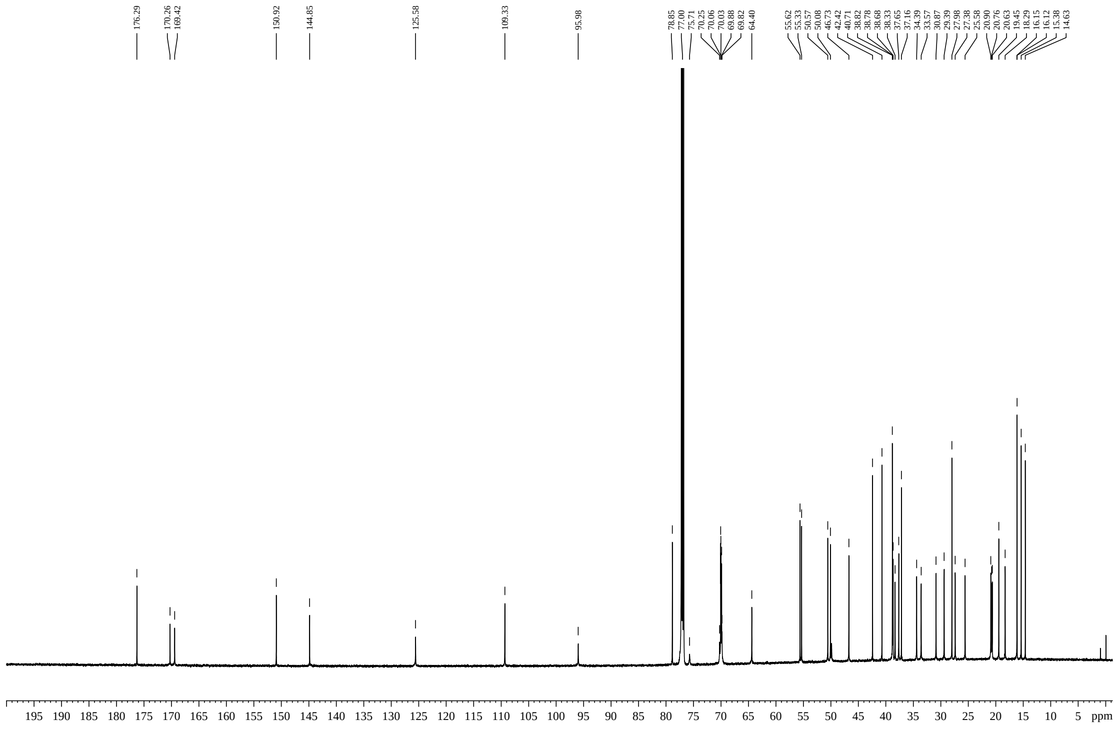

MALDI-TOF of compound **59**

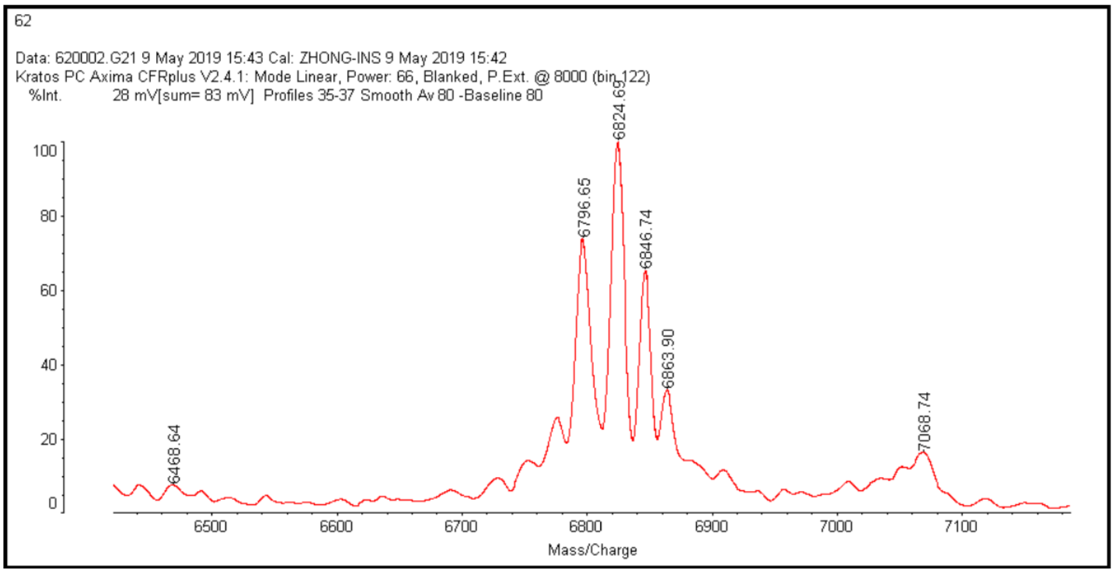

^{1}H NMR of compound **60**

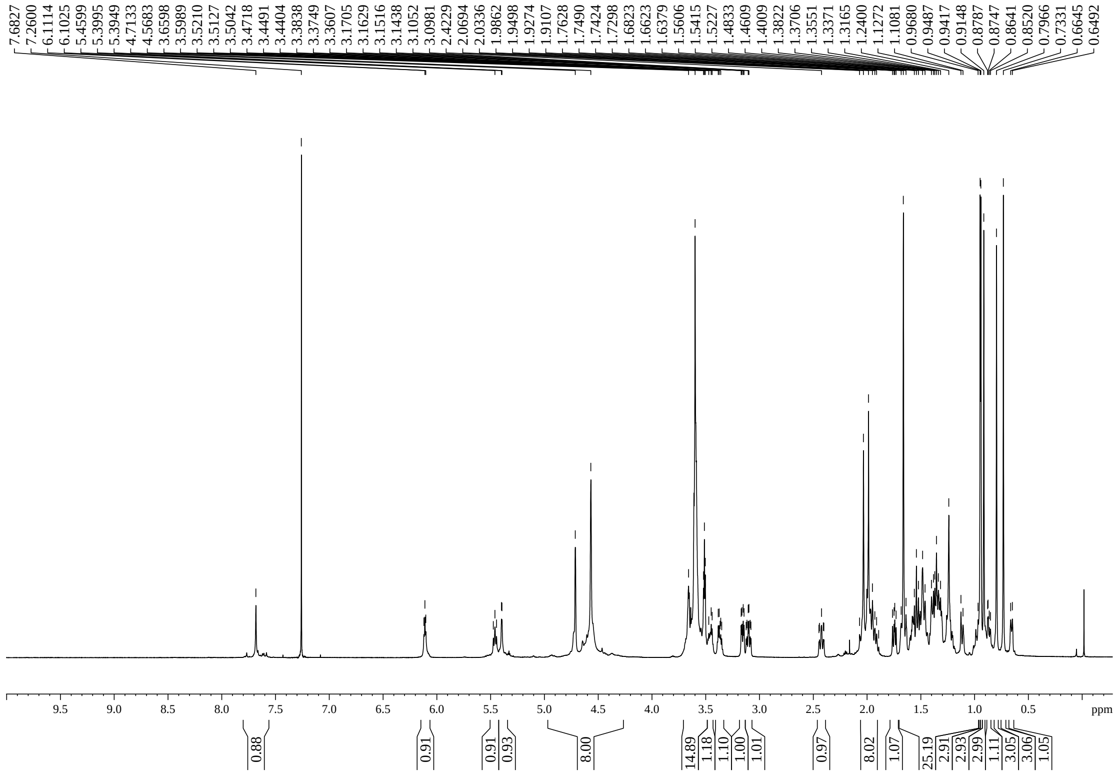

^{13}C NMR of compound **60**

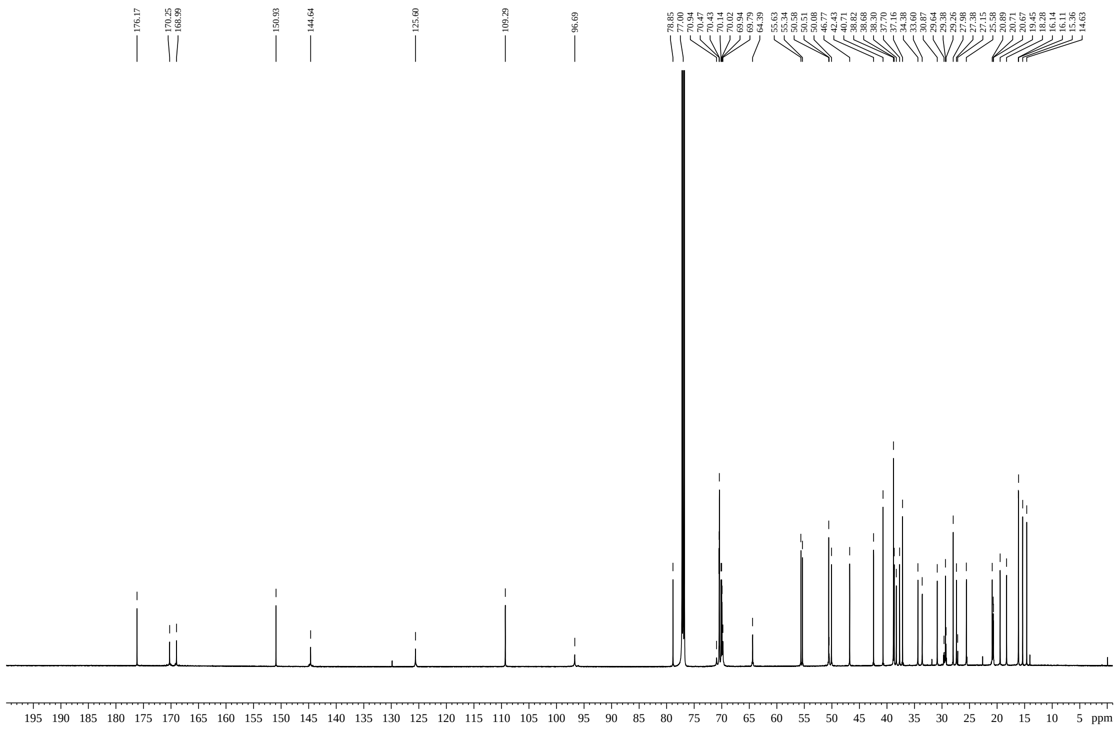

MALDI-TOF of compound **60**

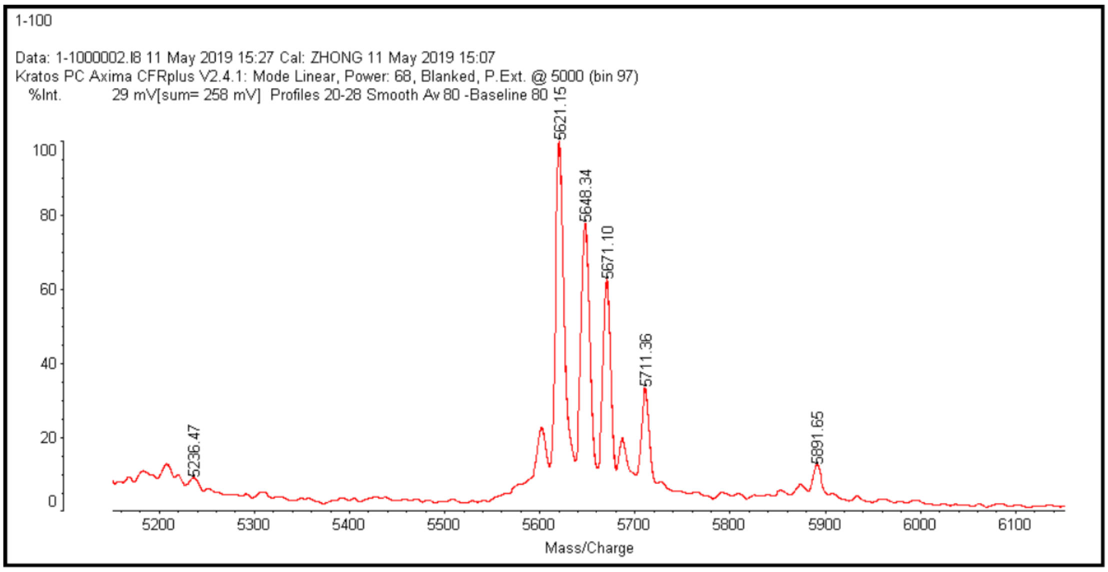

^{1}H NMR of compound **61**

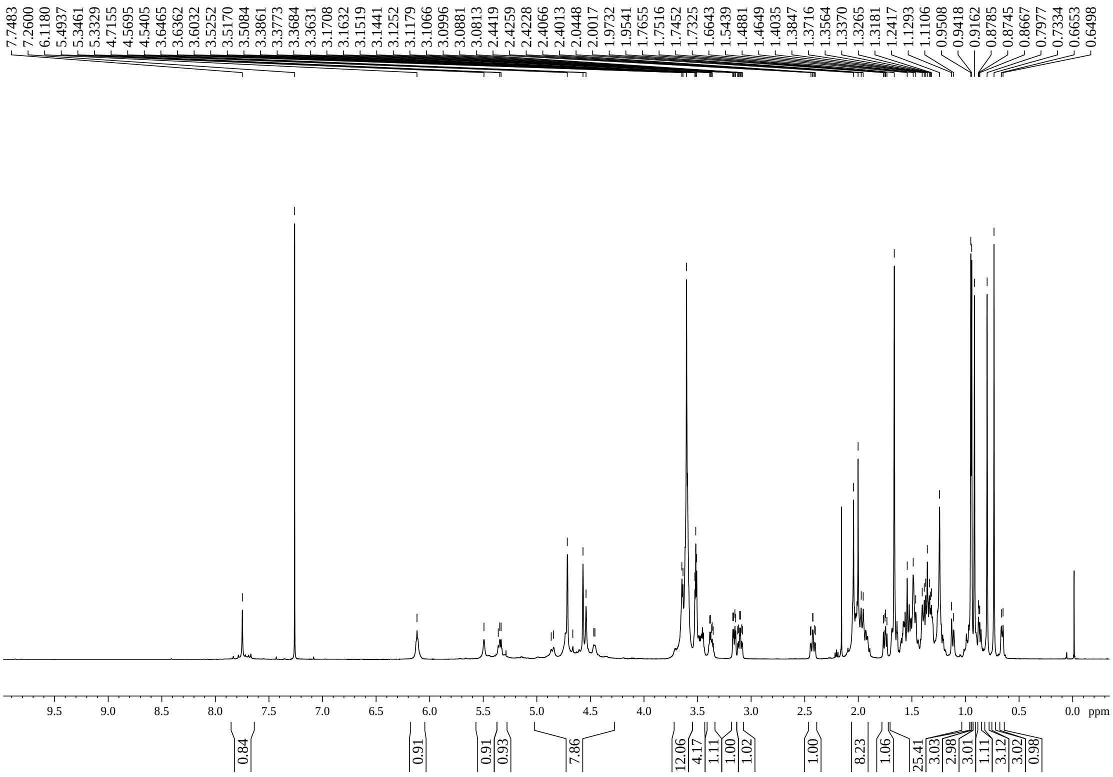

^{13}C NMR of compound **61**

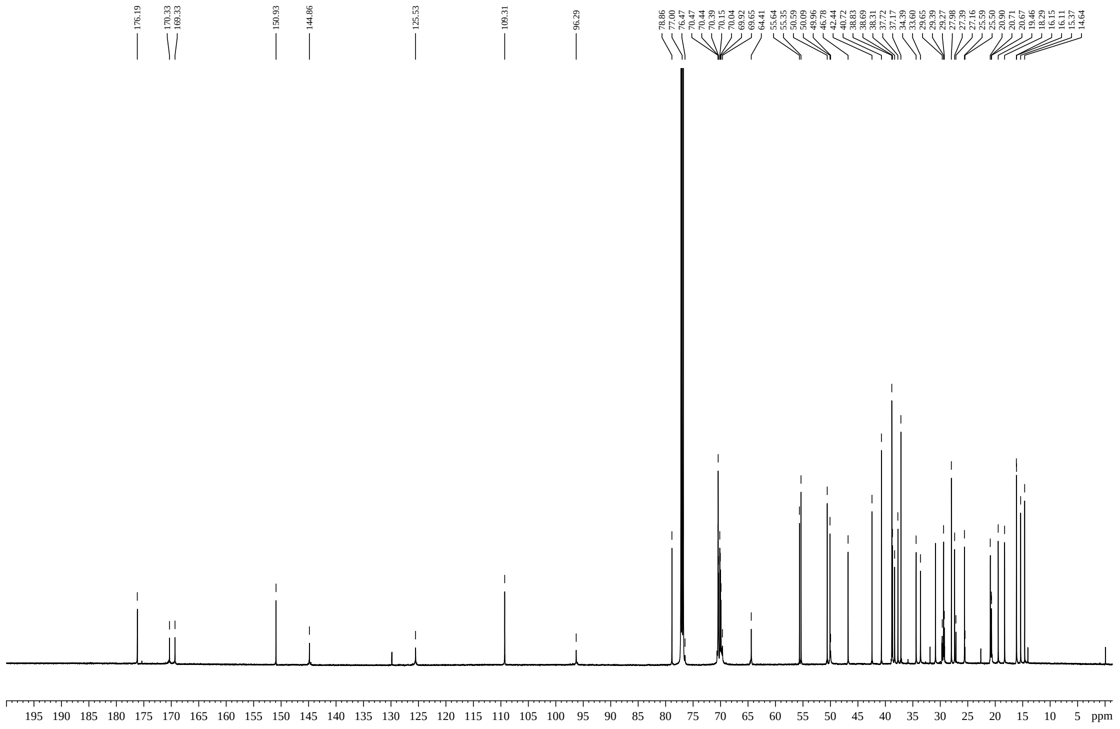

MALDI-TOF of compound **61**

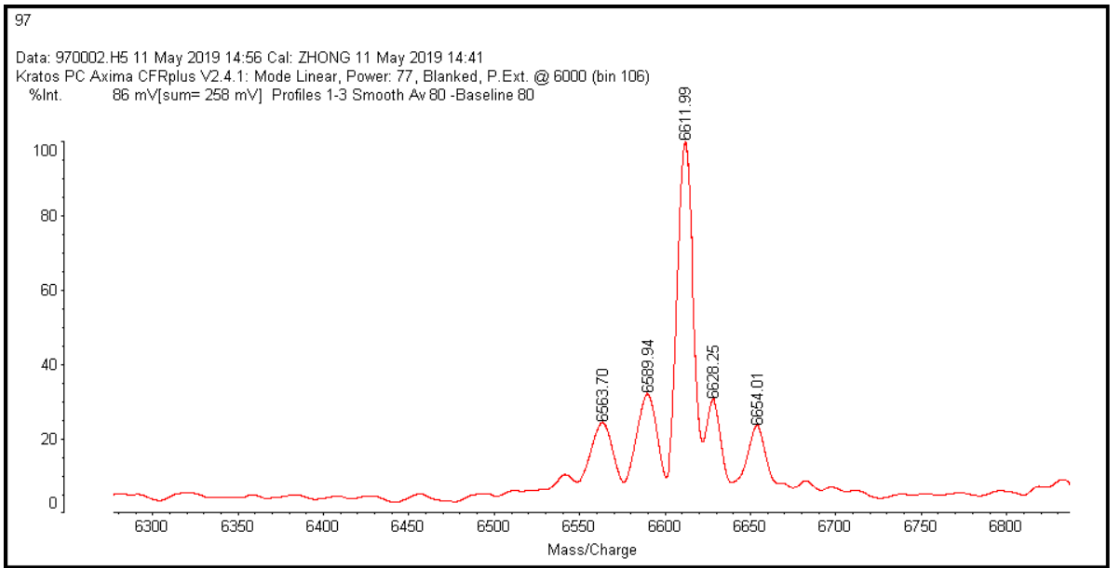

^{1}H NMR of compound **62**

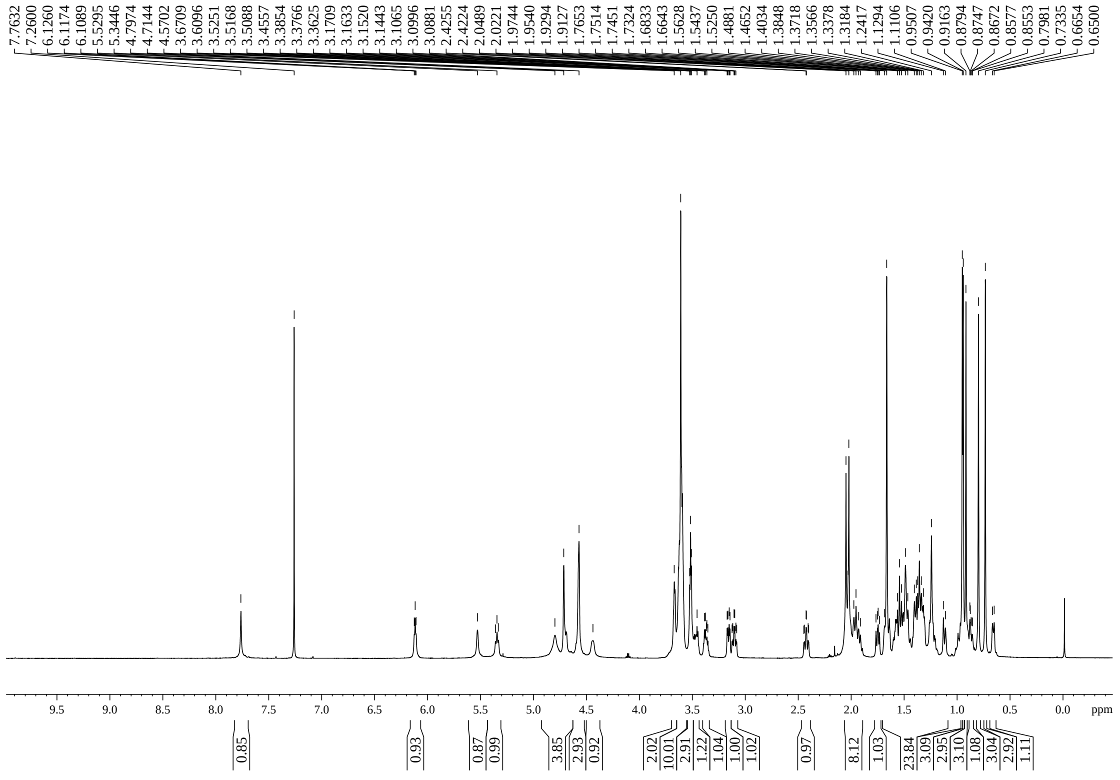

^{13}C NMR of compound **62**

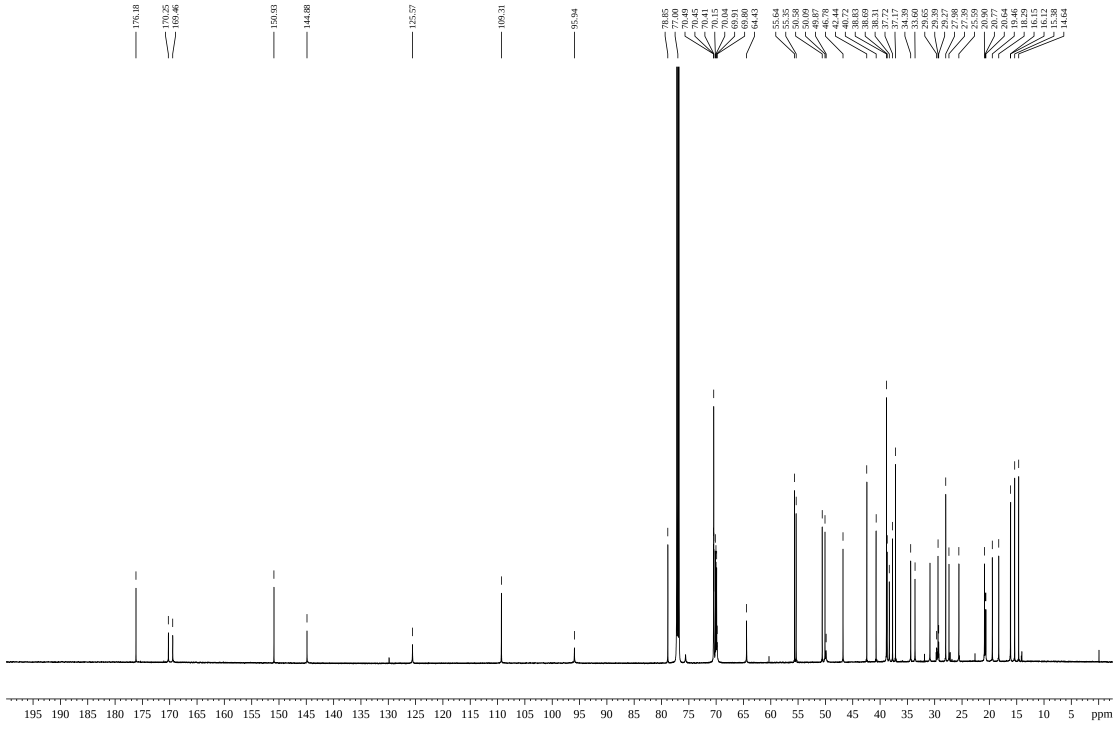

MALDI-TOF of compound **62**

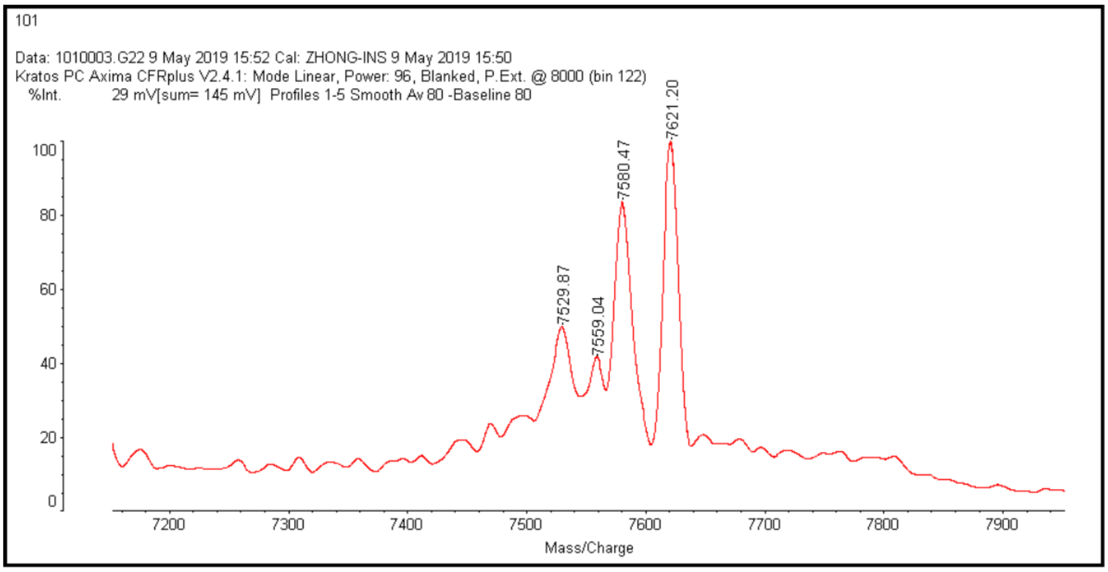

^{1}H NMR of compound **63**

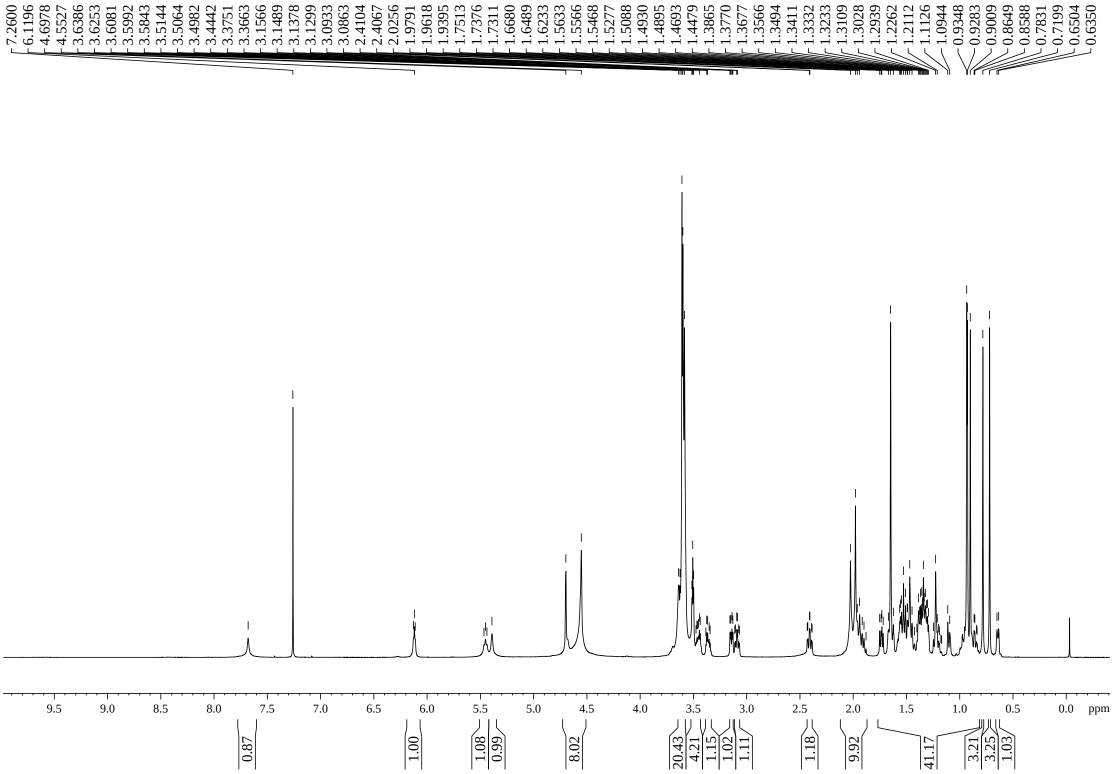

^{13}C NMR of compound **63**

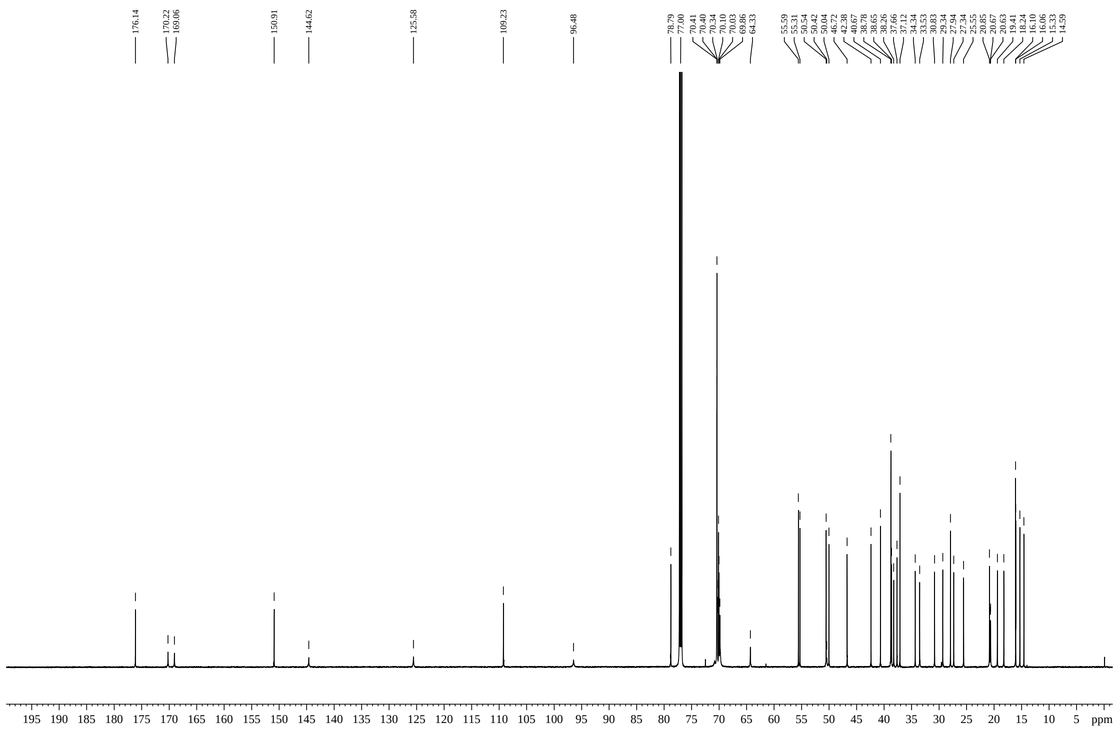

MALDI-TOF of compound **63**

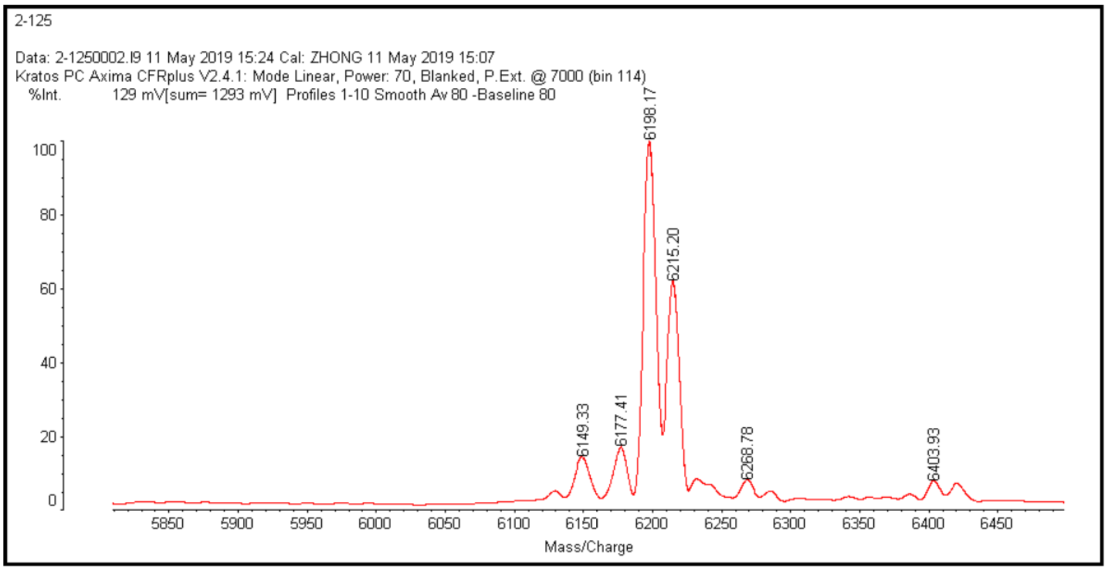

^{1}H NMR of compound **64**

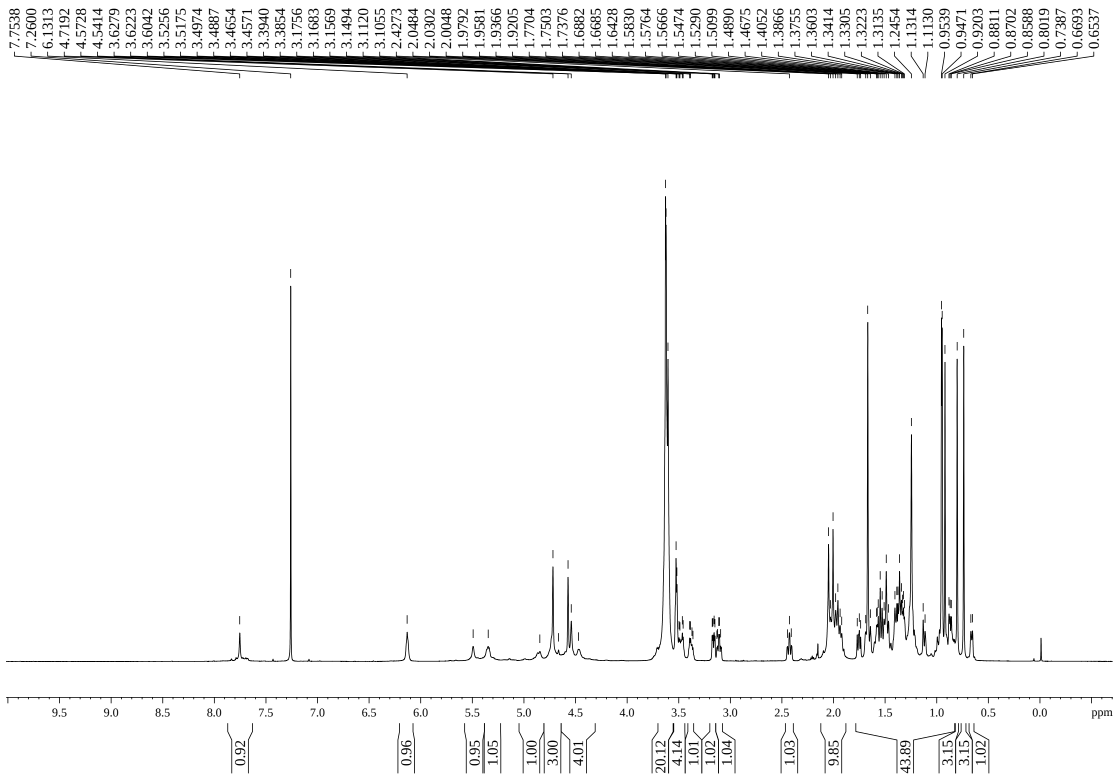

^{13}C NMR of compound **64**

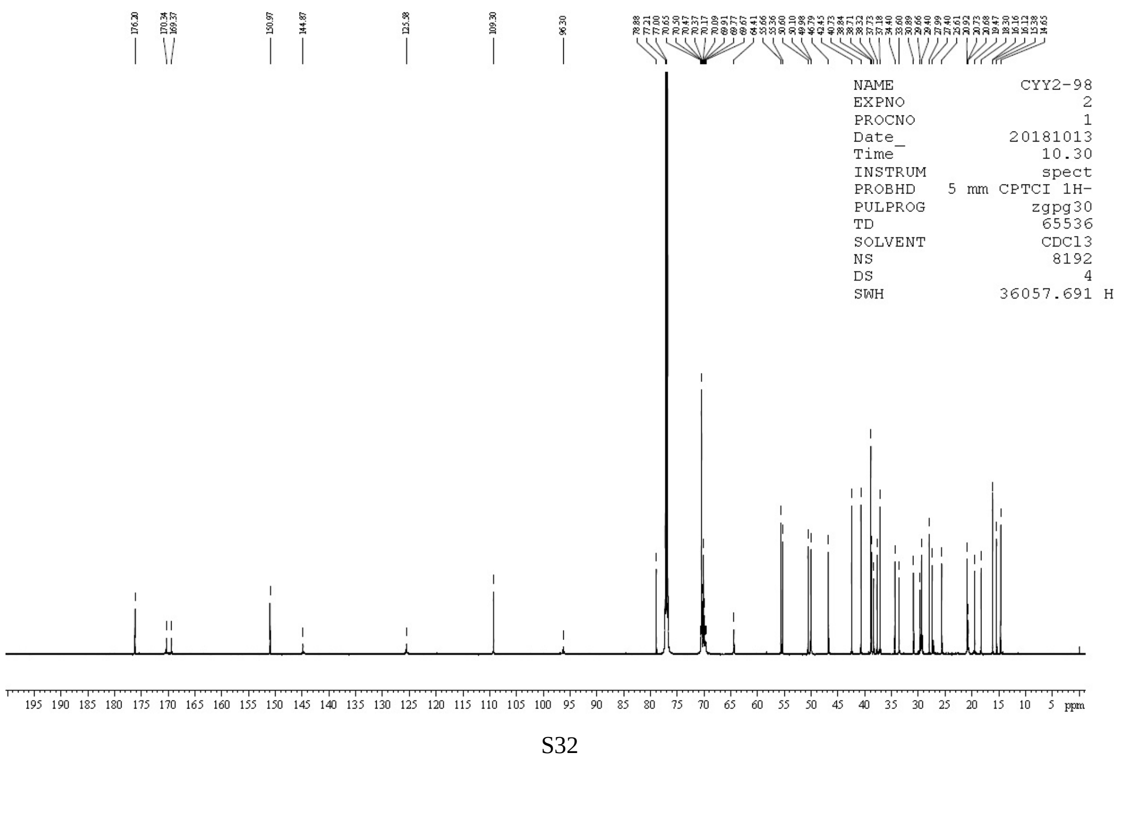

MALDI-TOF of compound **64**

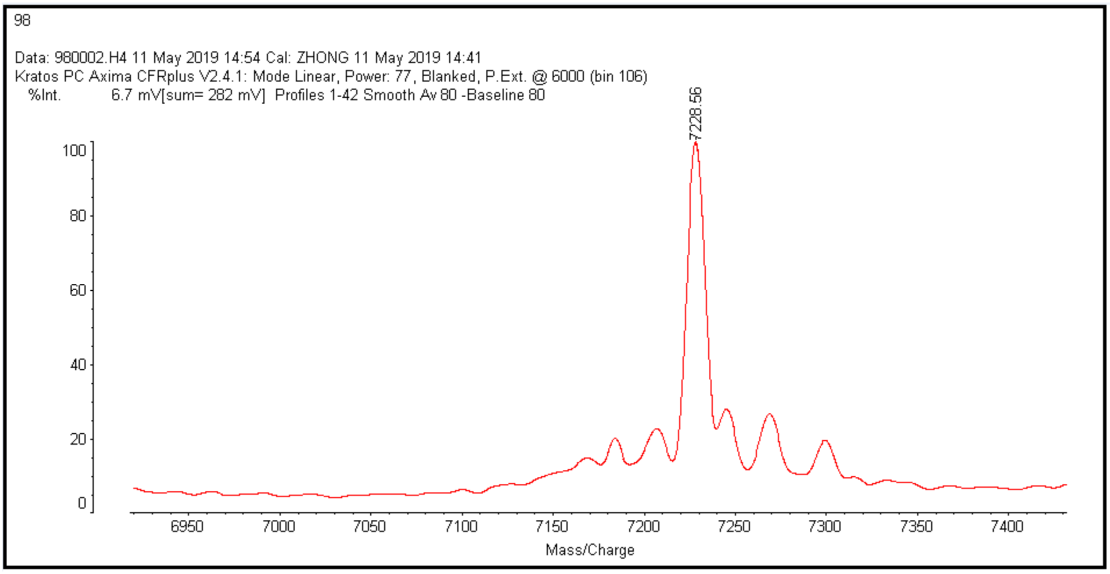

^{1}H NMR of compound **65**

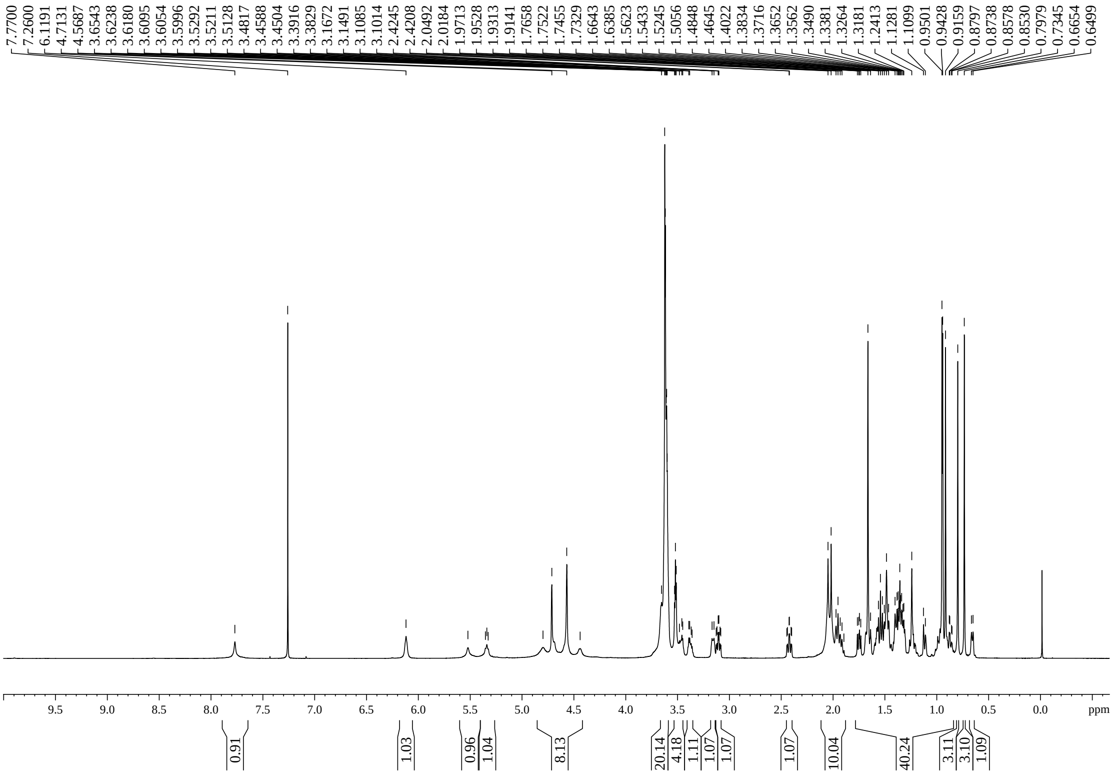

^{13}C NMR of compound **65**

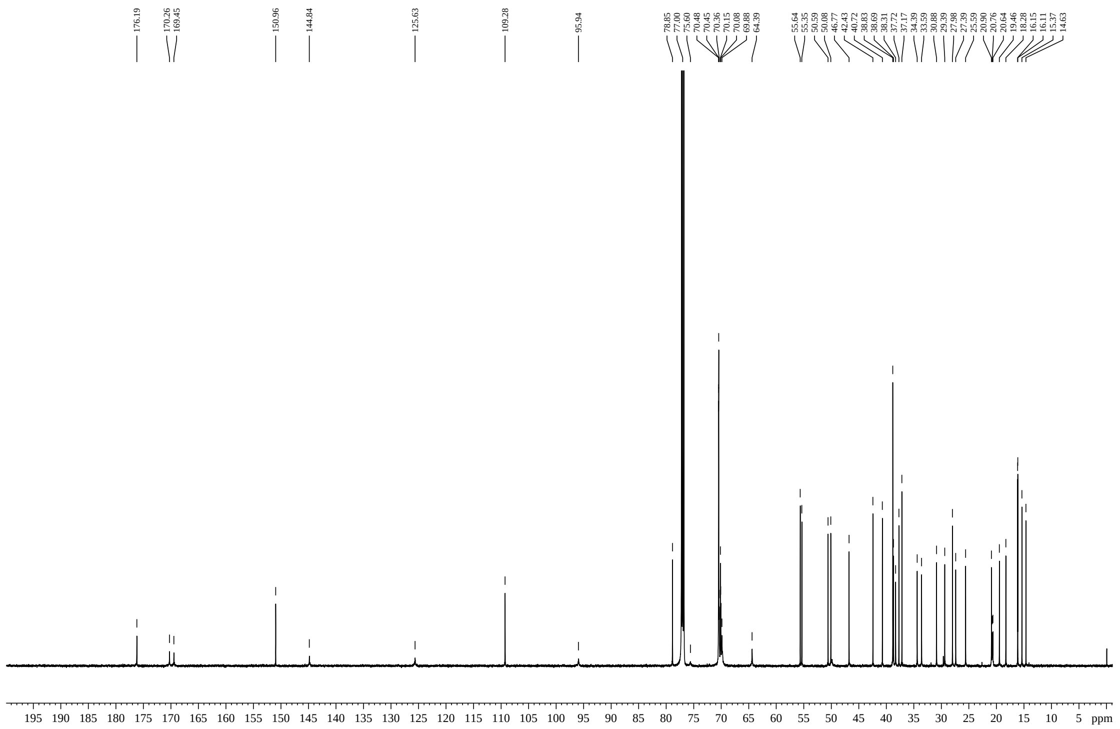

MALDI-TOF of compound **65**

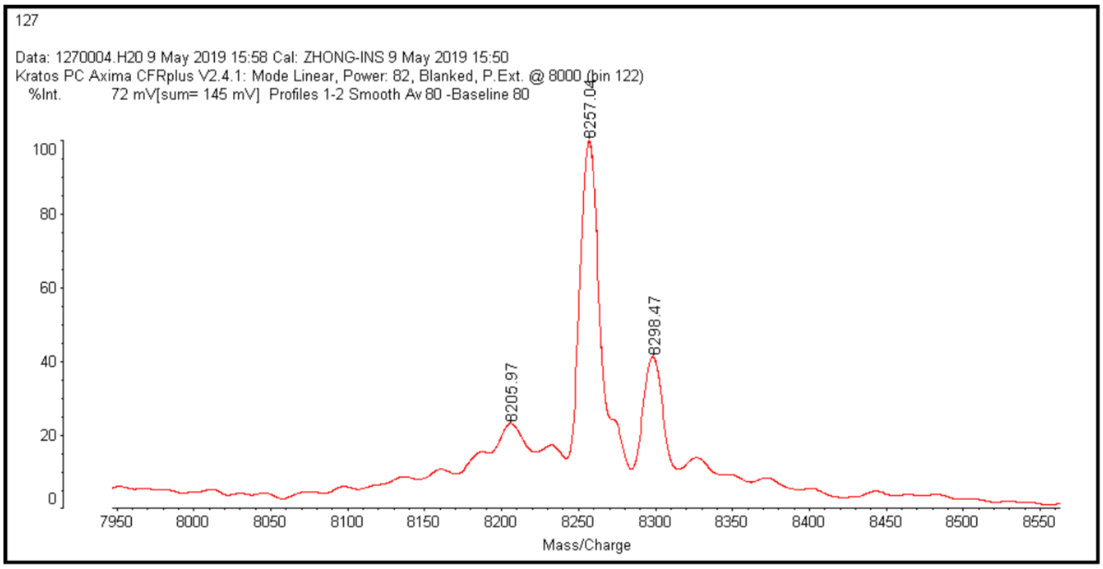

^{1}H NMR of compound **66**

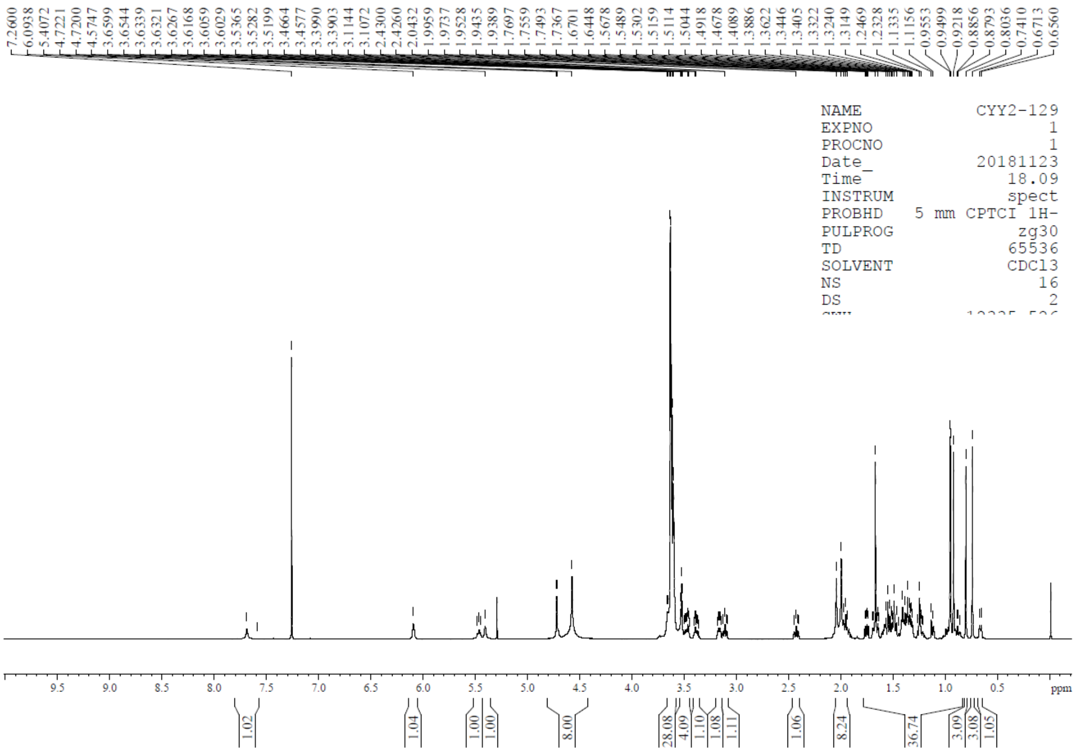

^{13}C NMR of compound **66**

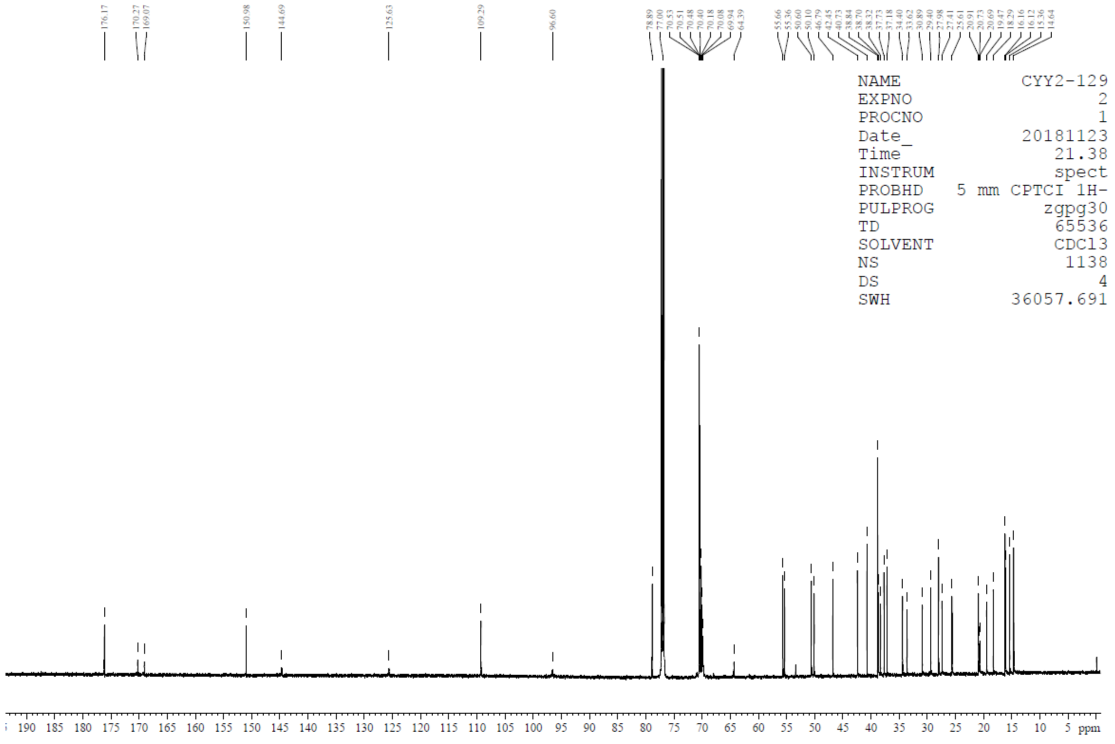

HRMS of compound **66**

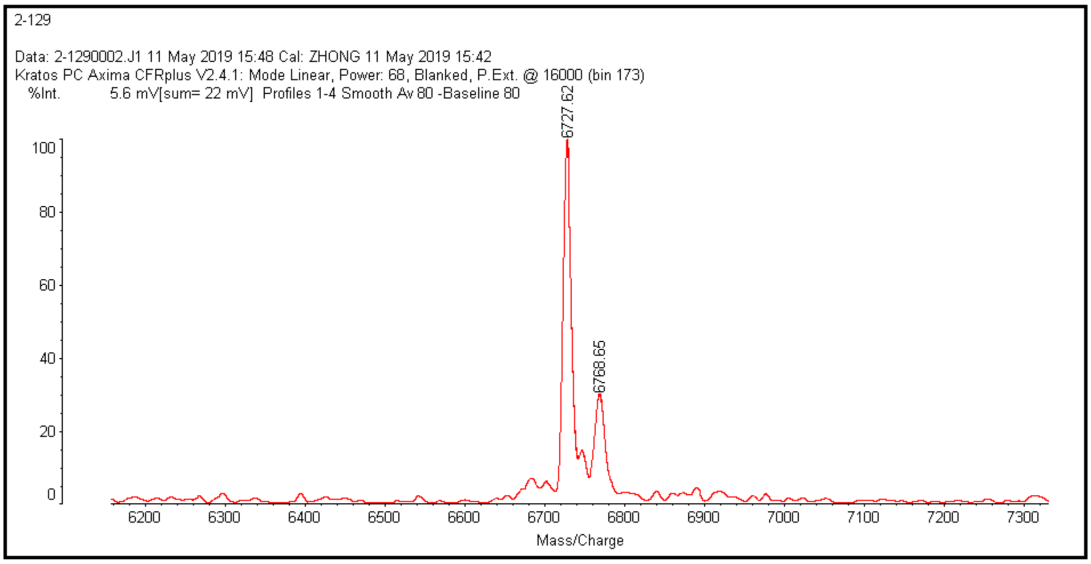

^{1}H NMR of compound **67**

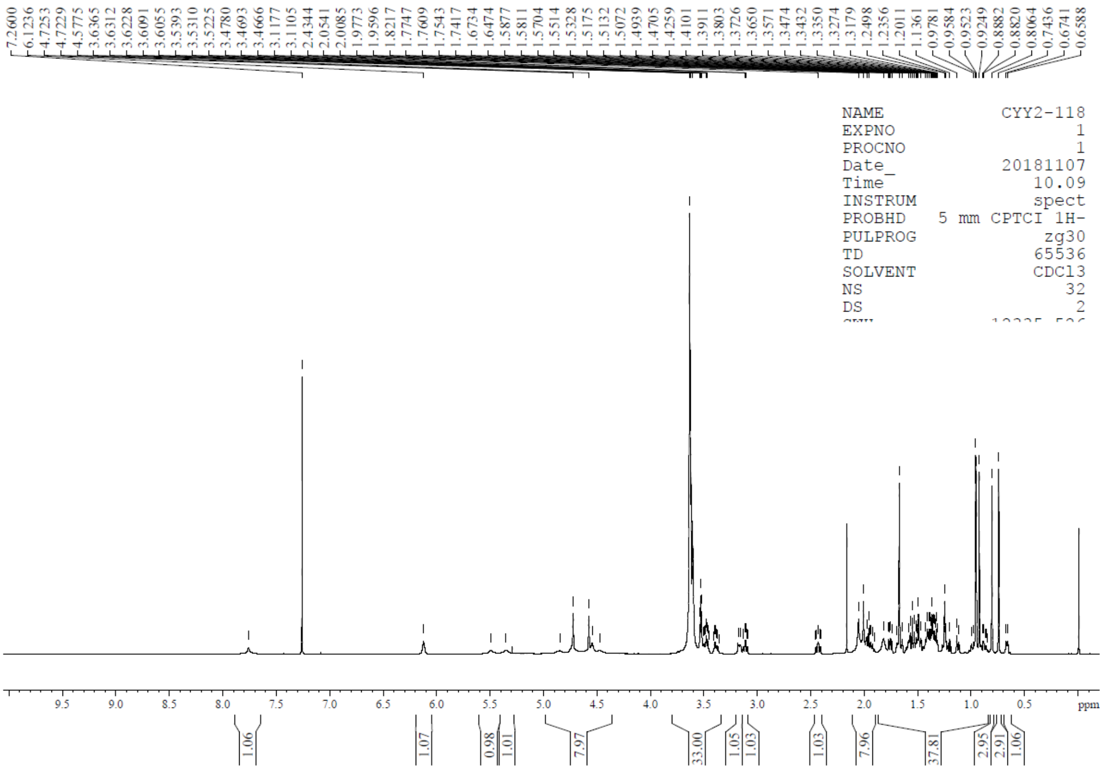

^{13}C NMR of compound **67**

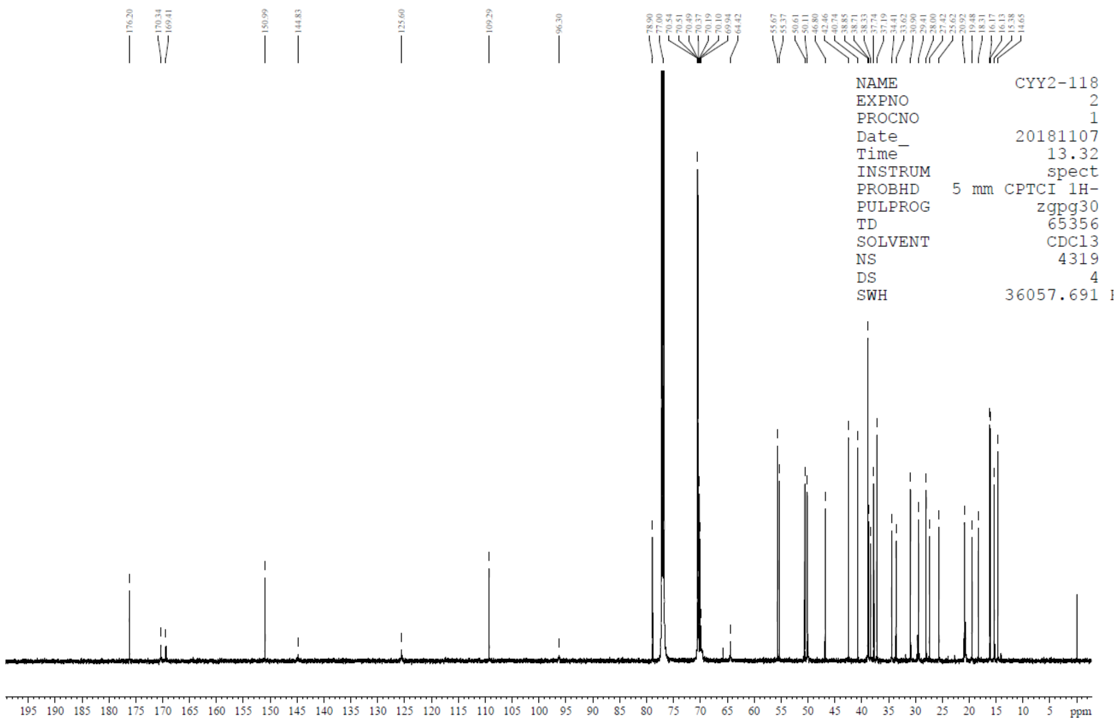

HRMS of compound **67**

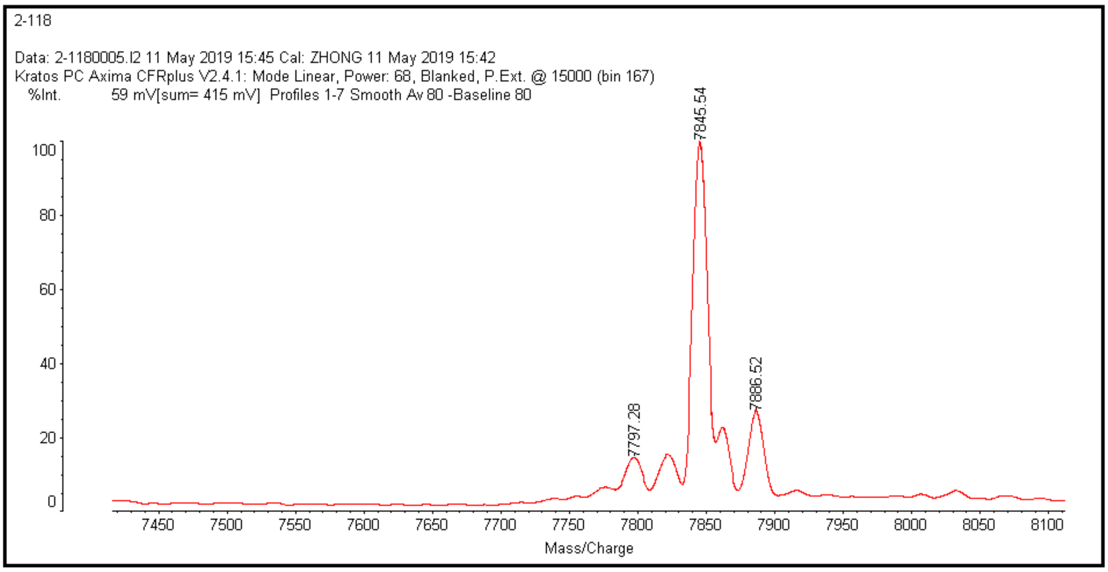

^{1}H NMR of compound **68**

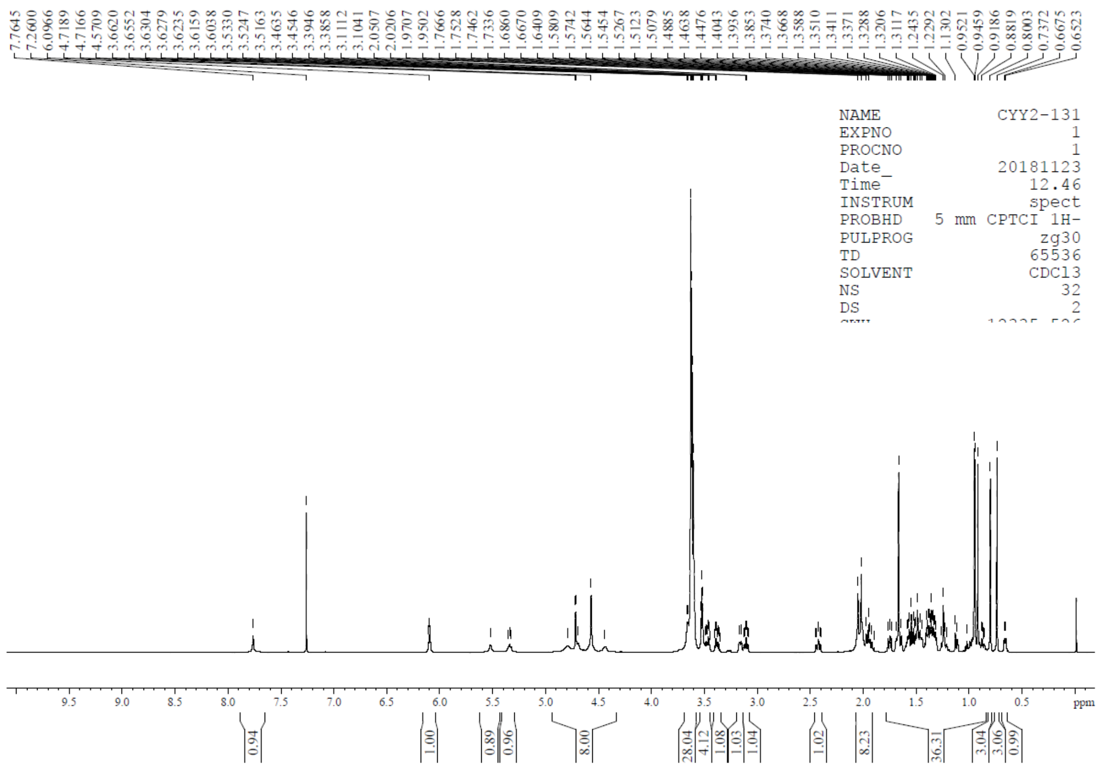

^{13}C NMR of compound **68**

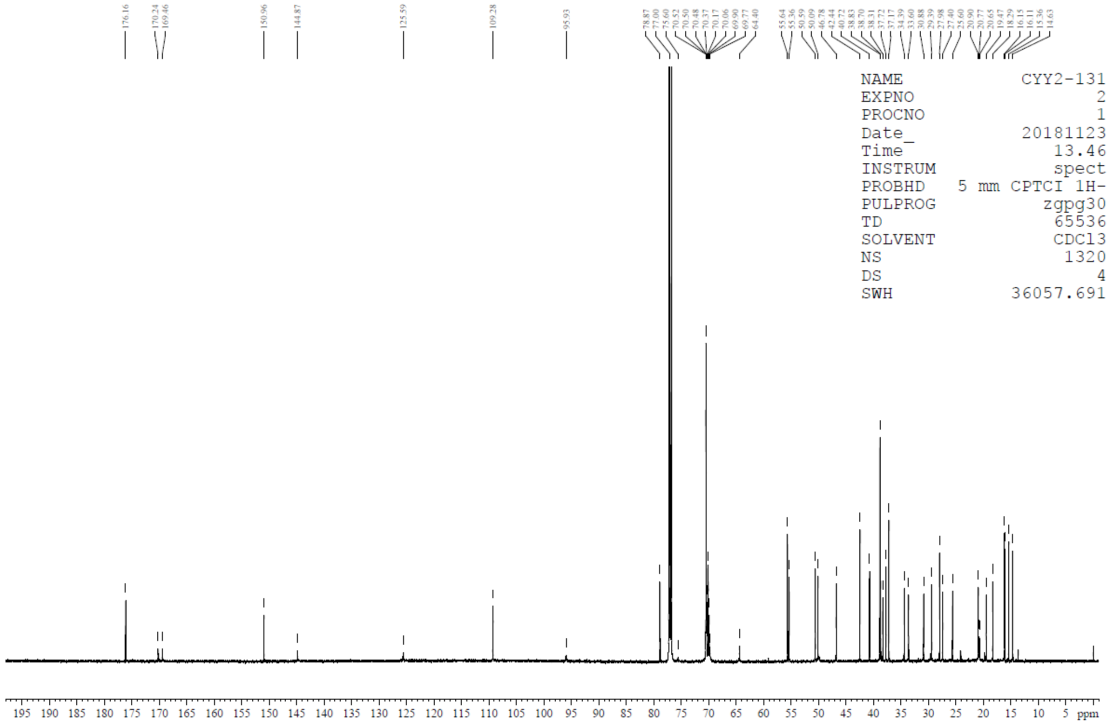

HRMS of compound **69**

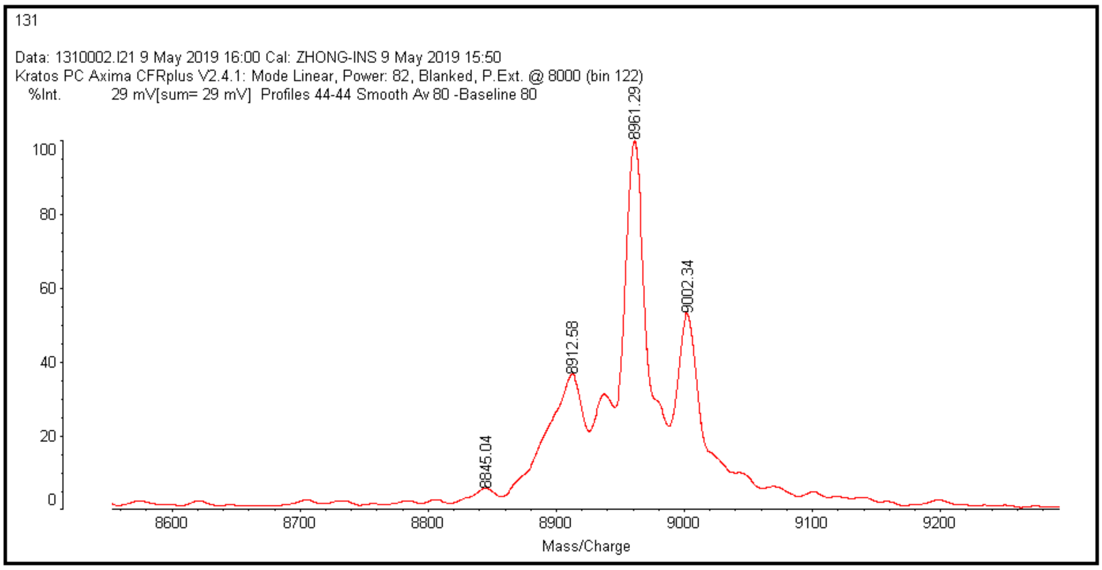

^{1}H NMR of compound **69**

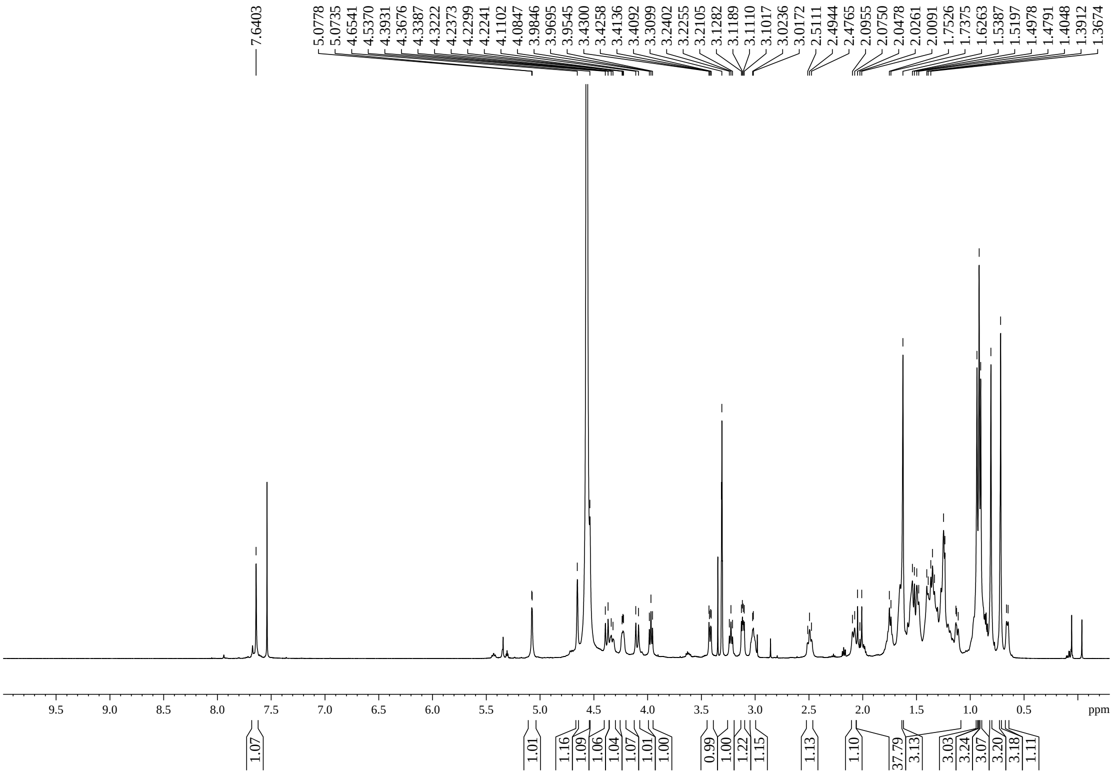

^{13}C NMR of compound **69**

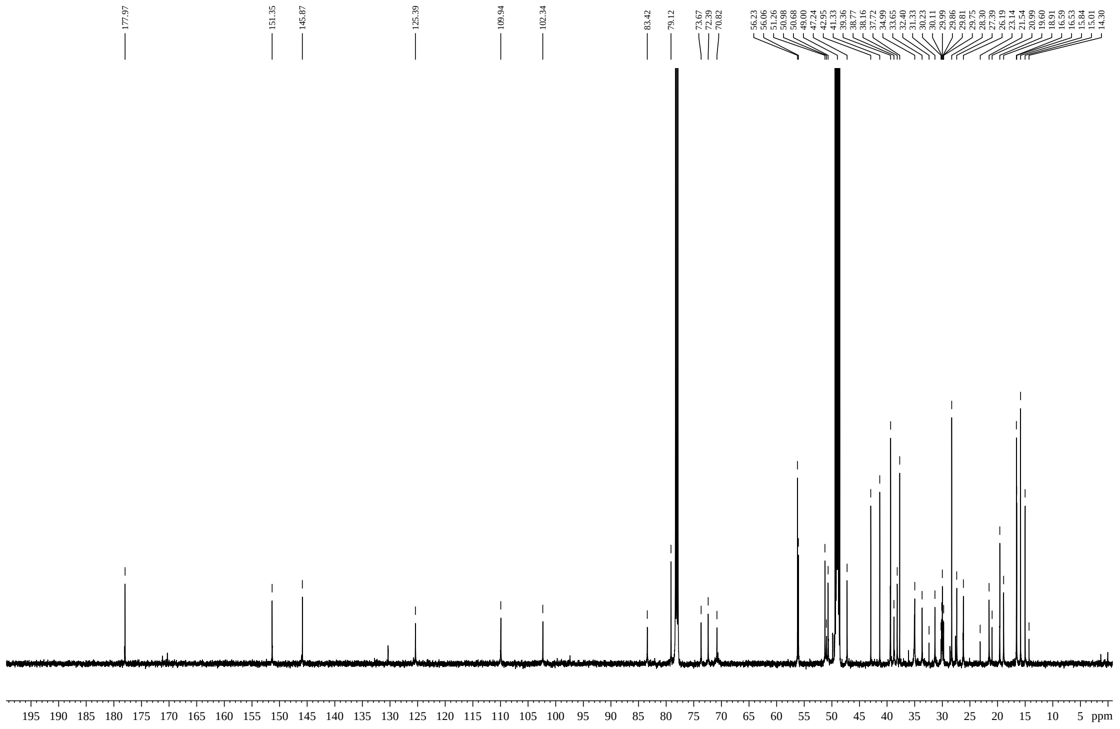

MALDI-TOF of compound **69**

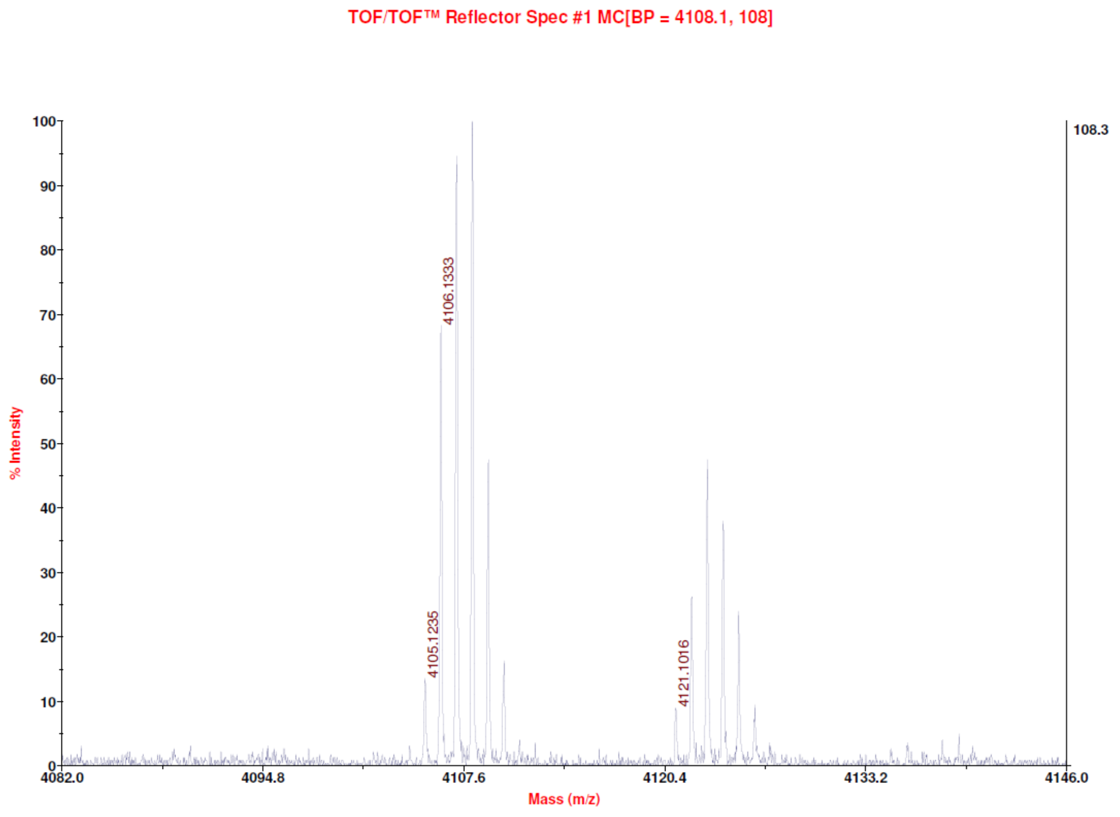

^{1}H NMR of compound **70**

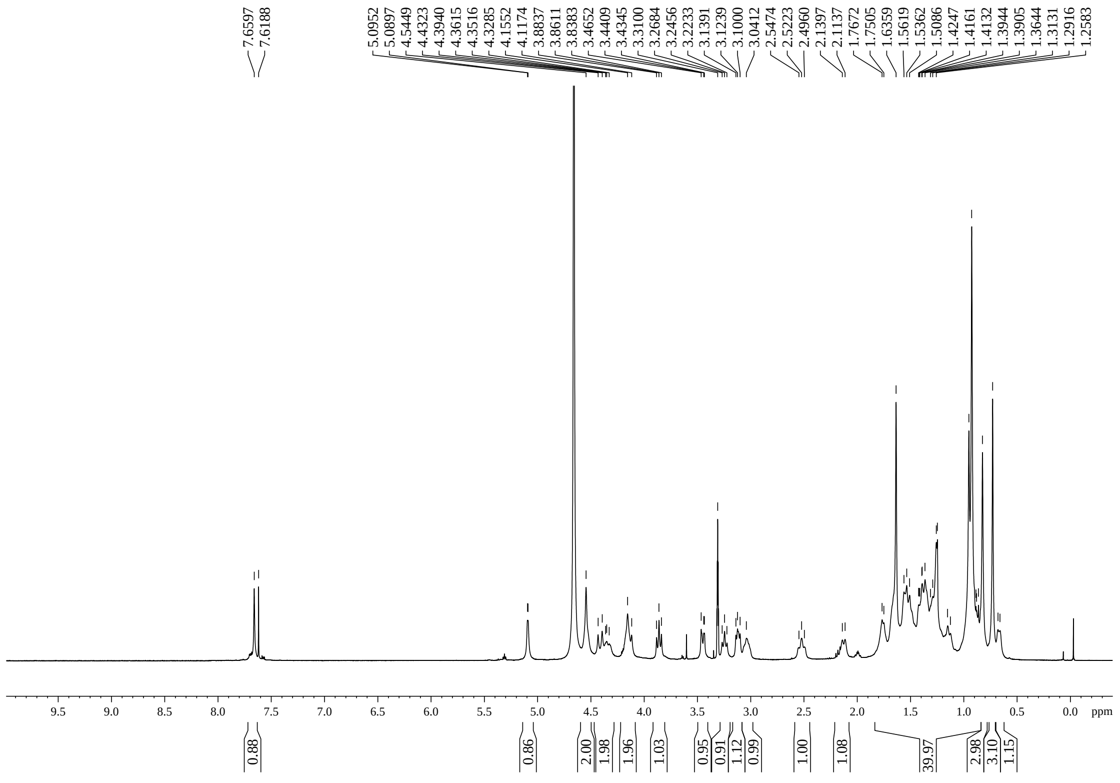

^{13}C NMR of compound **70**

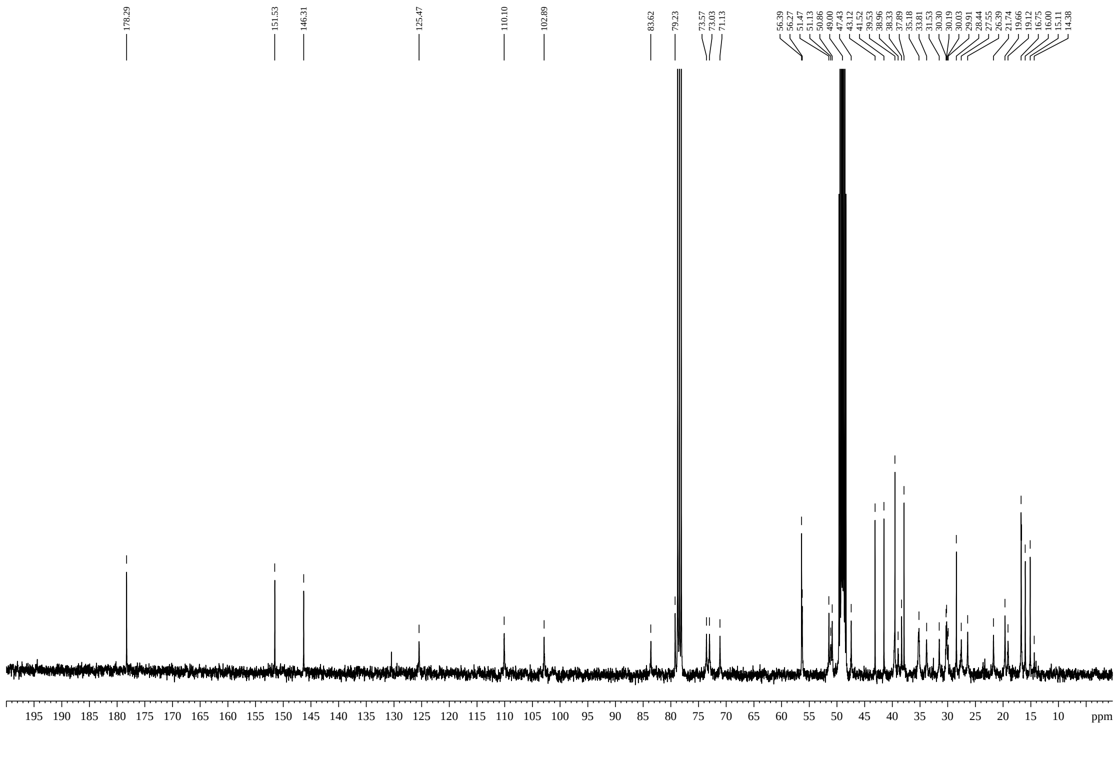

MALDI-TOF of compound **70**

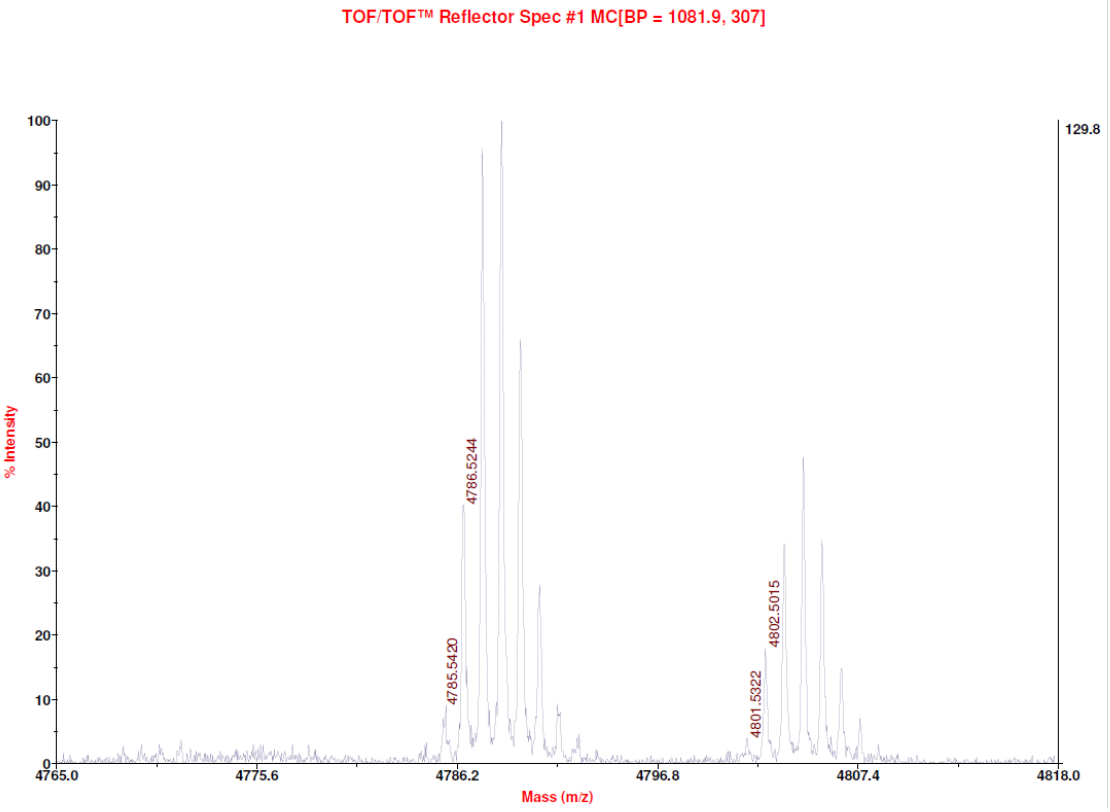

^{1}H NMR of compound **71**

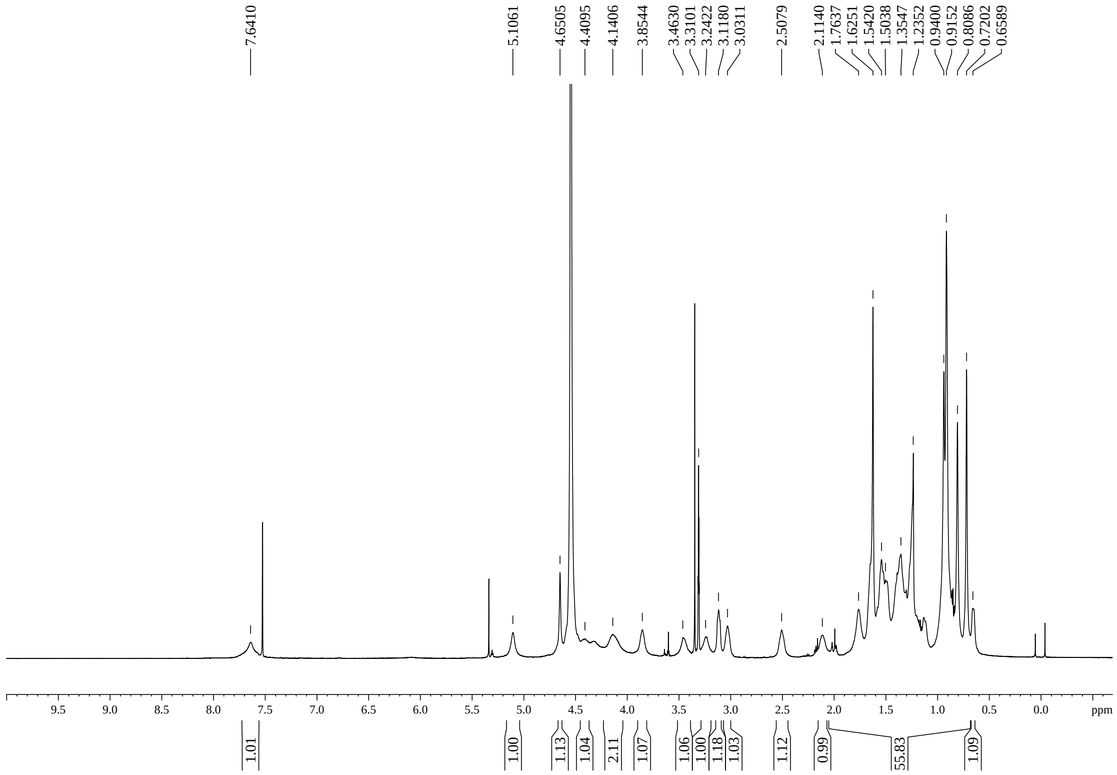

^{13}C NMR of compound **71**

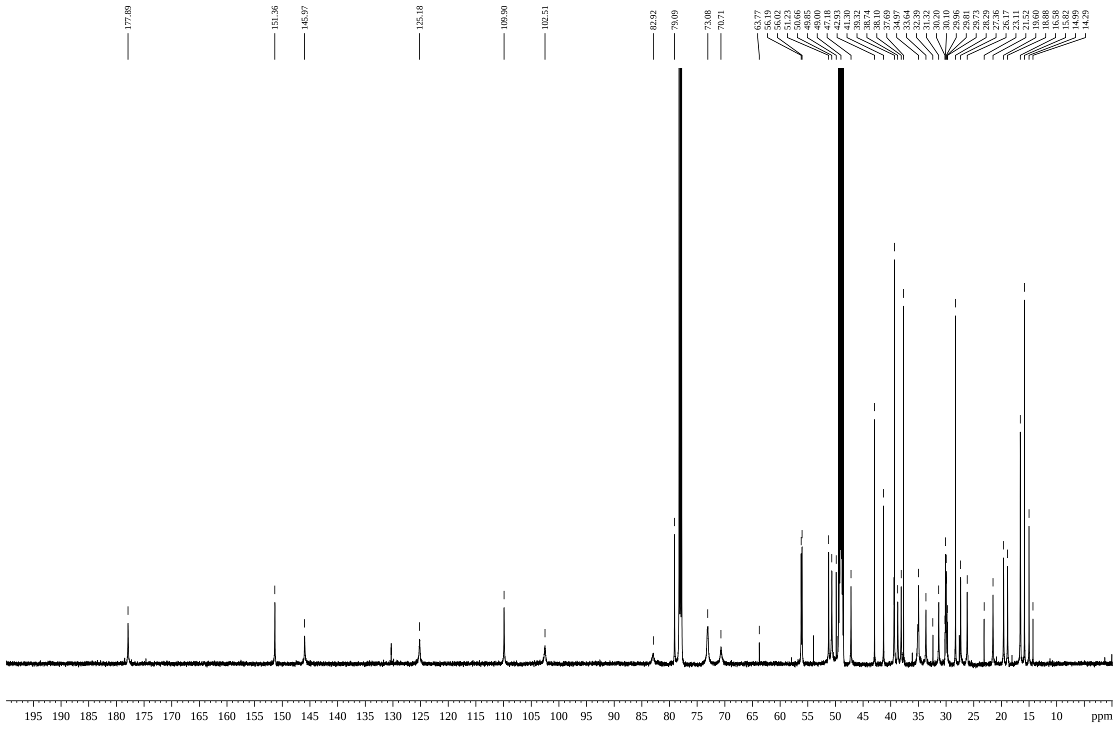

MALDI-TOF of compound **71**

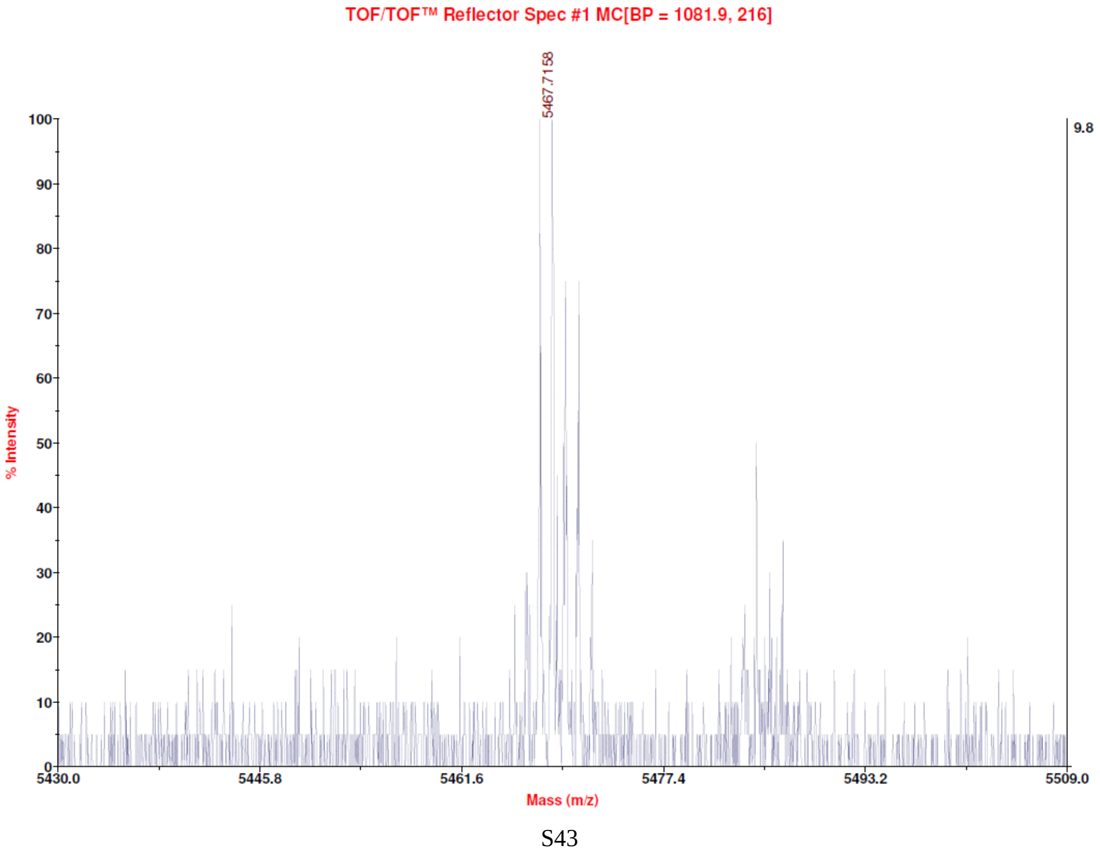

^{1}H NMR of compound **72**

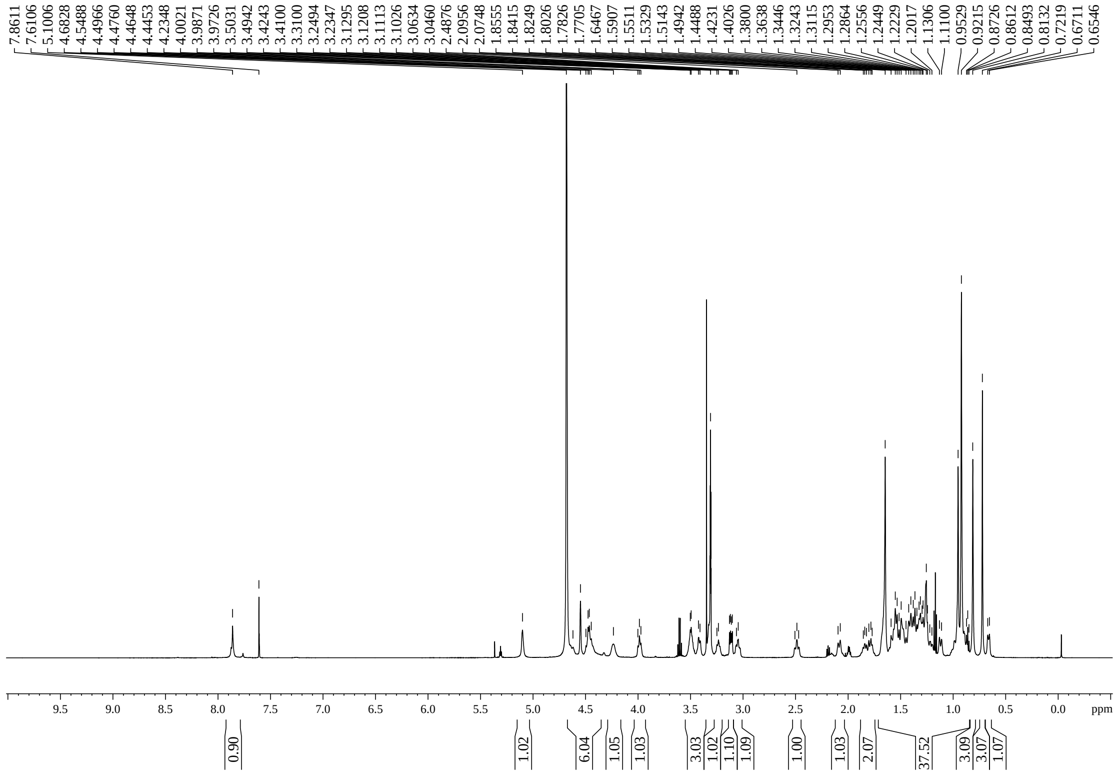

^{13}C NMR of compound **72**

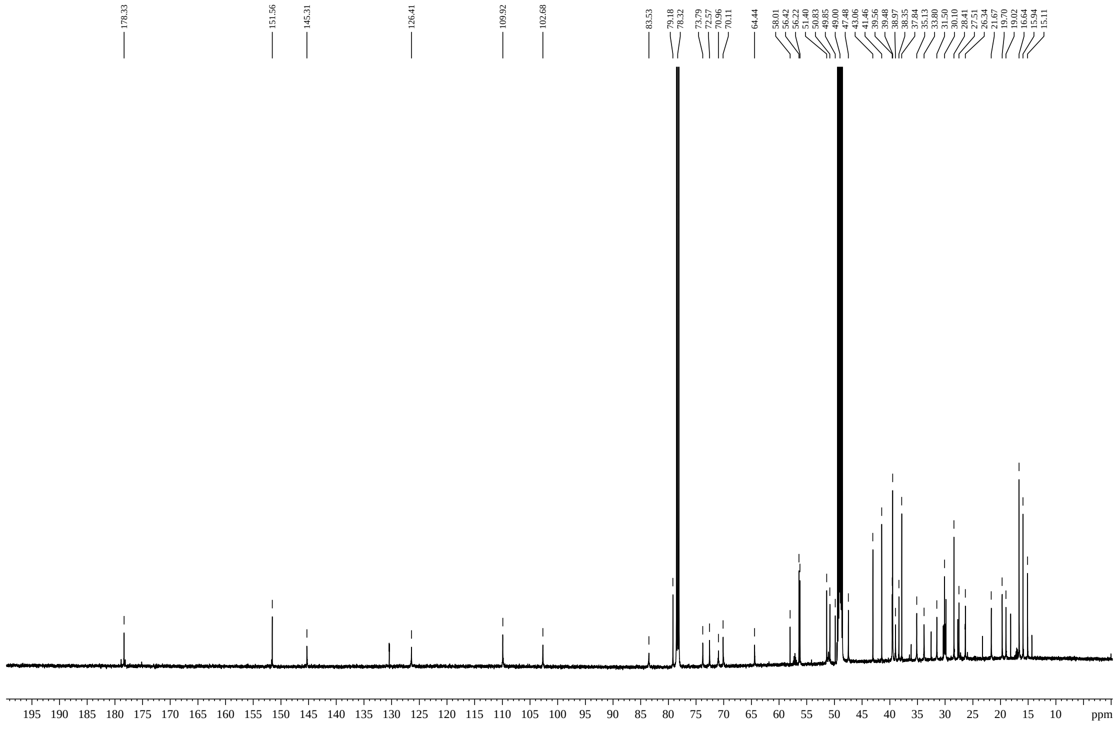

MALDI-TOF of compound **72**

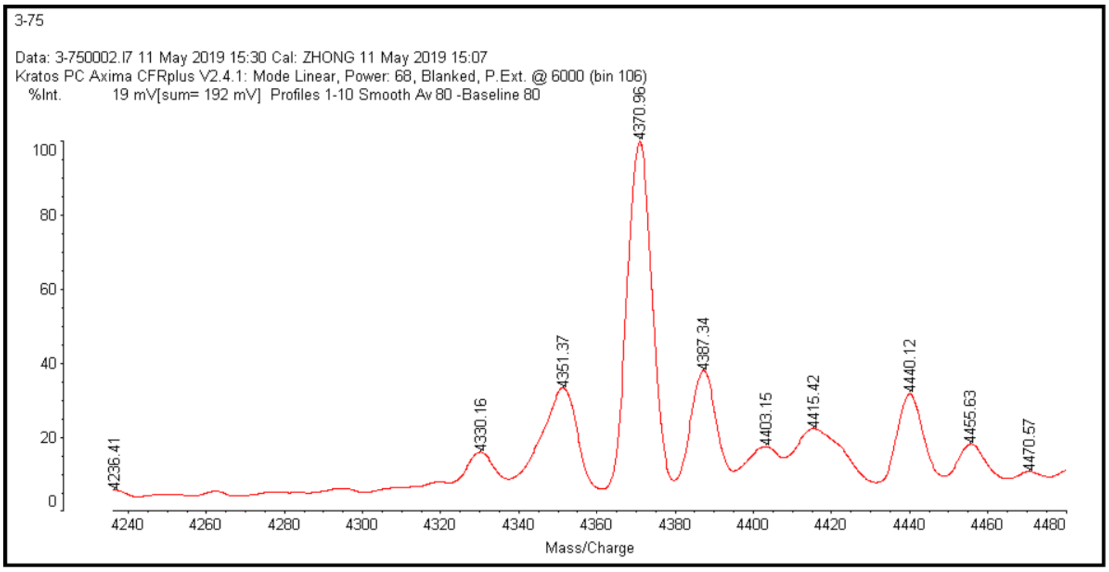

^{1}H NMR of compound **73**

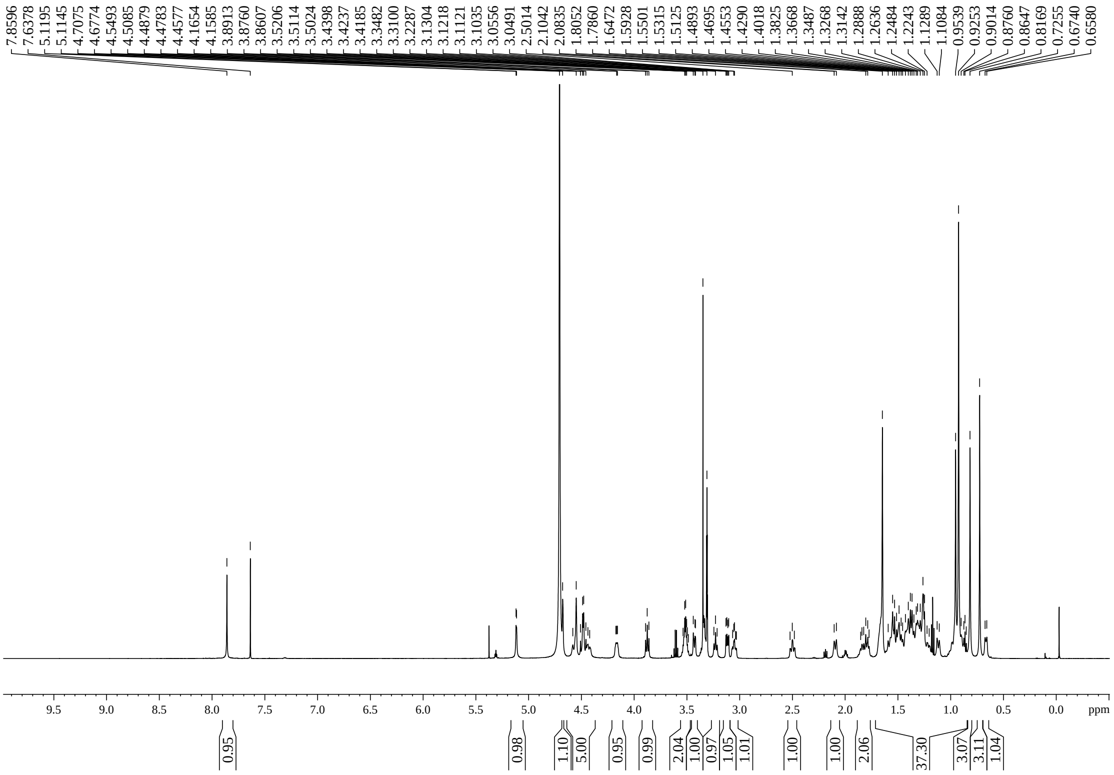

^{13}C NMR of compound **73**

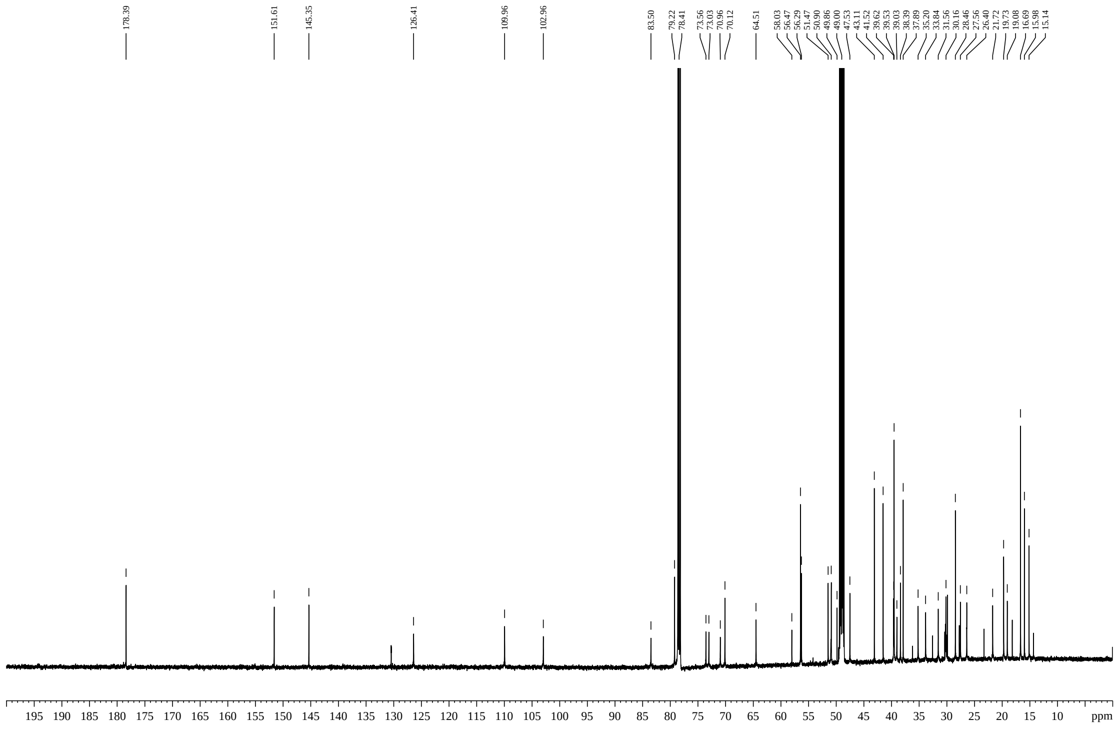

MALDI-TOF of compound **73**

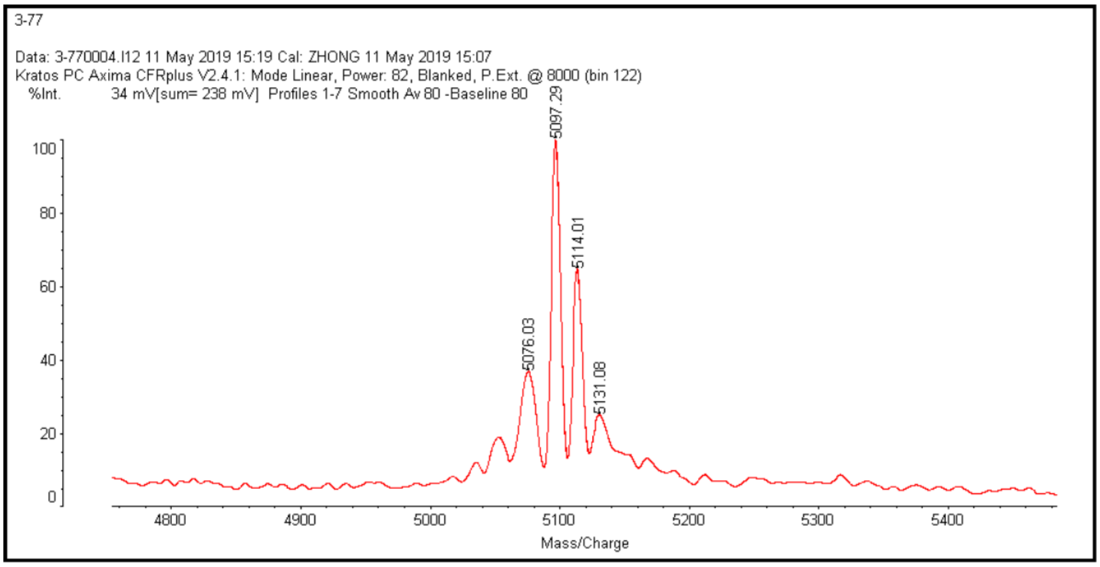

^{1}H NMR of compound **74**

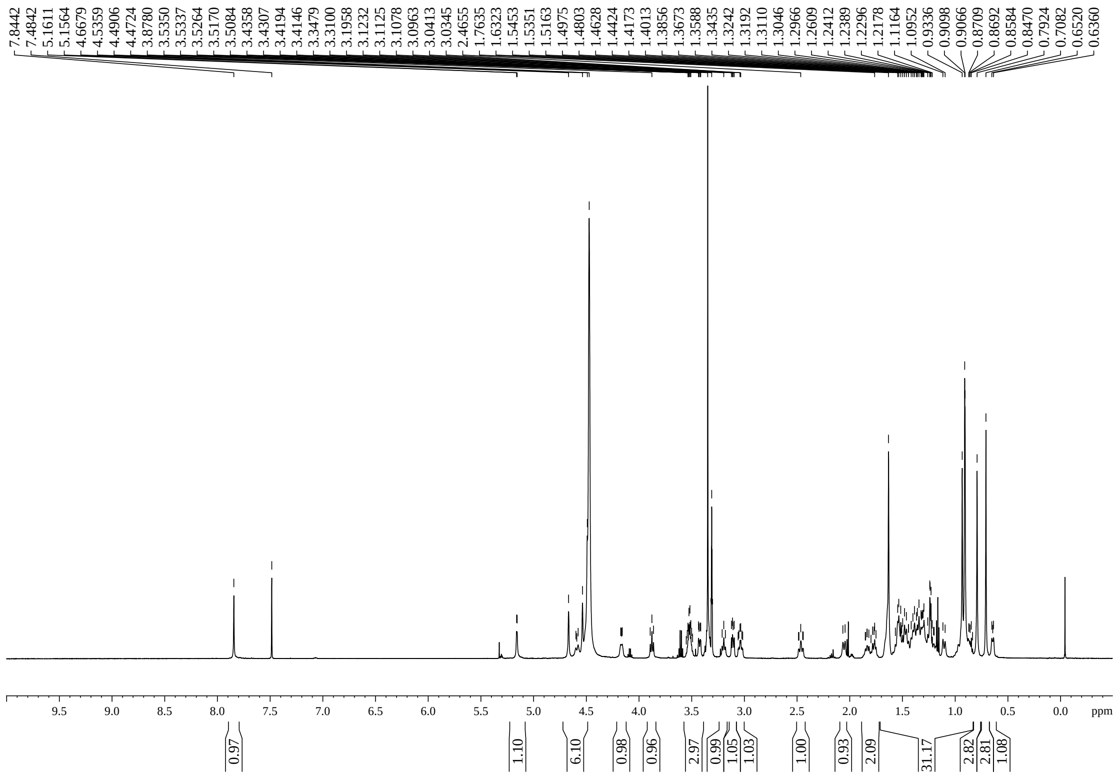

^{13}C NMR of compound **74**

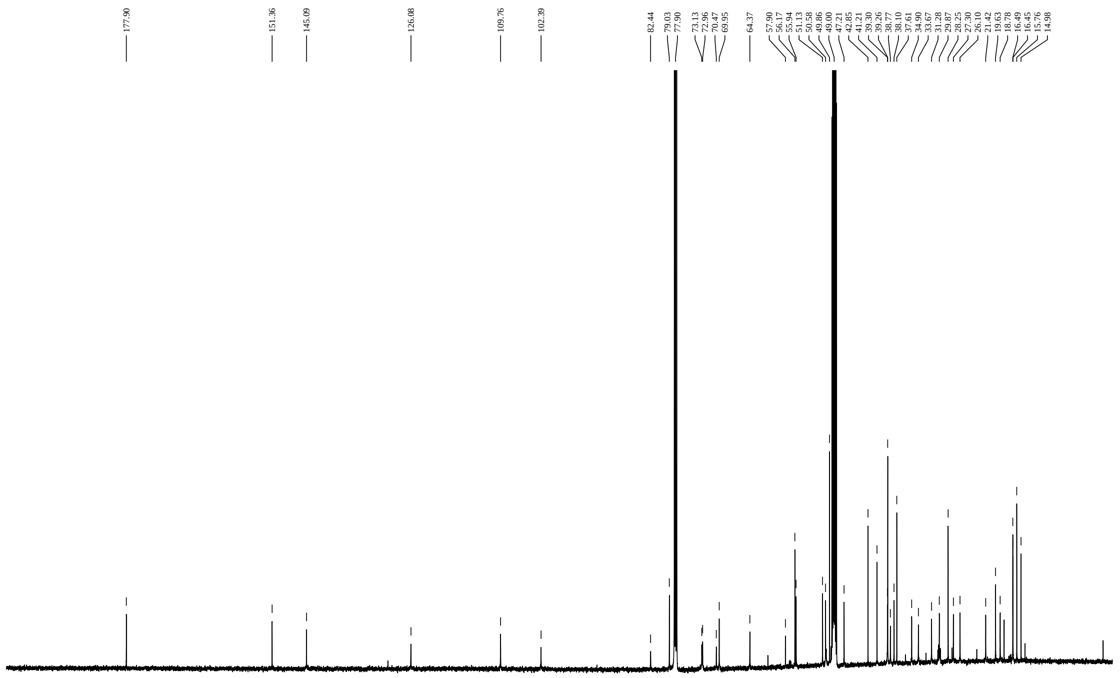

MALDI-TOF of compound **74**

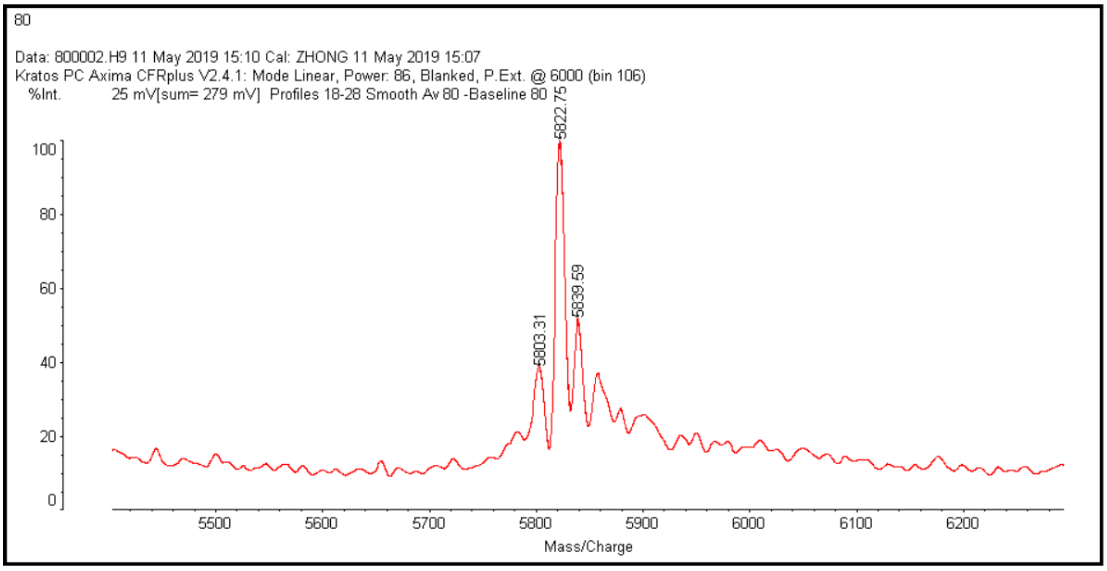

^{1}H NMR of compound **75**

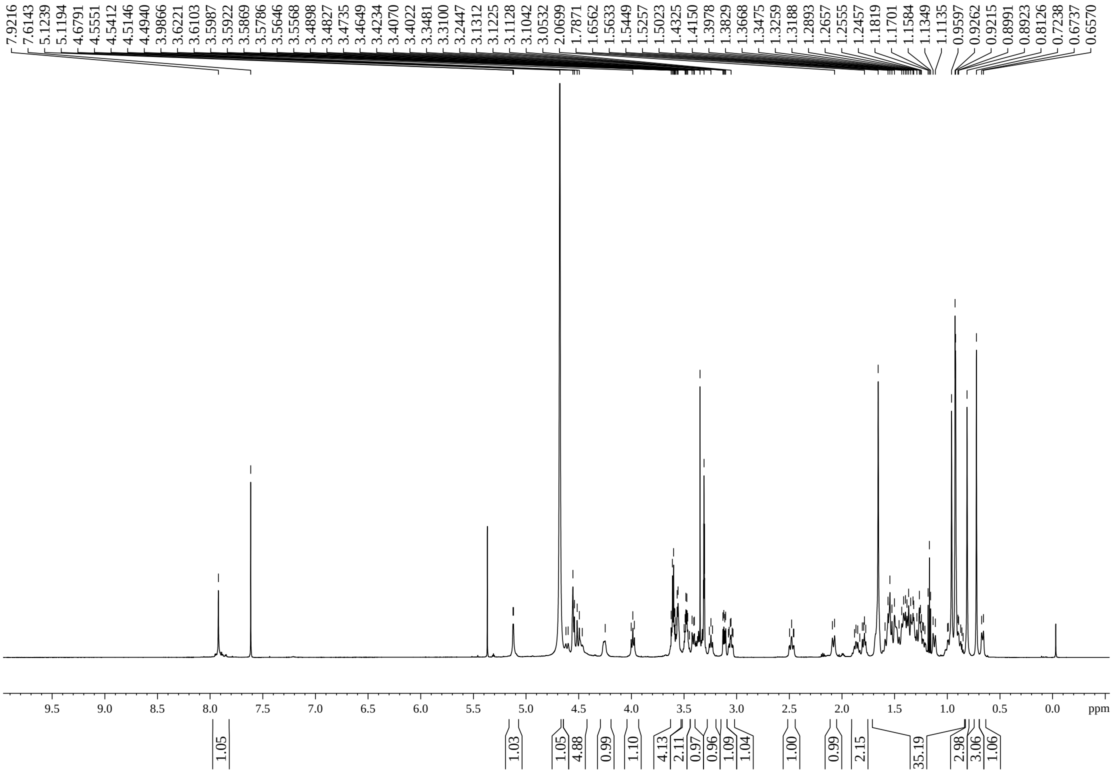

^{13}C NMR of compound **75**

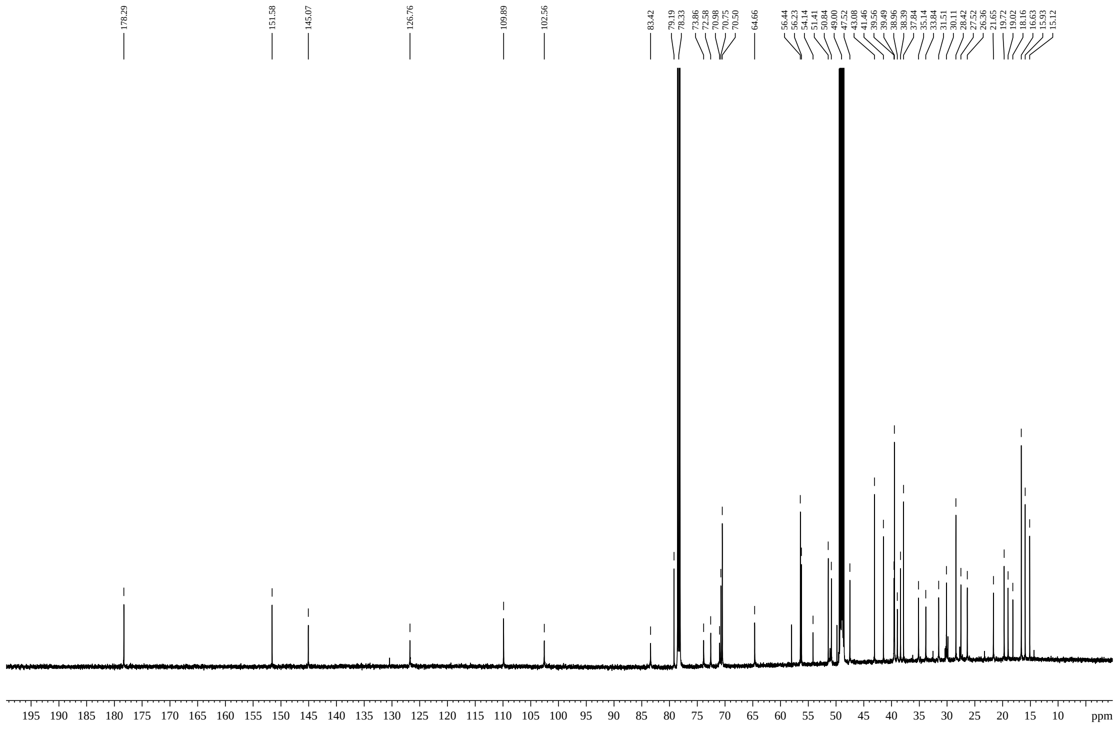

MALDI-TOF of compound **75**

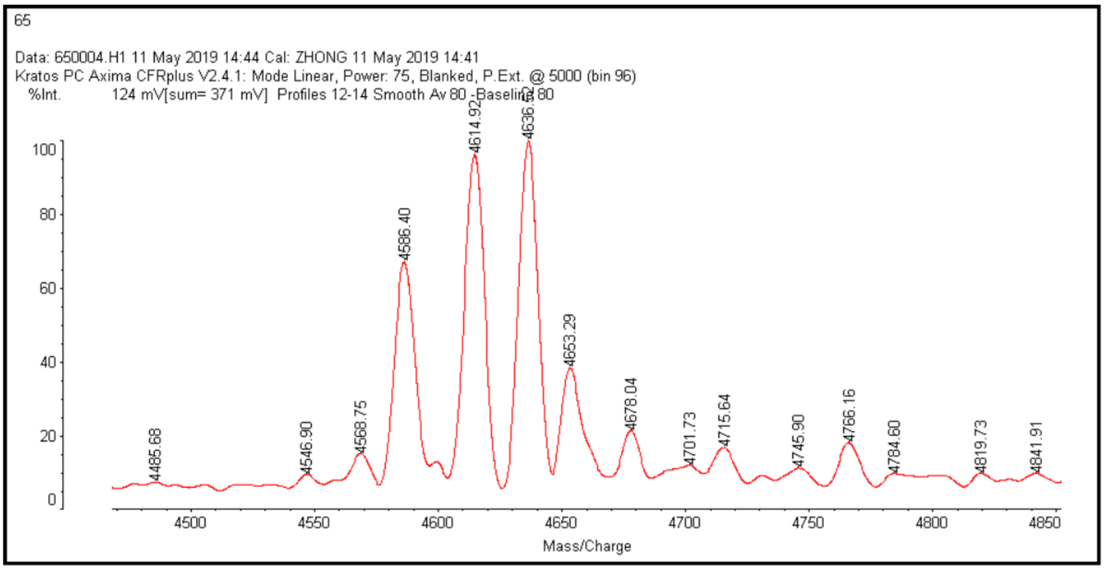

^{1}H NMR of compound **76**

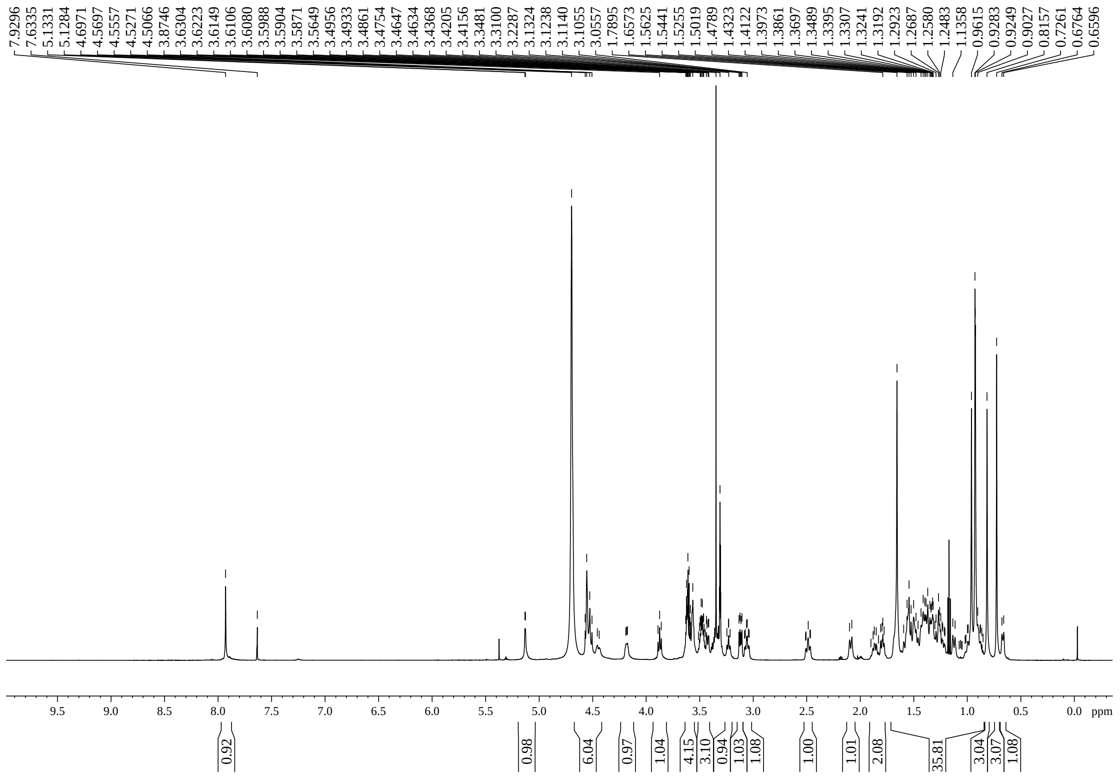

^{13}C NMR of compound **76**

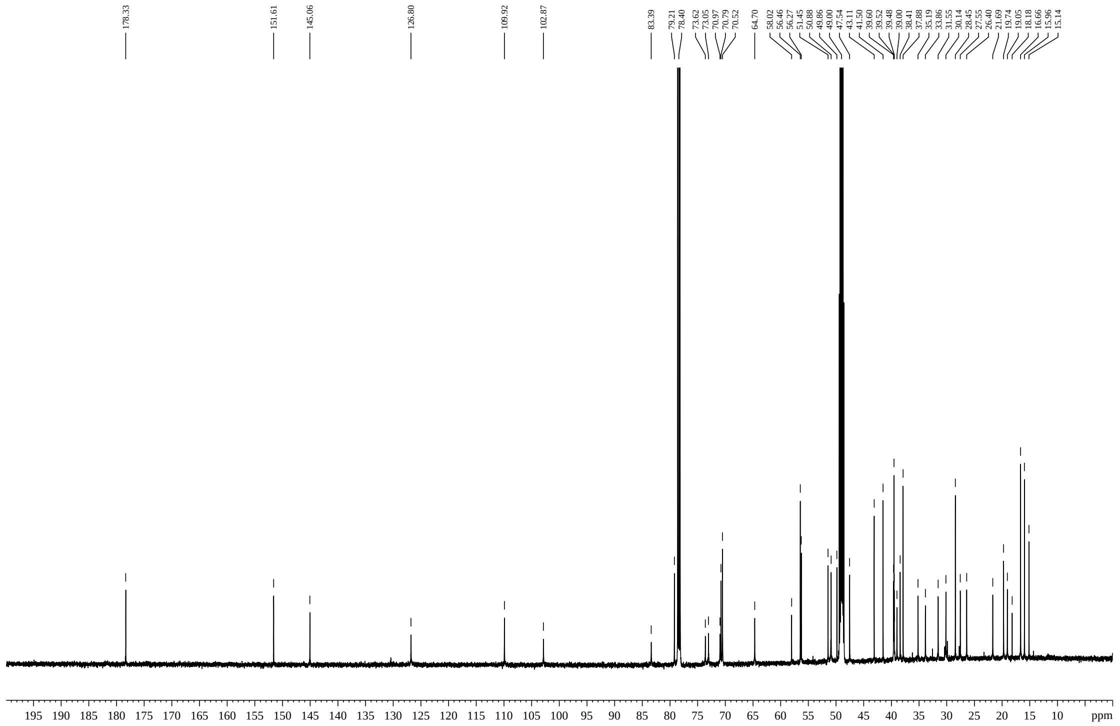

MALDI-TOF of compound **76**

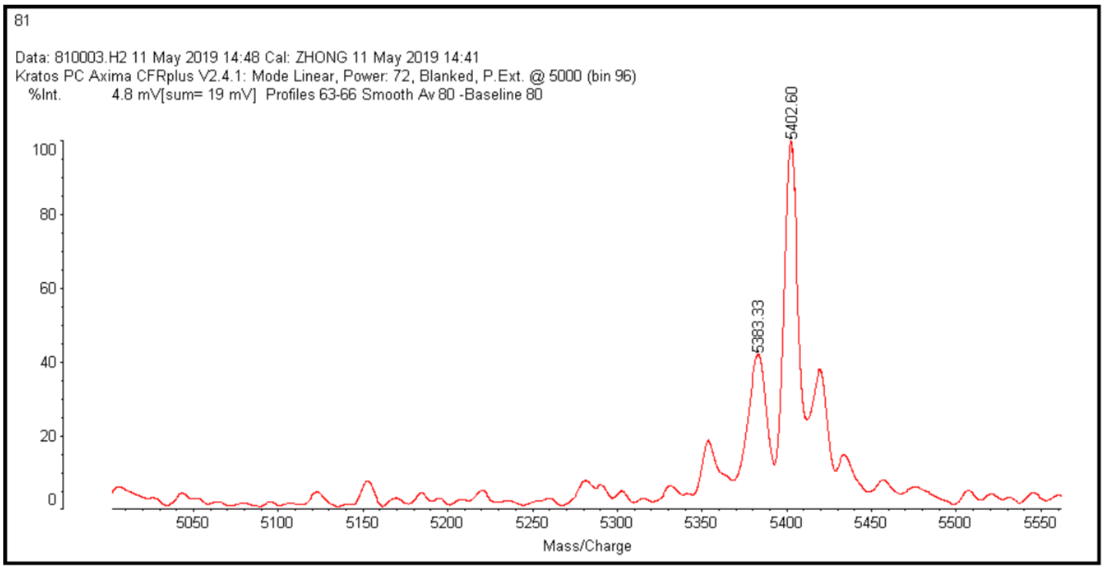

^{1}H NMR of compound **77**

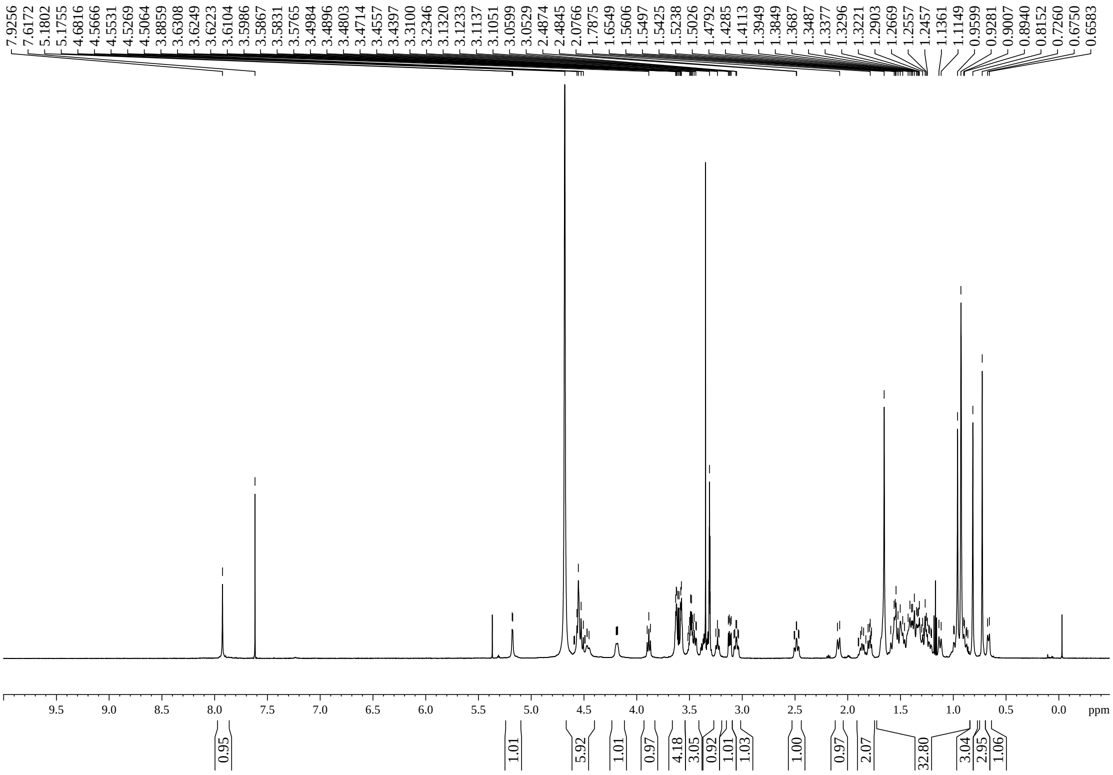

^{13}C NMR of compound **77**

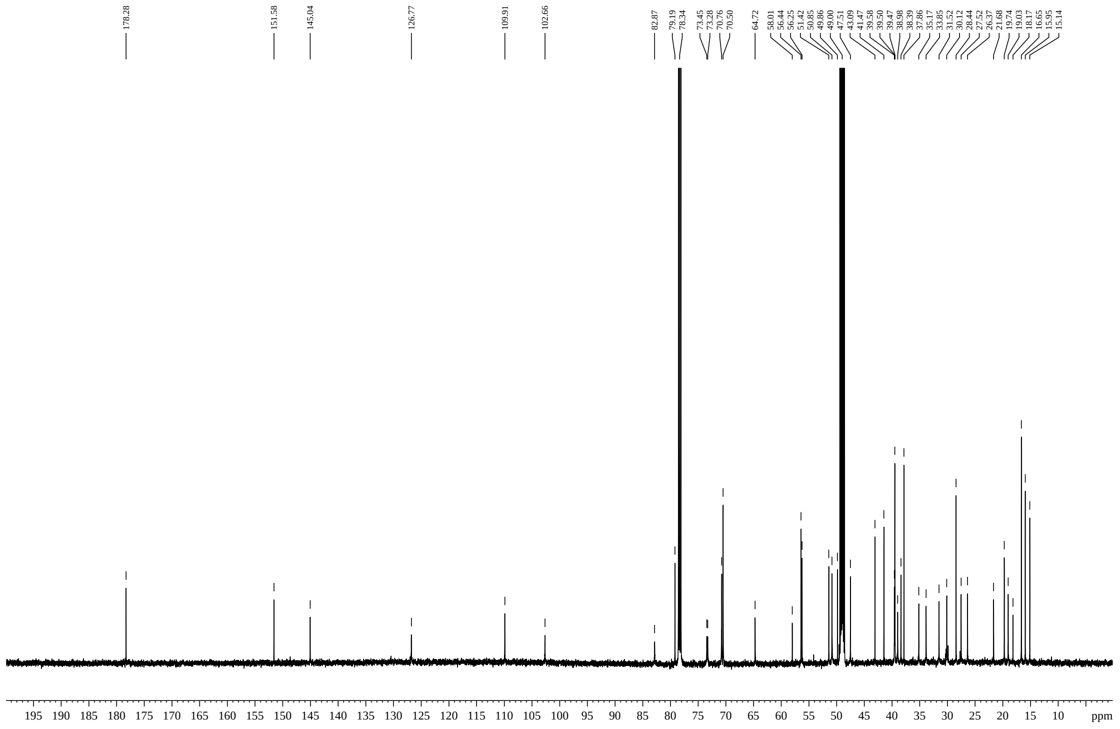

MALDI-TOF of compound **77**

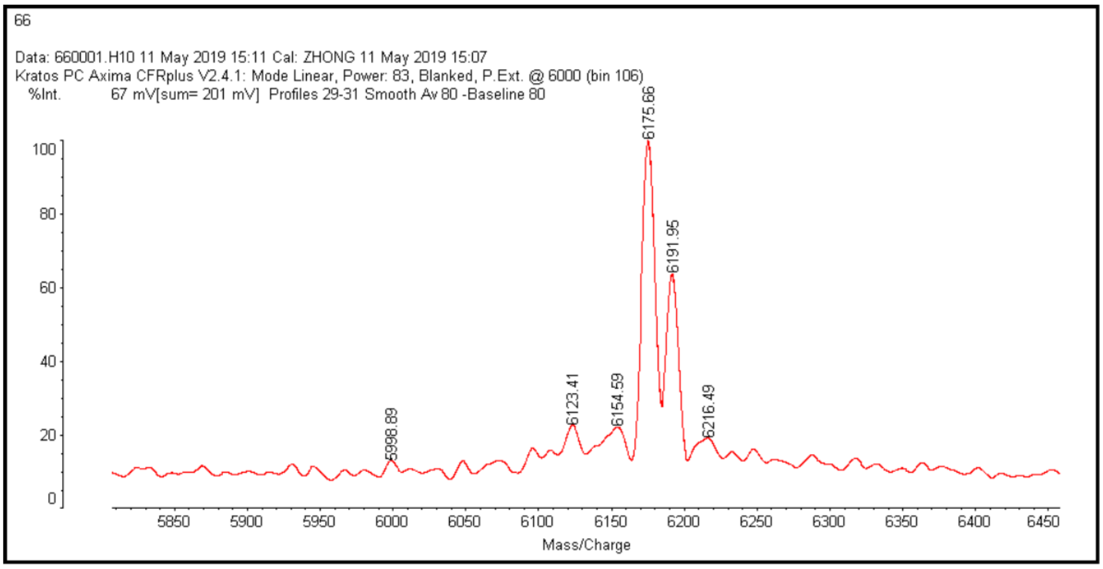

^{1}H NMR of compound **78**

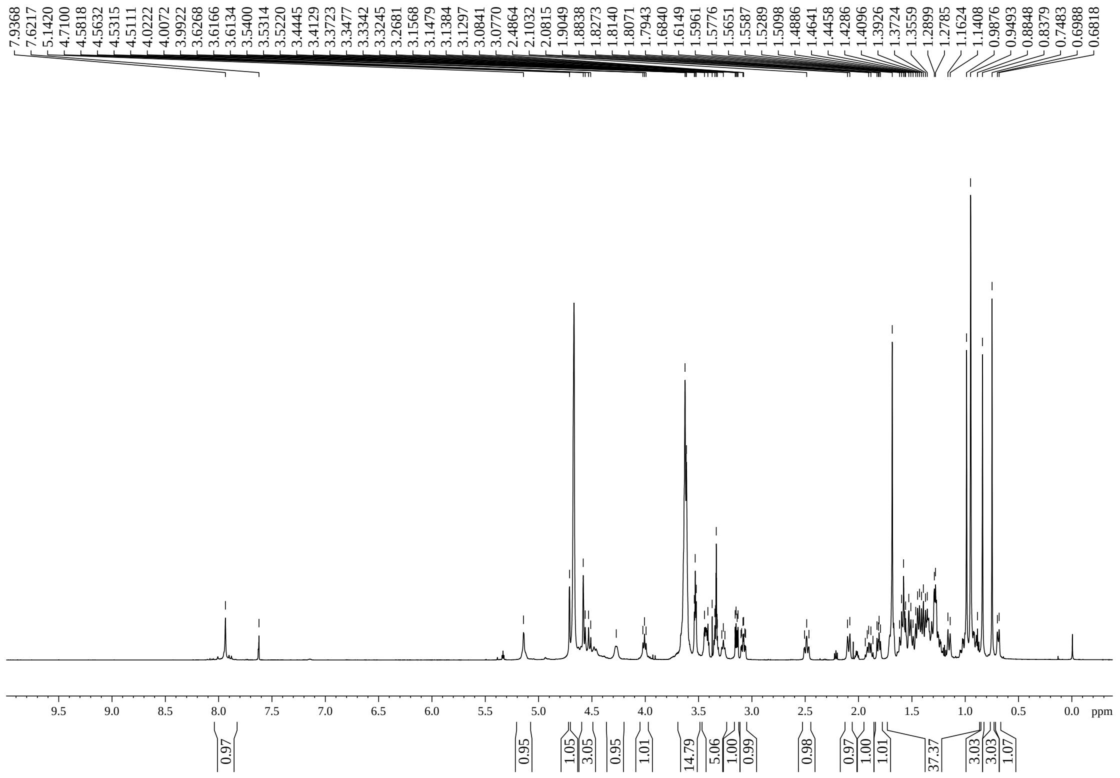

^{13}C NMR of compound **78**

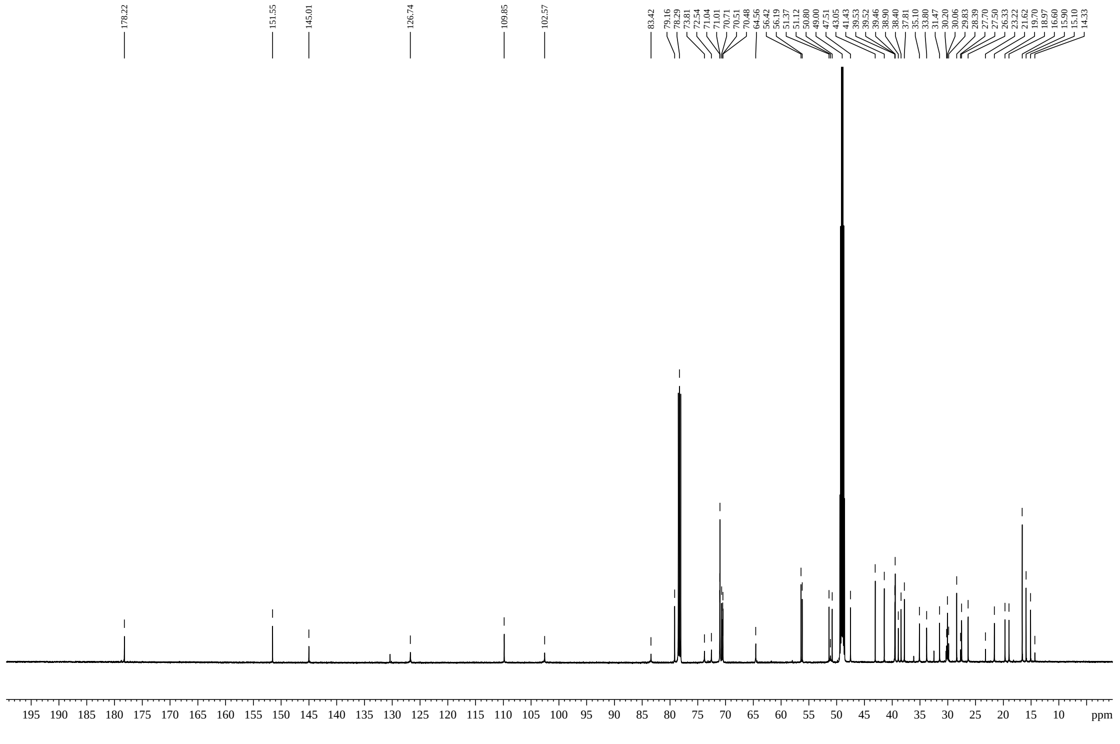

MALDI-TOF of compound **78**

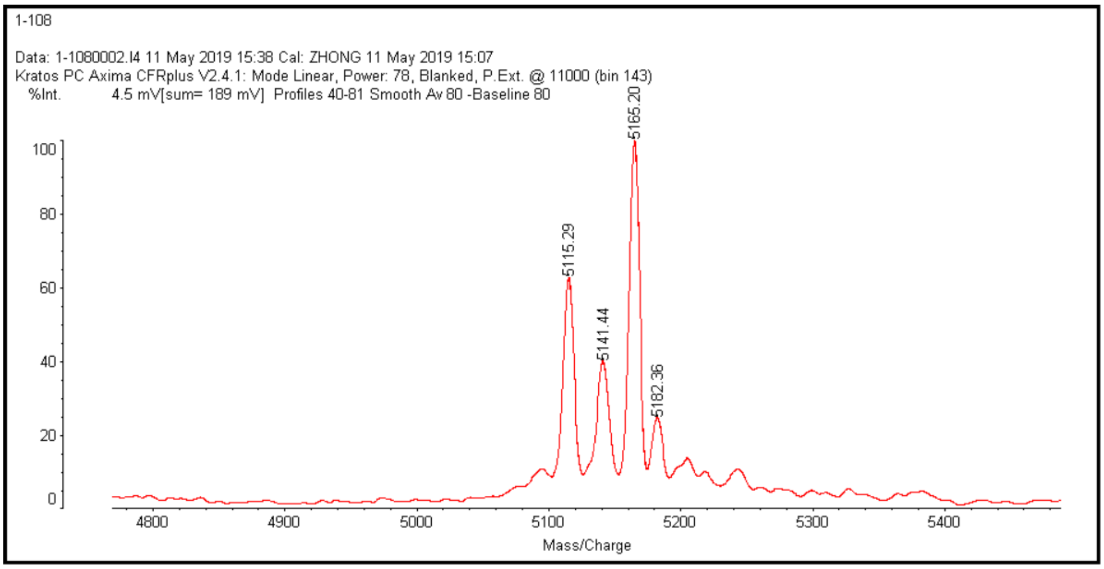

^{1}H NMR of compound **79**

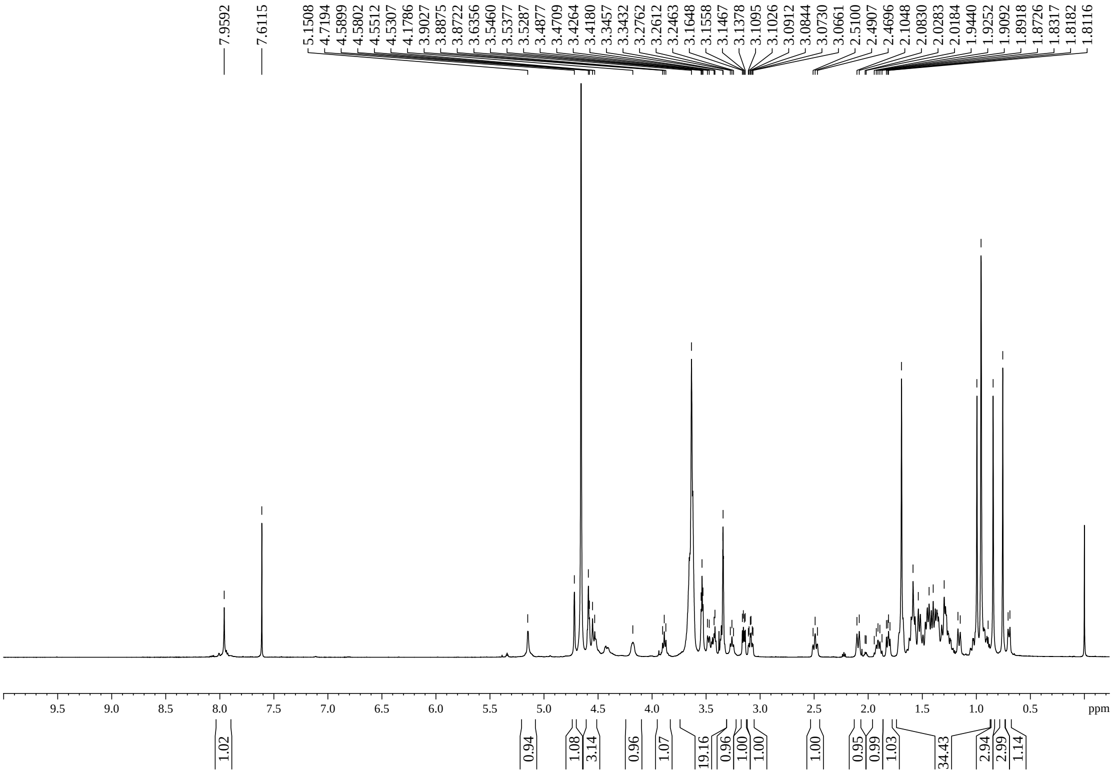

^{13}C NMR of compound **79**

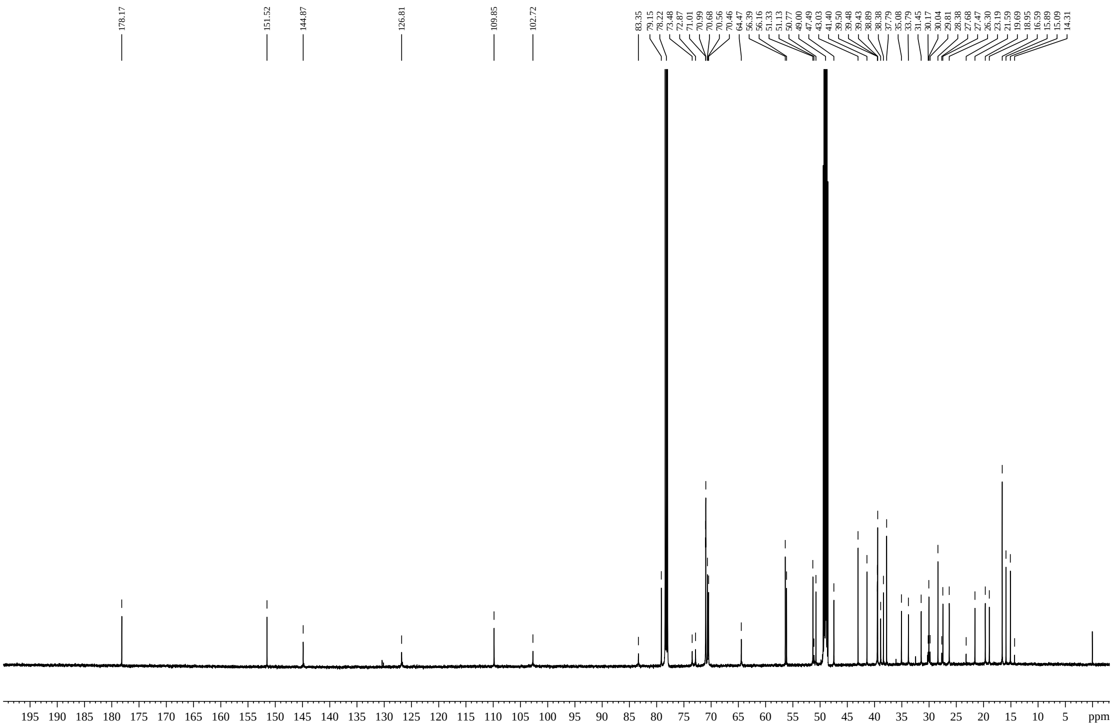

MALDI-TOF of compound **79**

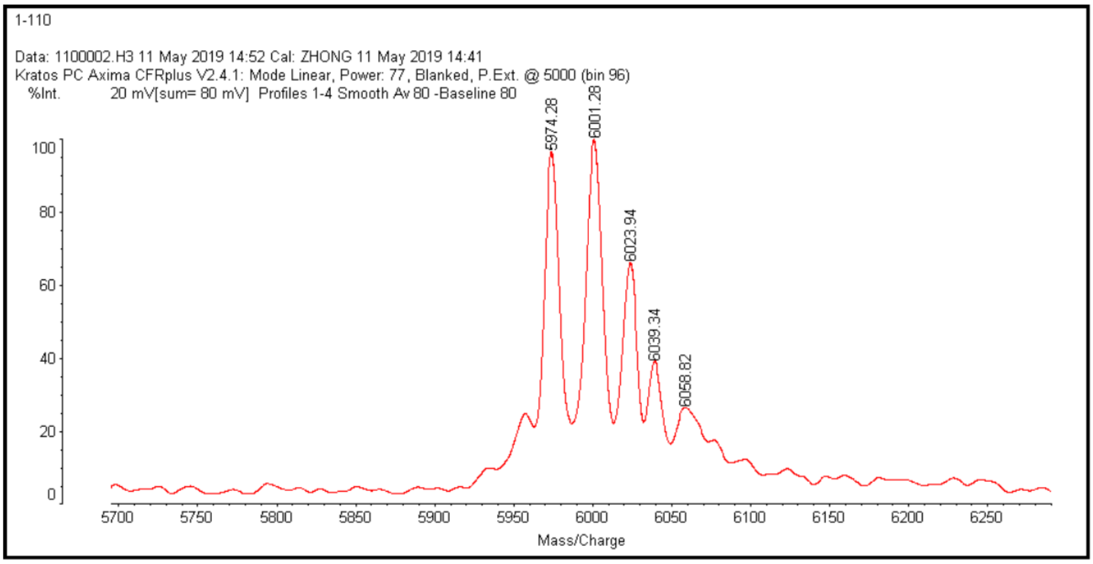

^{1}H NMR of compound **80**

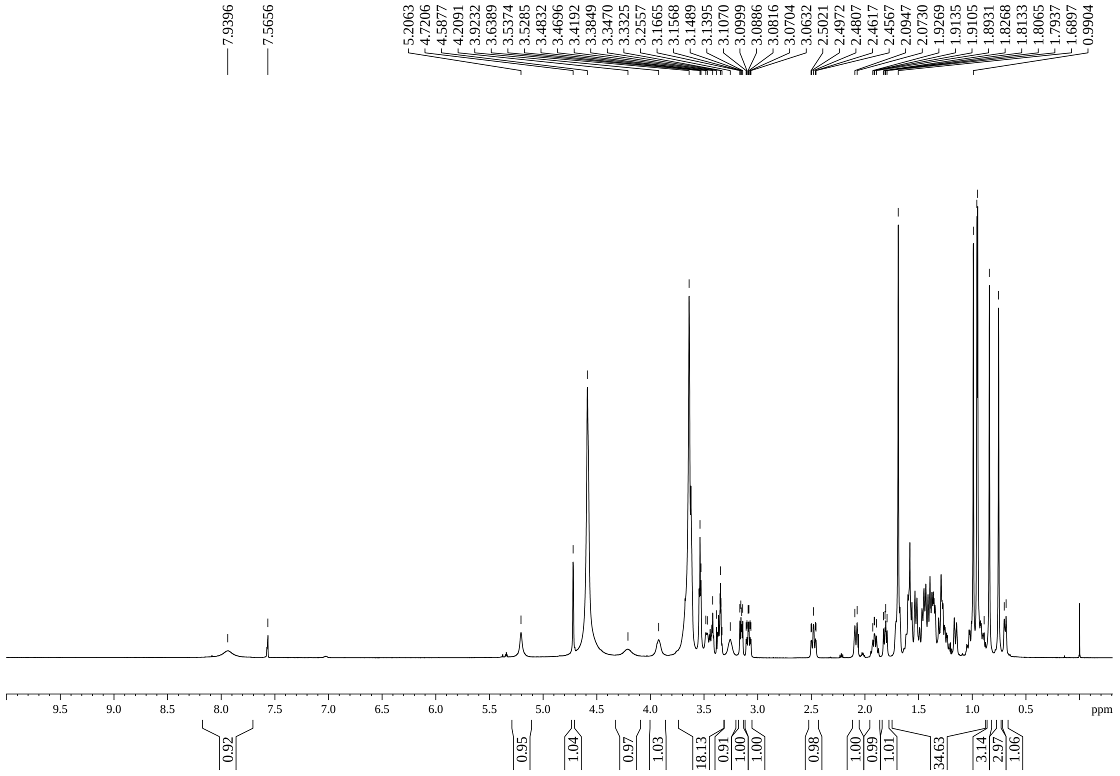

^{13}C NMR of compound **80**

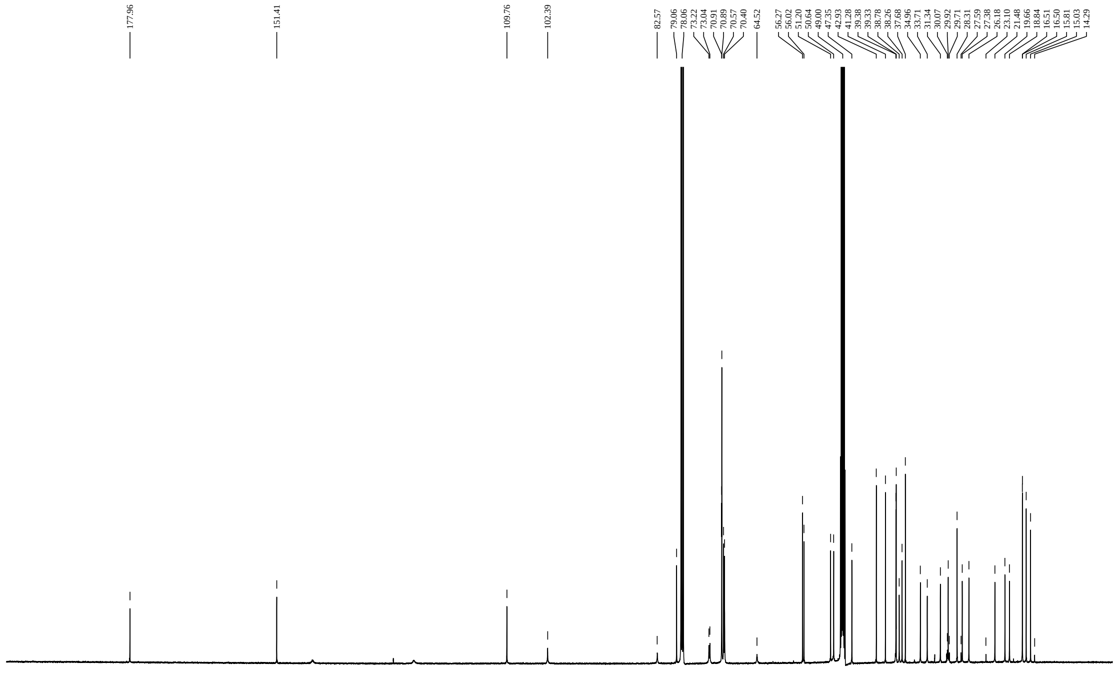

MALDI-TOF of compound **80**

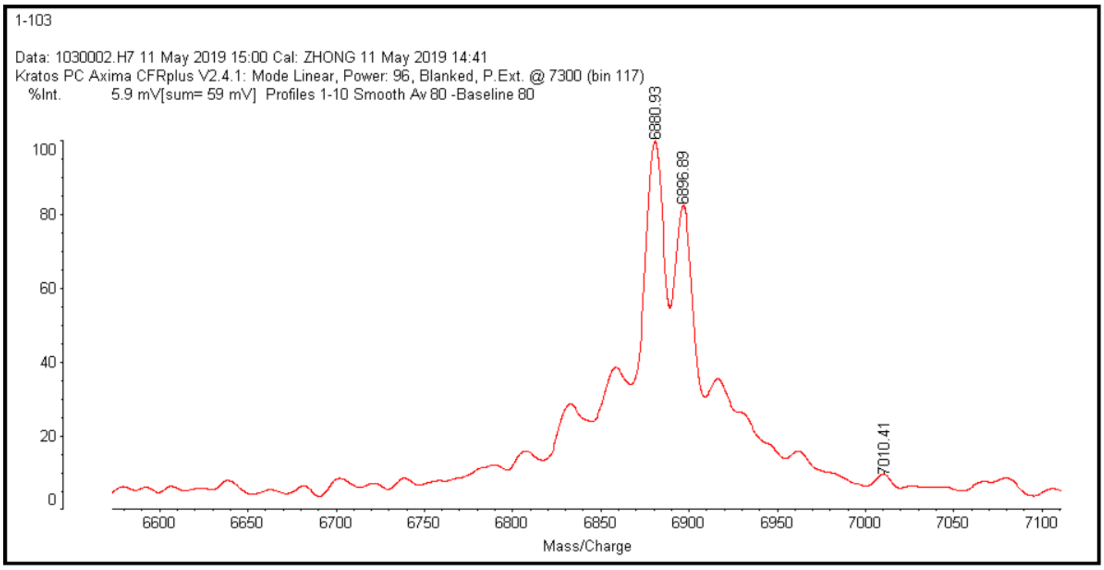

^{1}H NMR of compound **81**

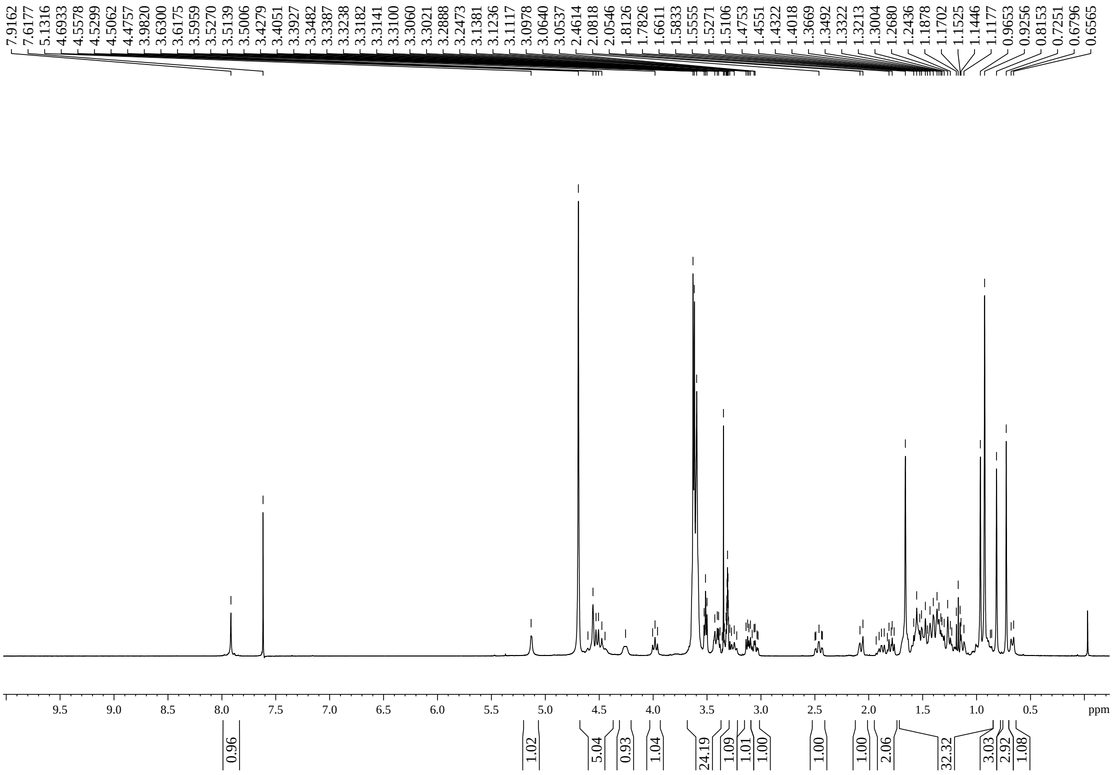

^{13}C NMR of compound **81**

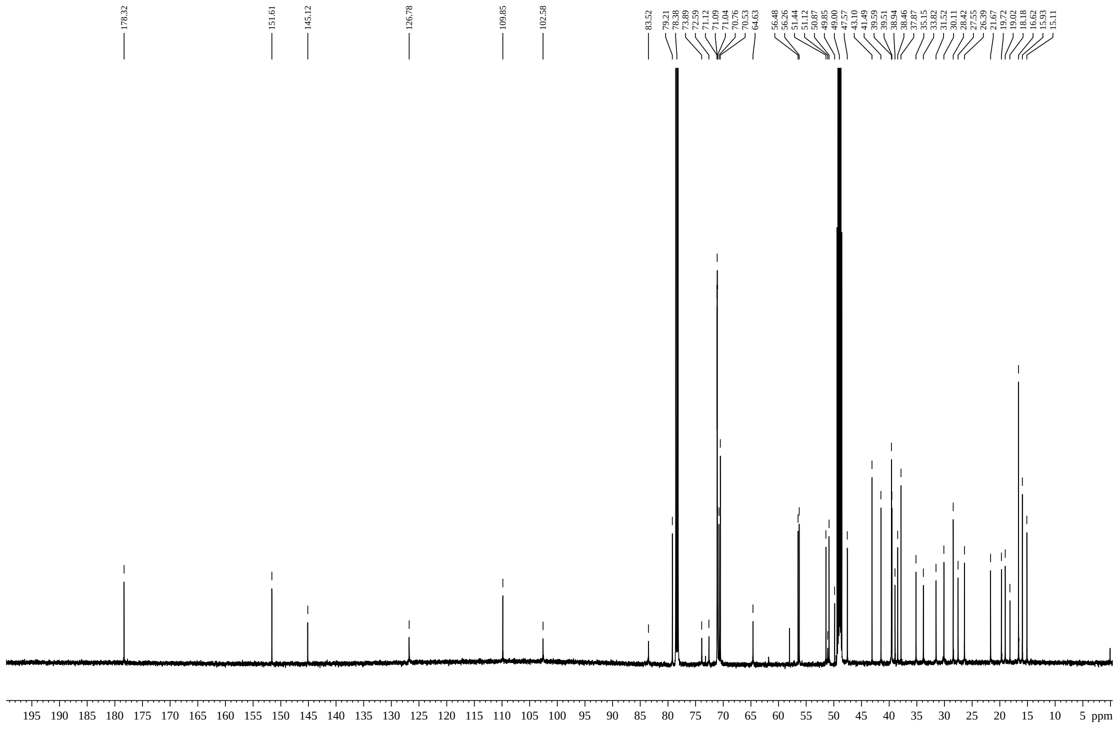

MALDI-TOF of compound **81**

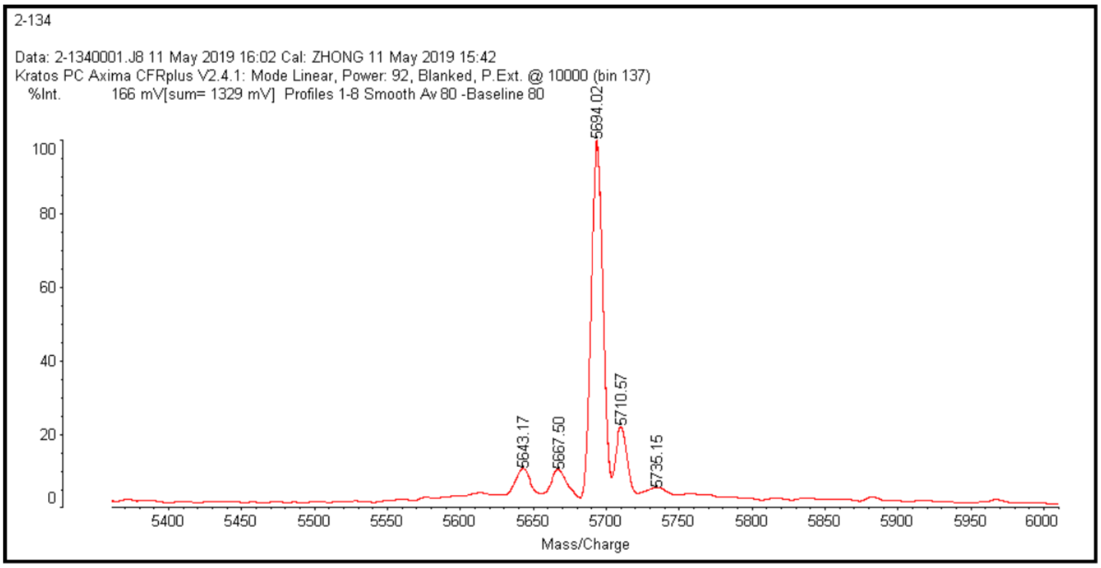

^{1}H NMR of compound **82**

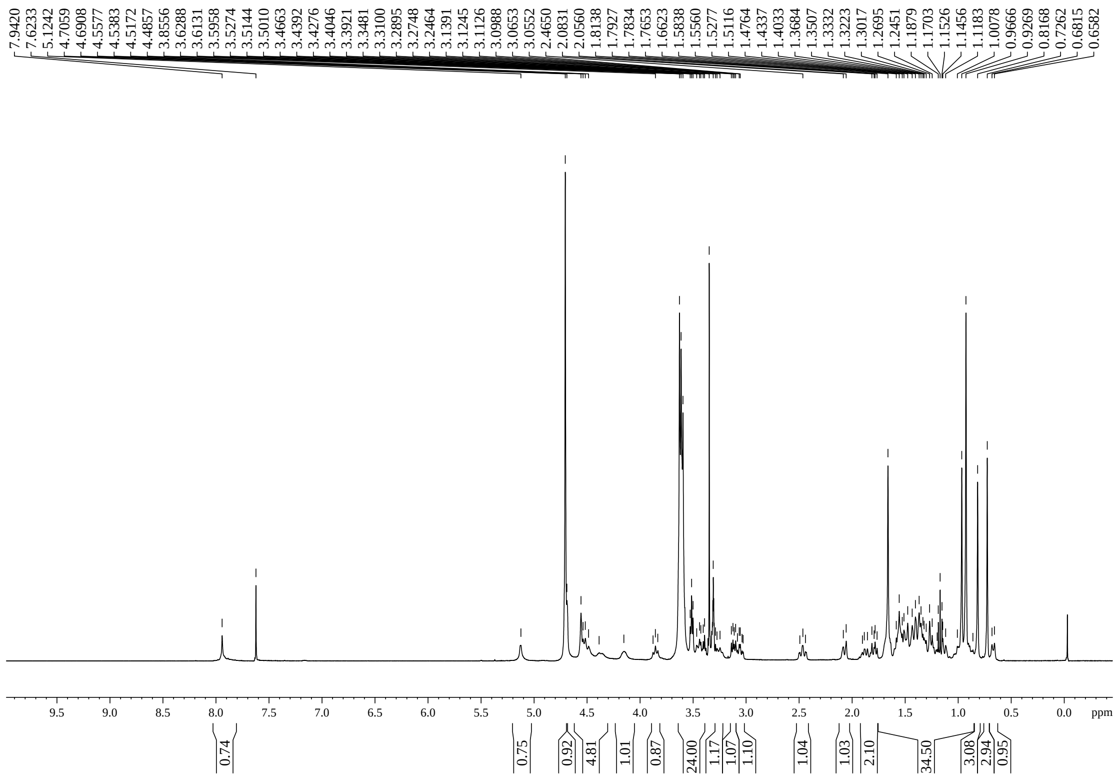

^{13}C NMR of compound **82**

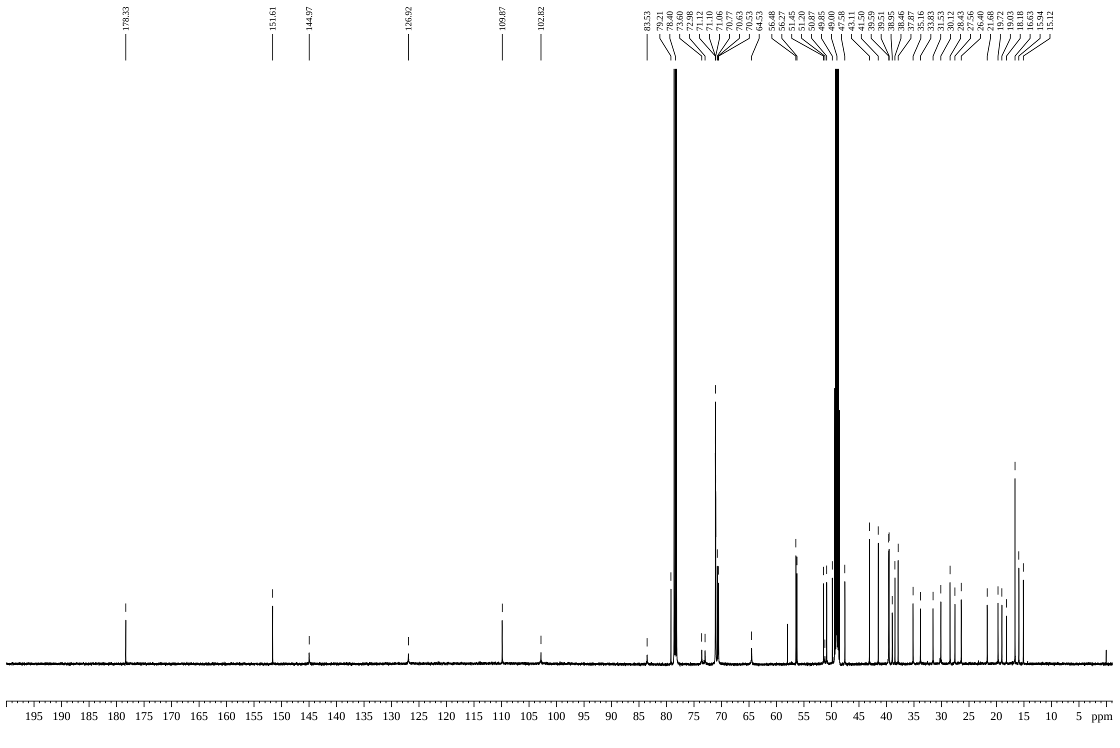

MALDI-TOF of compound **82**

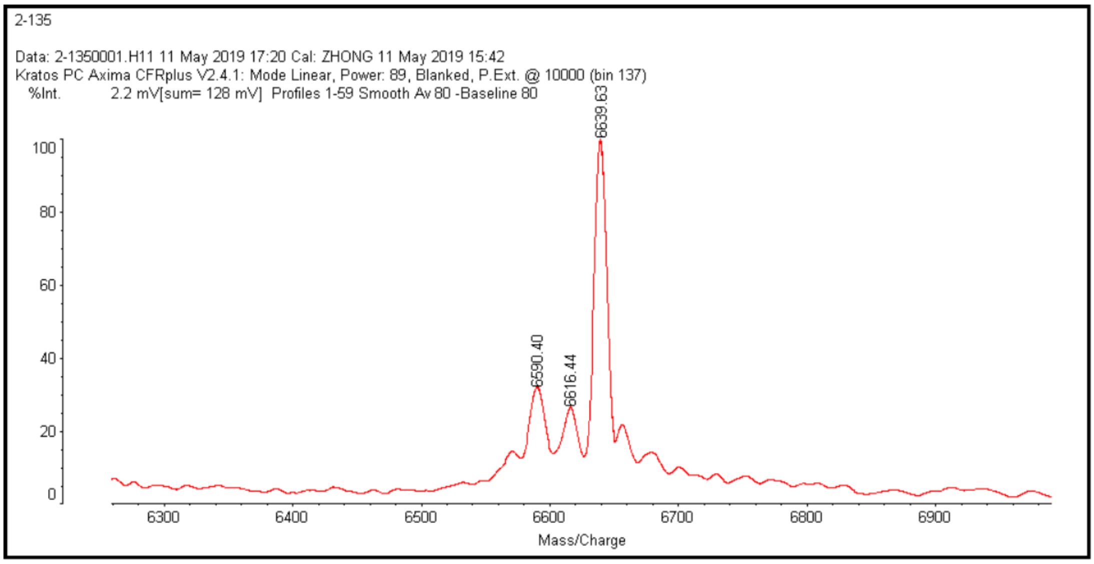

^{1}H NMR of compound **83**

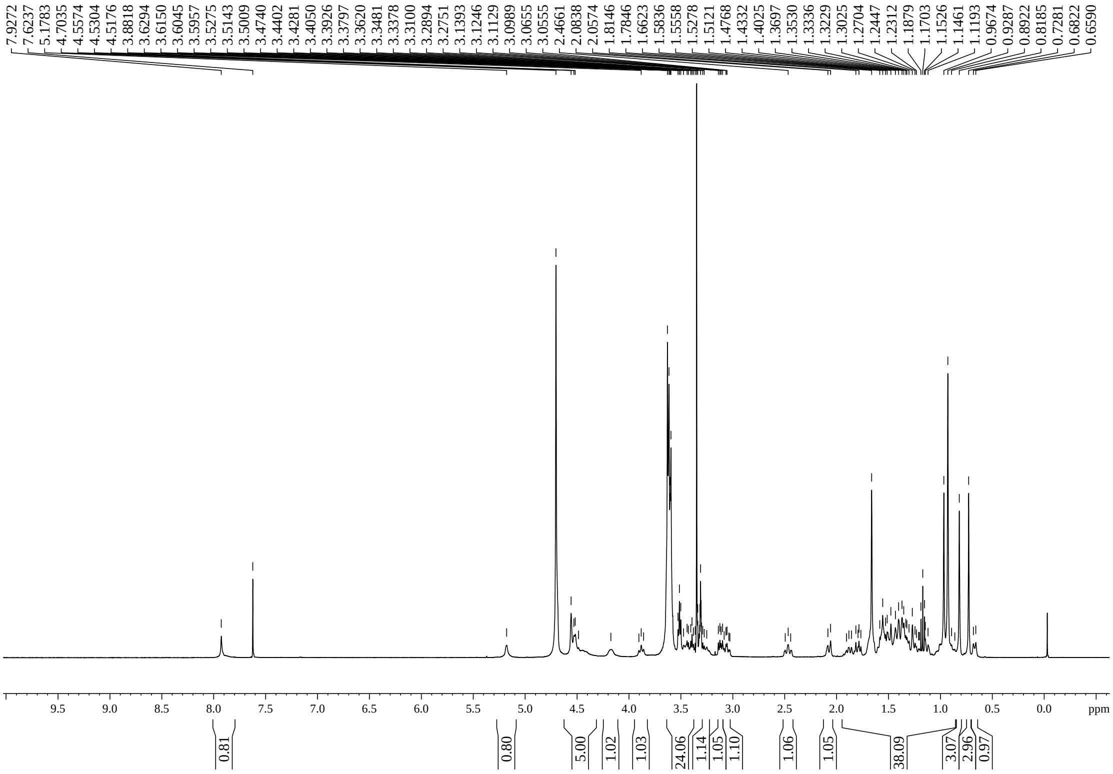

^{13}C NMR of compound **83**

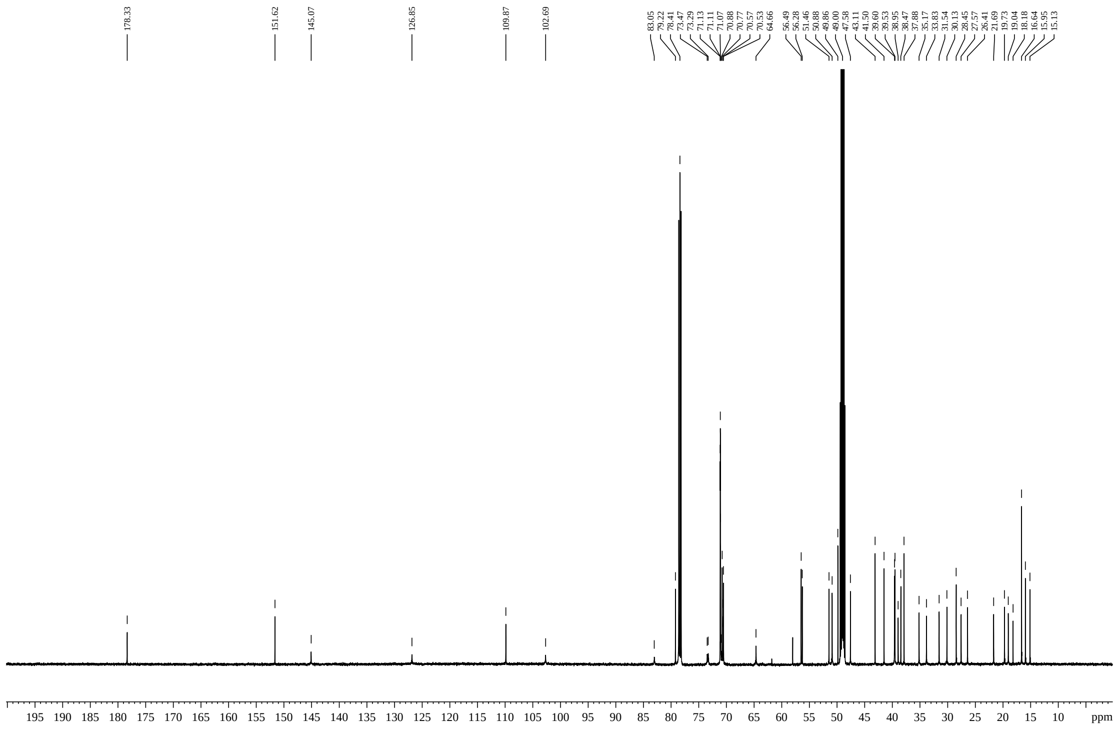

MALDI-TOF of compound **83**

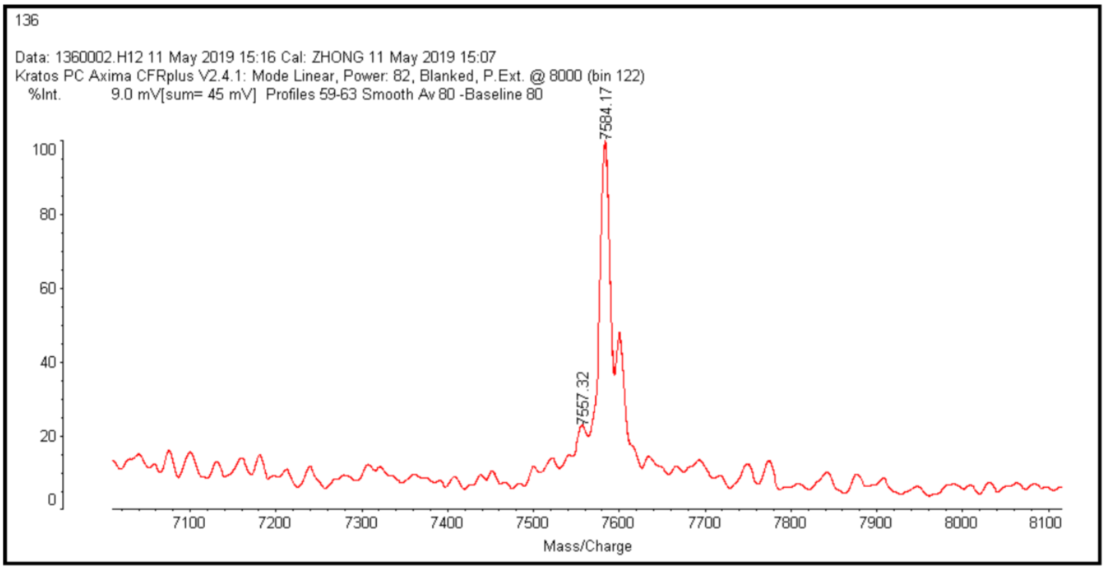

^{1}H NMR of compound **84**

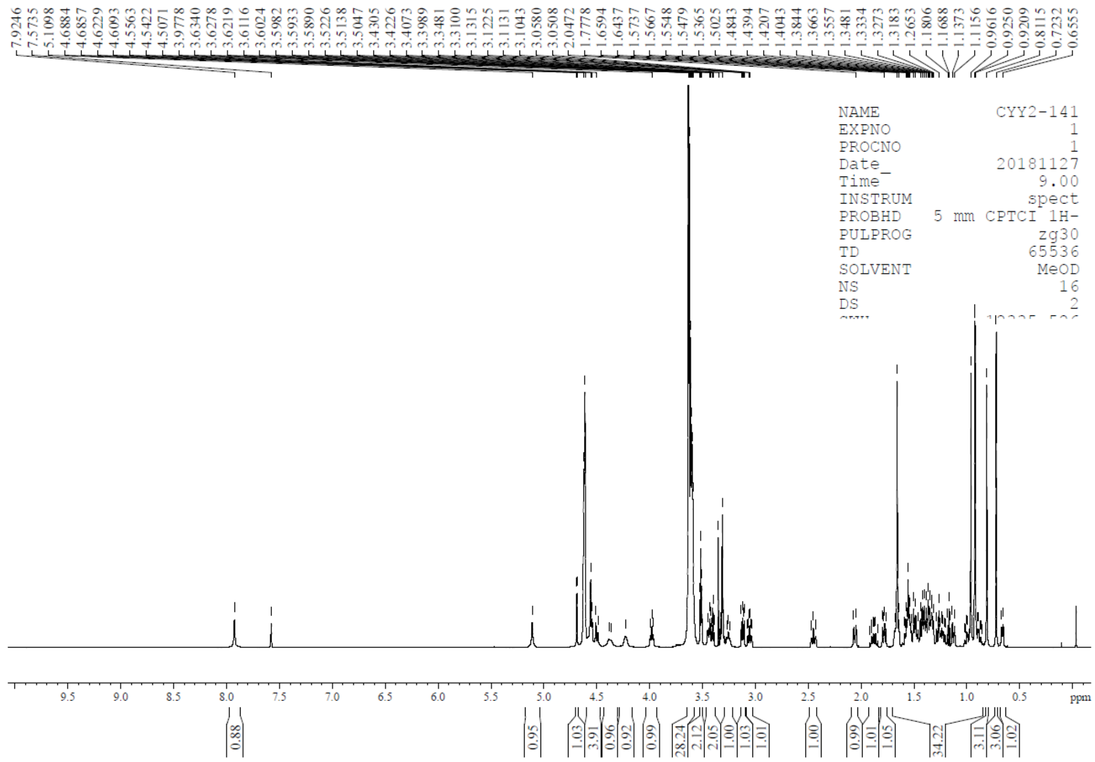

^{13}C NMR of compound **84**

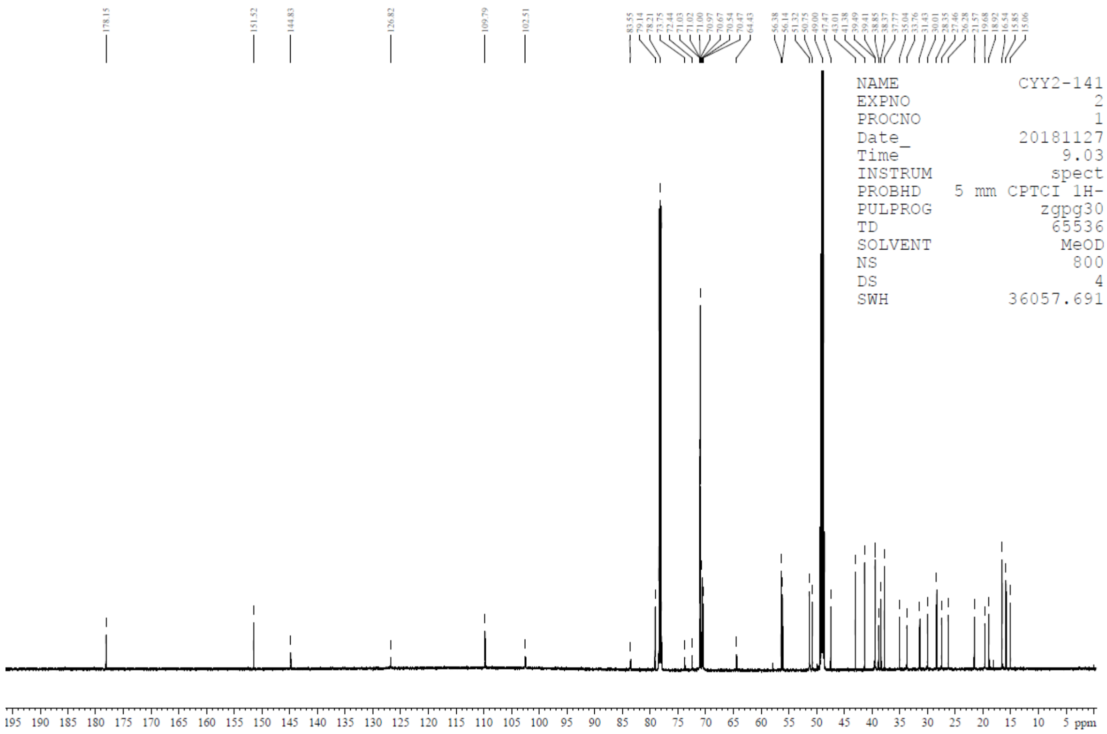

HRMS of compound **84**

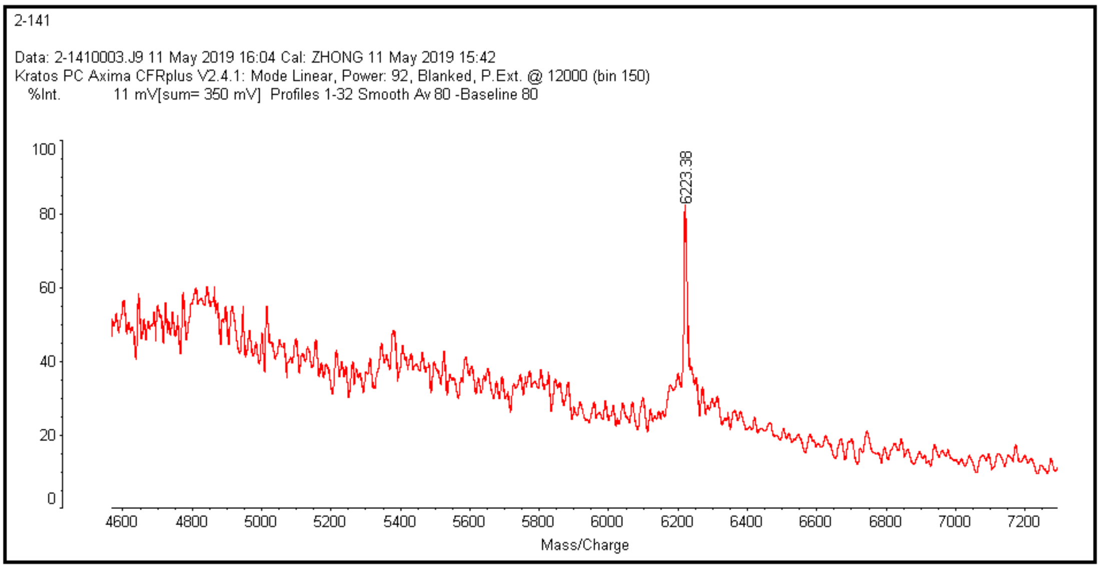

^{1}H NMR of compound **85**

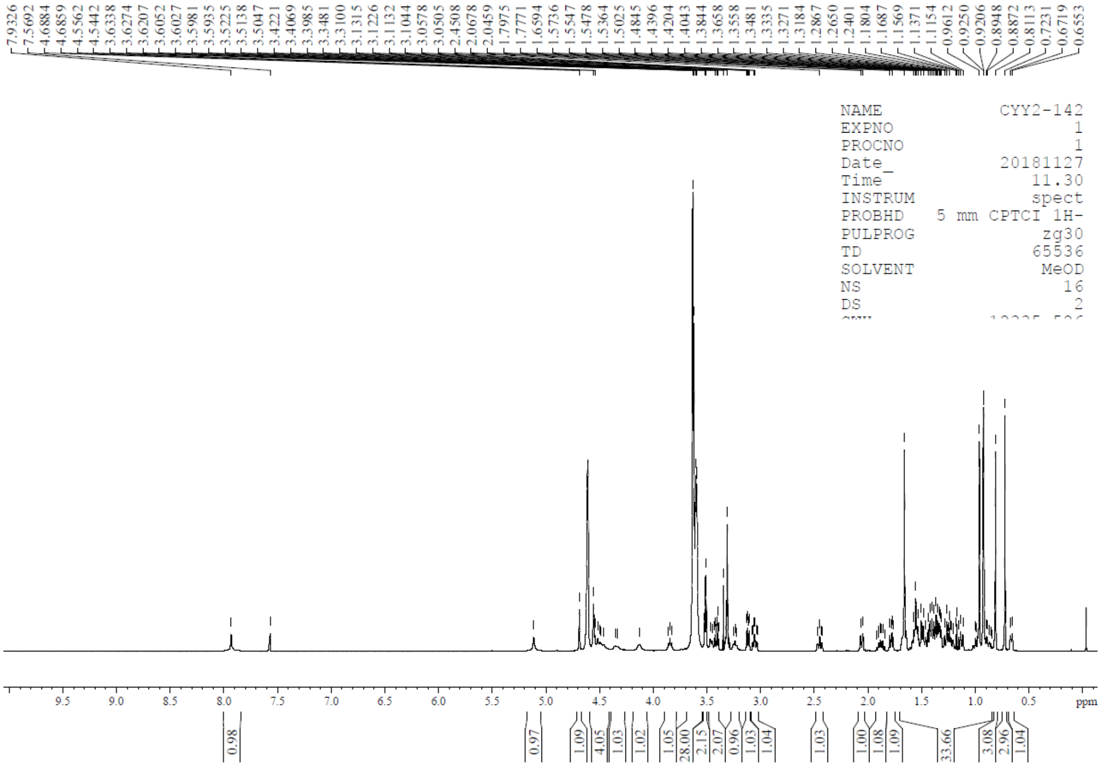

^{13}C NMR of compound **85**

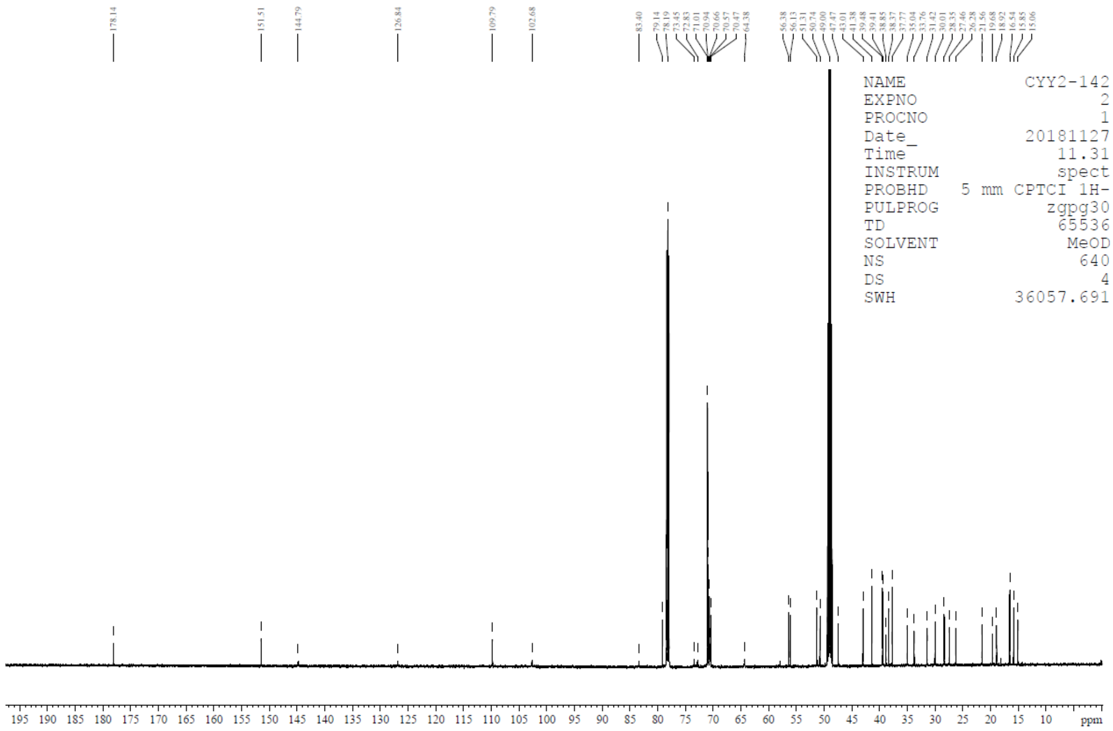

HRMS of compound **85**

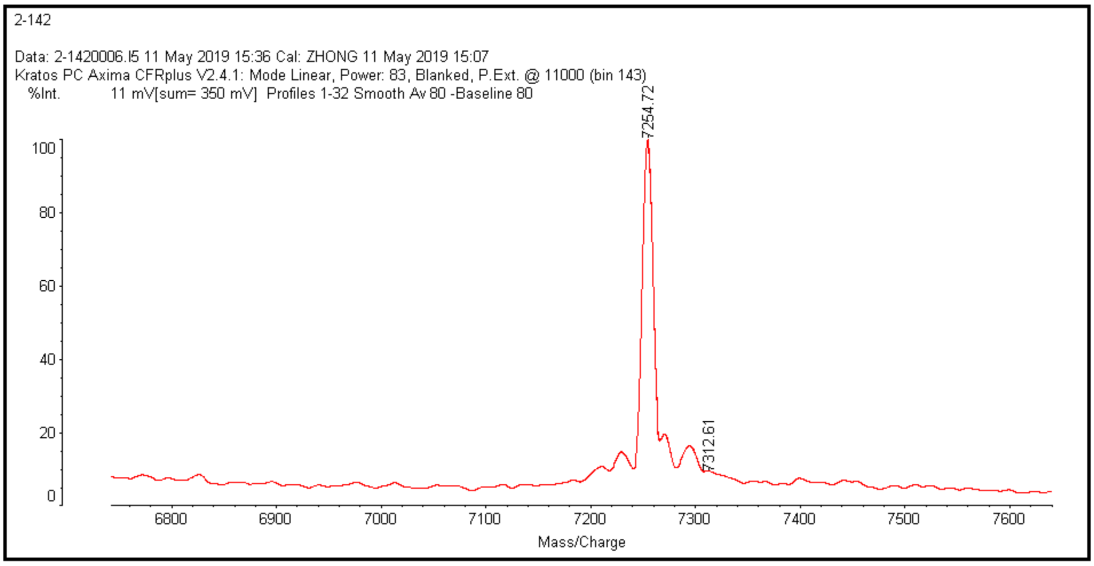

^{1}H NMR of compound **86**

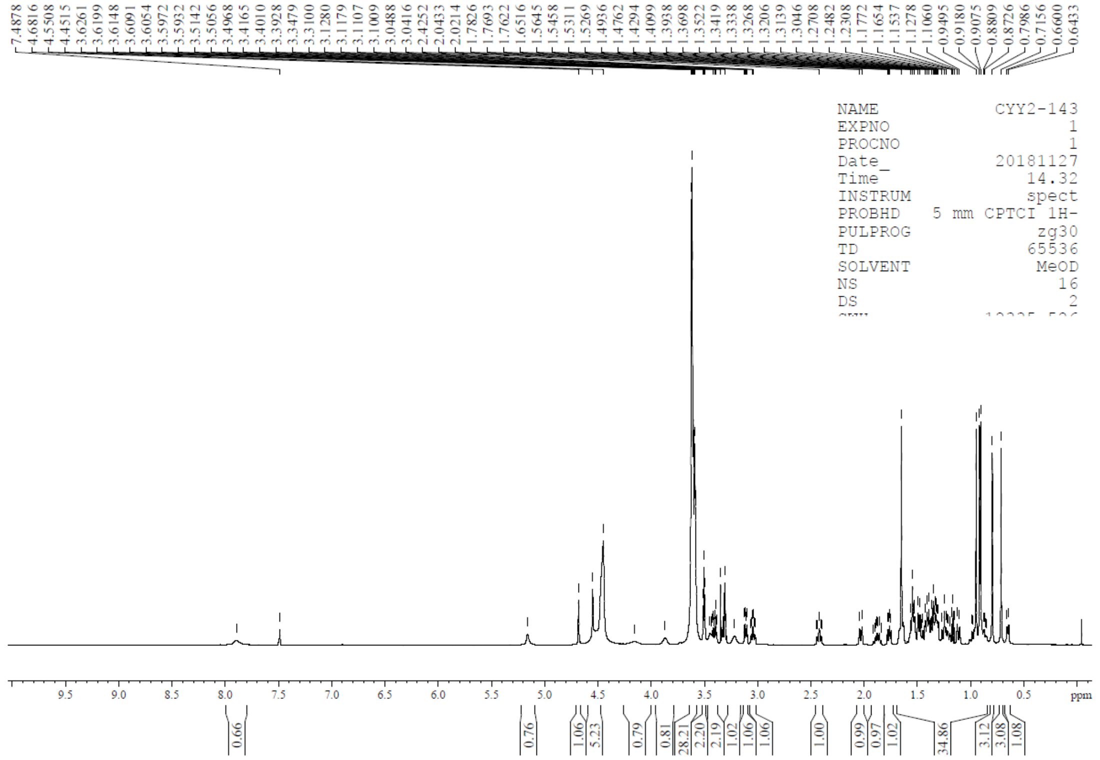

^{13}C NMR of compound **86**

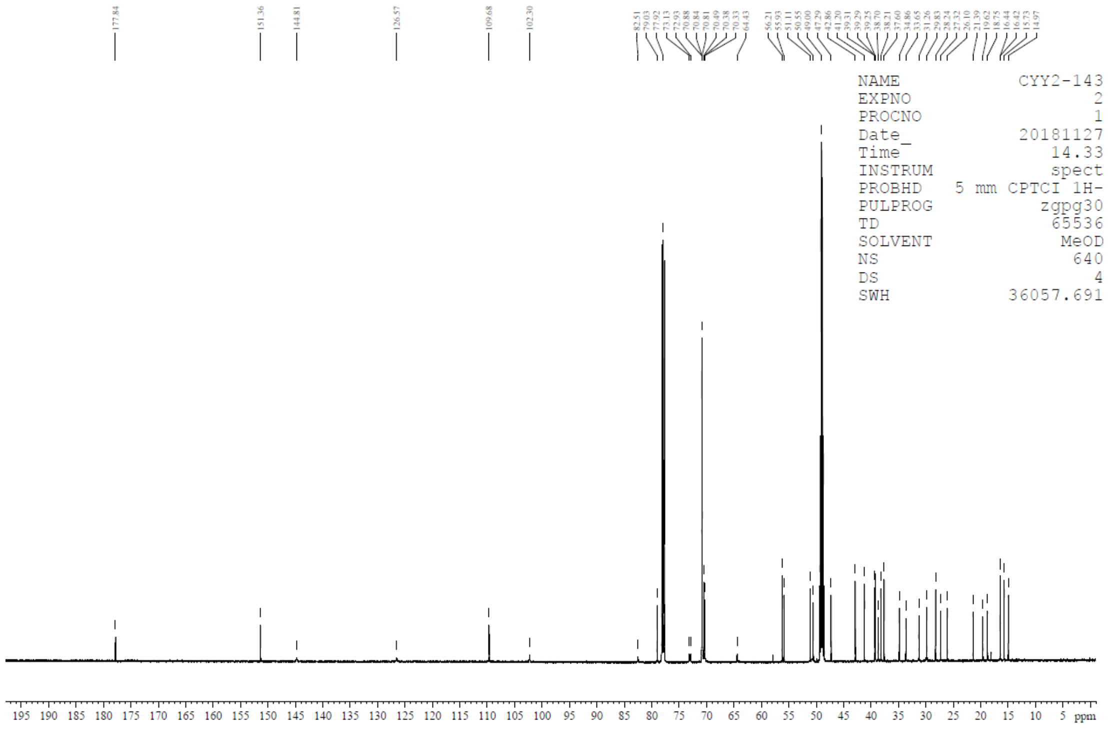

HRMS of compound **86**

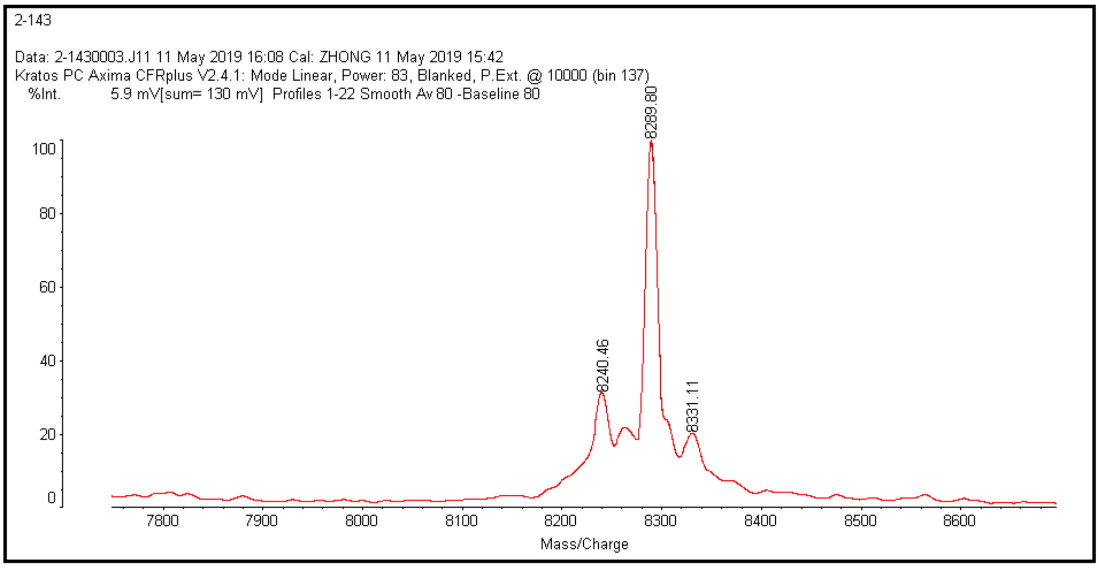

Supplement: Supplementary file 1 [file molecules-27-01163-s001.zip › molecules-1592077-supplementary.pdf]
